# Syntheses of 4-Indolylquinoline Derivatives via Reductive Cyclization of Indolylnitrochalcone Derivatives by Fe/HCl

**DOI:** 10.3390/molecules201219862

**Published:** 2015-12-15

**Authors:** Wen-Chang Chen, Chan-Chieh Lin, Veerababurao Kavala, Chun-Wei Kuo, Chia-Yu Huang, Ching-Fa Yao

**Affiliations:** Department of Chemistry, National Taiwan Normal University, 88, Sec. 4, Ting-Chow Road, Taipei 116, Taiwan; wcchen@taigenbiotech.com (W.-C.C.); ntnu21236@gmail.com (C.-C.L.); kavalaveeru@gmail.com (V.K.); cwkuo.water@gmail.com (C.-W.K.); tlotras@gmail.com (C.-Y.H.)

**Keywords:** Fe/HCl, reductive cyclization, 4-indolylquinoline, indolylchalcone

## Abstract

An easy and efficient procedure for the synthesis of 4-indolylquinoline derivatives is described. This process involves two steps, the first of which is the Michael addition of indole to nitrochalcones promoted by sulfamic acid under solvent free conditions and the second step is a reductive cyclization of the indolylnitrochalcone intermediates to 4-indolylquinoline derivatives by Fe/HCl in ethanol. In both steps, the reactions are clean and the yields of products are high.

## 1. Introduction

**Figure 1:**
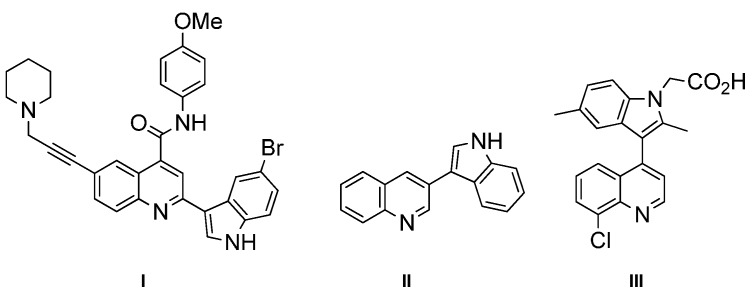
Bioactive indolylquinolines derivatives.

Indole and quinoline are two important class of structural scaffolds that are found in a vast number of natural products and pharmaceutically active compounds [1,2,3,4,5,6]. Compounds containing both indole and quinoline rings are called as indolylquinolines and are known to exhibit a wide variety of biological activities, including antibiotic, antimicrobial and antifungal activities [7,8,9,10,11,12]. Although different kinds of indolylquinoline derivatives are known in the literature, three types of indolylquinoline derivatives such as 2-indolylquinoline, 3-indolylquinoline, and 4-indolylquinoline are frequently found in many bioactive compounds. For example, 2-indolylquinoline [13,14] exhibit antistaphylococcal activities, 3-indolylquinolines [15,16,17,18] inhibit the activity of PDGF-RTK, 4-indolylquinolines [19,20,21,22] have been known for potential treatments for allergic rhinitis, asthma and other inflammatory diseases (Figure 1) [13,14].

A vast number of protocols are available for the synthesis of 2-indolylquinoline and 3-indolyl-quinoline derivatives [15,16,17,18], however, methods which describe the synthesis of 4-indolylquinoline derivatives are limited [19,20,21,22]. Marinelli and coworkers described a one-pot synthesis of 4-indolyl-quinoline derivatives from β-(2-aminophenyl)-α,β-ynones [23]. Recently, we reported a method for accessing 4-indolylquinoline derivatives through an inverse electron-demand aza-Diels-Alder reaction [24]. Some of these reported procedures required functionalized quinoline derivatives such as haloquinolines or indolylboronic acid derivatives and a few methods are associated with the use of expensive metal catalysts and starting materials. Hence, a simple and handy method for the synthesis of 4-indolylquinoline derivatives from easily available starting materials is desirable.

**Scheme 1:**
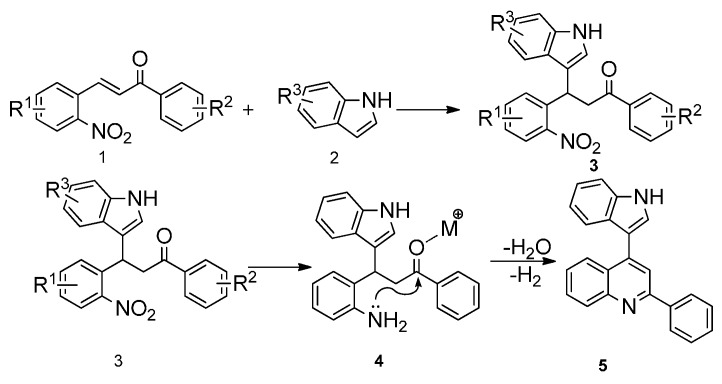
The proposed strategy for the synthesis of 4-indolyl quinoline derivatives.

For the past decade, we have been working on the use of reductive cyclization reactions [25,26,27,28,29,30,31,32] to generate a wide variety of nitrogen heterocycles, including indolylquinoline derivatives, 3,3′-biindoles, quinoline derivatives, 2*H*-1,4-benzoxazin-3-(4*H*)-ones, carbazolone derivatives, 2,3-disubstituted indole derivatives, acridinones and phenathridine derivatives by using Fe/AcOH as a reagent [33,34,35,36]. In continuation to our interest on reductive cyclization reactions, we proposed to synthesize 4-indolylquinoline derivatives in two steps starting from 2-nitrochalcone derivatives and indoles. The proposed strategy for the synthesis of 4-indolyl quinoline derivatives is shown in the Scheme 1. This strategy involves two steps: a Michael addition of indole to 2-nitrochalcone followed by the reductive cyclization.

## 2. Results and Discussion

To execute our strategy, we need to synthesize the Michael adducts of indoles and various 2-nitrochalcone derivatives. Although, several procedures describe the Michael addition reactions of indoles to chalcones [37,38,39,40,41,42], to our knowledge there is no procedure available for the Michael addition of 2-nitrochalcone with indole derivatives. On the other hand, we have reported Michael addition reactions of various 2-nitroalkenes and indoles using sulfamic acid as a catalyst under solvent free conditions to obtain the corresponding indolylnitroalkane derivatives in good to excellent yields [30]. We wished to adopt similar conditions to synthesize our starting materials, thus we tested the reaction of indole, 2-nitrochalcone, and sulfamic acid at the temperature of 90 °C under solvent free conditions. To our delight, the reaction was complete in 4 h and provided the corresponding 3-(1*H*-indol-3-yl)-3-(2-nitrophenyl)-1-phenylpropan-1-one derivative was obtained in good yield (Table 1, Entry 1).

Encouraged by this result, we applied these reaction conditions to synthesize various substituted indolylnitrochalcones. The reactions of indole with nitrochalcone derivatives with halogen group (F, Cl and Br) containing 2-nitrobenzalehydes and acetophenone proceeded quickly and afforded the desired products in good to excellent yields (Table 1, Entries 2–4). On the other hand, the reactions of indole with nitrochalcone derivatives derived from 2-nitrobenzaldehyde and *ortho* halogen group (Cl or Br) substituted acetophenones took place smoothly to provide the corresponding Michael adducts in quantitative yields.

**Table 1:** Michael addition of various 2-nitrochalcones and indole. 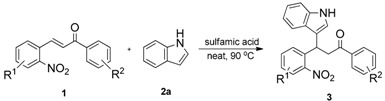

Entry ^a^	Nitrochalcone	Indole	Product	Time (h)	Yield % ^b^
1	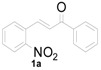	**2a**	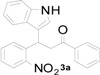	4.0	83
2	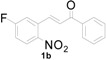	**2a**	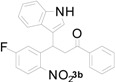	0.5	76
3	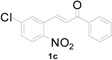	**2a**	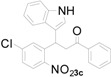	1.0	89
4	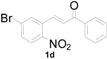	**2a**	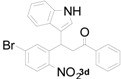	2.5	99
5	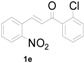	**2a**	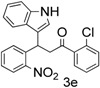	2.5	99
6	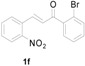	**2a**	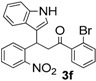	2.5	99
7	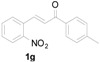	**2a**	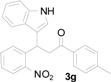	2.0	99
8	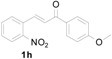	**2a**	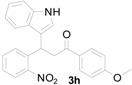	2.5	99
9	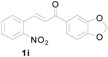	**2a**	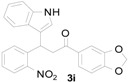	2.5	93
10	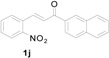	**2a**	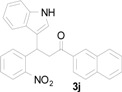	3	98
11	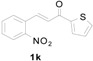	**2a**	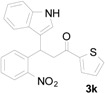	10	93

^a^ All reactions were carried out by using 1.0 equiv. of **1** and 1.2 equiv. of **2a** in the presence of 50 mol % of sulfamic acid; ^b^ Isolated yields.

Next, we investigated the reactions of unsubstituted nitrochalcone and various substituted indoles (Table 2).

**Table 2:** Michael addition of 2-nitrochalcone (**1a**) and various indoles. 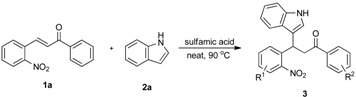

Entry ^a^	Nitrochalcone	Indole	Product	Time (h)	Yield % ^b^
1	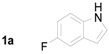	**2b**	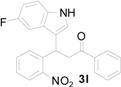	2.0	81
2	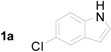	**2c**	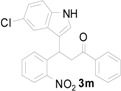	1.5	85
3	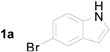	**2d**	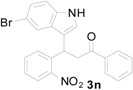	3.0	89
4	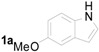	**2e**	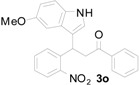	1.0	99
5	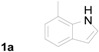	**2f**	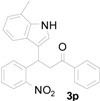	1.0	90
6	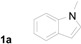	**2g**	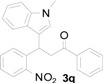	1.0	97
7	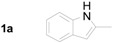	**2h**	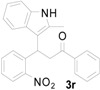	2.0	70
8	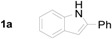	**2i**	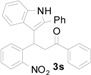	72	65

^a^ All reactions were carried out by using 1.0 equiv. of **1** and 1.2 equiv. of **2a** in the presence of 50 mol % of sulfamic acid; ^b^ Isolated yields.

When nitrochalcone was treated with electron-withdrawing group containing indoles such as 5-fluoroindole, 5-chloroindole and 5-bromoindole, the reactions produced the corresponding Michael adducts in good yields. On the other hand, the reactions of nitrochalcone and electron-donating indoles such as 5-methoxyindole and 6-methylindole provided its corresponding Michael adducts in excellent yields. Next, the reaction of *N*-methylindole and 2-nitrochalcone afford the desired Michael adduct in excellent yield. Further, the reaction of 2-nitrochalcone with 2-phenylindole or 2-methylindole afforded the corresponding Michael adducts in moderate yields. Moreover, the reactions of 2-nitrochalcone with 2-phenylindole or 2-methylindole took longer to go to completion (Table 2).

After the preparation of various substituted 3-(1*H*-indol-3-yl)-3-(2-nitrophenyl)-1-phenyl-propan-1-one derivatives, we then focused on the reductive cyclization of these compounds to 4-indolylquinoline derivatives. Initially, we treated 3-(1*H*-indol-3-yl)-3-(2-nitrophenyl)-1-phenyl-propan-1-one with the standard reductive cyclization agent Fe/AcOH [9] (Table 3, entry 1). Under these conditions the reaction afforded two compounds. From the ^1^H- and ^13^C-NMR spectral data and mass spectral analysis, it was revealed that the major product was the 4-indolylquinoline derivative and the minor product was the indole-eliminated 2-phenylquinoline derivative.

Then, we tried Zn as reducing reagent (Table 3, entries 2 and 3), but the results were not encouraging. As it is reported in the literature [43,44] that Fe/HCl is an efficient reductive cyclizing agent, next, we used Fe/HCl in EtOH (Table 3, entry 4) for this transformation. To our delight, the reaction produced the indolylquinoline derivative in excellent yield without any of the minor product. Further, when the reaction was performed using a mixed solvent such as ethanol and water (1:1), the reaction afforded an excellent yield of the 4-indolylquinoline derivative (Table 3, entry 5). However, the reaction time was longer in this case. Furthermore, when the reaction was performed in methanol it resulted in a decreased yield of the desired product (Table 3, entry 6). From the optimization results, the reaction condition using Fe/HCl in ethanol at reflux temperature (Table 3, entry 5) were found to be the best condition for the synthesis of 4-indolylquinoline derivatives from the corresponding 3-(1*H*-indol-3-yl)-3-(2-nitrophenyl)-1-phenylpropan-1-one derivatives (Table 3).

**Table 3:** Optimization studies for reductive cyclization of 3-(1H-indol-3-yl)-3-(2-nitrophenyl)-1-phenylpropan-1-one (**3a**). 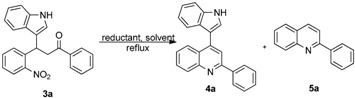

Entry ^a^	Reductant	Solvent	Time (h)	Yield of 4a (%) ^b^	Yield of 5a (%) ^b^
1	Fe	AcOH	0.5	72	24
2	Zn	AcOH	10	16	21
3	Zn ^c^	THF–H_2_O ^d^	2.0	0	0
4	Fe ^e^	EtOH	1.0	90	0
5	Fe ^e^	EtOH–H_2_O ^f^	4.0	88	0
6	Fe ^e^	MeOH	10	57	0

^a^ **3a** (1.0 equiv.), metal (6.0 equiv.), solvent (10 mL); ^b^ Isolated yields; ^c^ NH_4_Cl (1.1 equiv.); ^d^ THF–H_2_O (2:1); ^e^ HCl (1.0 equiv.); ^f^ EtOH–H_2_O (4:1).

Having the optimized reaction conditions in hand, we then investigated the scope and limitations of this protocol. As shown in Table 4, 3-(1*H*-indol-3-yl)-3-(2-nitrophenyl)-1-phenylpropan-1-one (**3a**) reacted under optimized reaction conditions to produce the 4-indolyl-quinoline derivatives in excellent yield.

Under the present reaction conditions, the substrates containing electron-withdrawing groups (F, Cl and Br) in the nitrochalcone part of 3-(1*H*-indol-3-yl)-3-(2-nitrophenyl)-1-phenylpropan-1-one reacted well and afforded the corresponding 4-indolyl-quinoline derivatives in good yields, while the reactions of the substrates possessing electron-donating groups provided the desired 4-indolylquinoline derivatives in excellent yields. Moreover, the substrate bearing a naphthalene ring also provided the corresponding product in 93% yield under the present reaction conditions. Next, the 3-(1*H*-indol-3-yl)-3-(2-nitrophenyl)-1-phenylpropan-1-one derivative containing a thiophene group was also reacted under the present reaction conditions to obtain the corresponding indolylquinoline derivative in excellent yield (Table 4).

**Table 4:** Reductive cyclization of substituted 3-(1*H*-indol-3-yl)-3-(2-nitrophenyl)-1-phenylpropan-1-one derivatives derived from various 2-nitrochalcone and indoles. 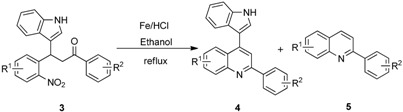

Entry ^a^	Indolylnitrochalcone	Product	Time (h)	Yield (%) ^b,c^
1	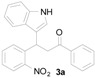	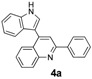	4.0	83
2	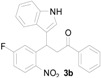	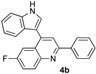	0.5	76
3	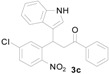	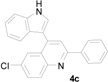	1.0	89
4	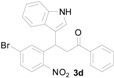	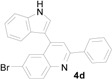	2.5	99
5	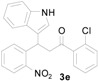	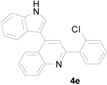	2.5	99
6	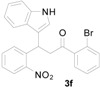	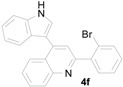	2.5	99
7	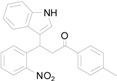	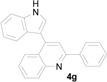	2.0	99
8	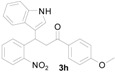	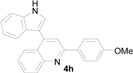	2.5	99
9	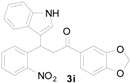	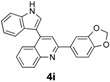	2.5	93
10	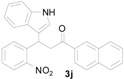	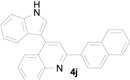	3	93
11	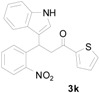	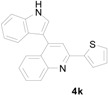	10	93

^a^ Condition: **3** (1.0 equiv.), Fe (6.0 equiv.), HCl (1.0 equiv.), EtOH (10 mL); ^b^ Isolated yields; ^c^ no trace of **5** was observed.

Next, we investigated the reactions of Michael adducts derived from nitrochalcone and various indoles. As shown in the Table 5, the 3-(1*H*-indol-3-yl)-3-(2-nitrophenyl)-1-phenylpropan-1-one derivatives derived from electron poor indoles (fluoro-, chloro-, and bromoindoles) underwent a smooth reductive cyclization to afford the desired 4-indolylquinoline derivatives in excellent yield, whereas, the substrates derived from the electron rich indoles produced the corresponding indolylquinoline derivatives in slightly lower yields than those of electron poor indoles. It is notable that the reactions of the substrate obtained from 2-phenyl or 2-methylindole provided the corresponding 4-indolylquinoline derivative in moderate yield along with a substantial amount of the indole cleaved product as byproduct. These results show that steric hindrance influences the elimination of indole to produce the indole-cleaved product (Table 5).

To further examine the influence of steric hindrance, we investigated the reactions of the 3-(1*H*-indol-3-yl)-3-(2-nitrophenyl)-1-phenylpropan-1-one substrates containing a methyl group adjacent to the nitro group as well the substrates derived from 2,5-dimethylindoles. As shown in the Table 6, steric hindrance adjacent to the nitro group has less influence on the reaction outcome, as the substrate **3t** gave the corresponding indolylquinoline in good yield along with traces of the indole cleaved product. However, when the substrate **3u** derived from 2,5-dimethylindole was treated under the present reaction conditions, we obtained 66% of indolylquinoline derivative and 30% of indole-cleaved product. Furthermore, we obtained only indole-cleaved product, when the substrate **3v** containing a methyl group adjacent to the nitro as well as the second indole position was used. It is important to note that the reactions took longer when the methyl group was adjacent to the nitro group as in case of entries 2, 3 in Table 6.

**Table 5:** Reductive cyclization of 3-(1*H*-indol-3-yl)-3-(2-nitrophenyl)-1-phenylpropan-1-one derivatives derived from various indoles and 2-nitrochalcone. 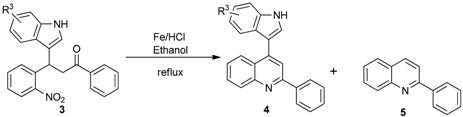

Entry ^a^	Indolylnitrochalcone	Product	Time (h)	Yield % ^b^
1	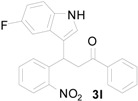	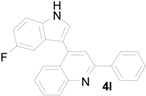	1.5	99
2	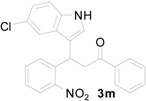	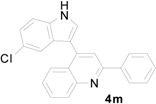	1.5	99
3	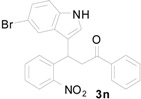	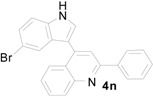	1.5	99
4	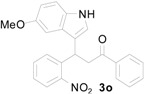	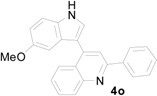	1.0	82
5	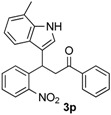	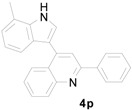	1.0	85
6	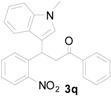	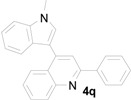	1.0	83
7	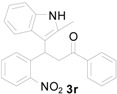	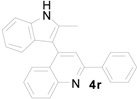	3.0	66 ^c^
8	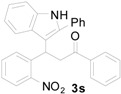	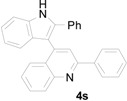	6.0	57 ^d^

^a^ Condition: **3** (1.0 equiv.), Fe (6.0 equiv.), HCl (1.0 equiv.), EtOH (10 mL); ^b^ Isolated yields; ^c^ 17% of product 5 formed along with 4r (66%); ^d^ 30% of 5 formed along with 4s (57%)

**Table 6:** Reductive cyclization of sterically hindered 3-(1*H*-indol-3-yl)-3-(2-nitrophenyl)-1-phenylpropan-1-one derivatives. 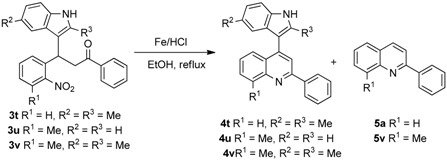

Entry ^a^	Substrate	Solvent	Time (h)	Yield of 4 (%) ^b^	Yield of 5 (%) ^b^
1	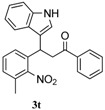	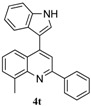	24	85	8
2	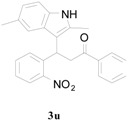	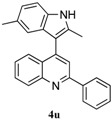	1.0	66	30
3	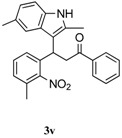	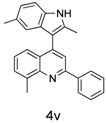	24	0	70

^a^ Condition: **3** (1.0 equiv.), Fe (6.0 equiv.), HCl (1.0 equiv.), EtOH (10 mL); ^b^ Isolated yields.

**Scheme 2:**
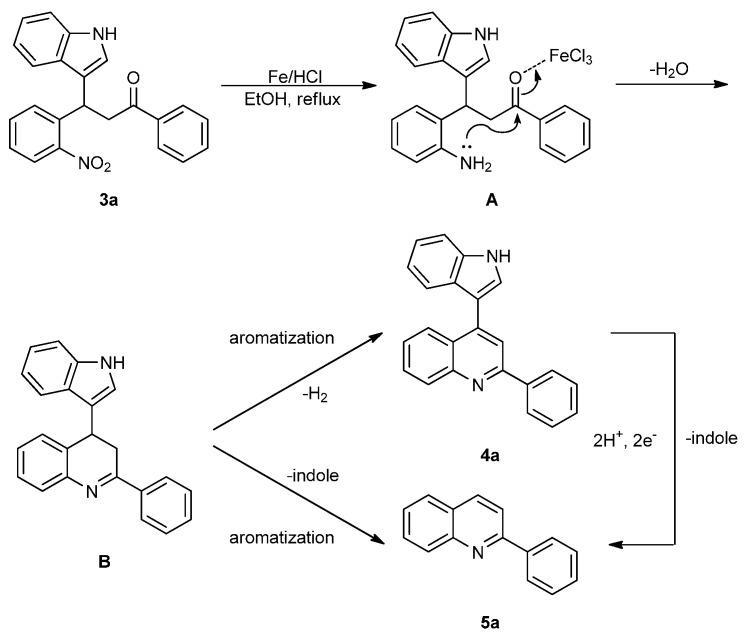
Mechanistic route for the reductive cyclization of 3-(1*H*-indol-3-yl)-3-(2-nitrophenyl)-1-phenylpropan-1-one derivatives.

A reaction mechanism for the formation of the indolylquinoline as well as the indole-cleaved product from 3-(1*H*-indol-3-yl)-3-(2-nitrophenyl)-1-phenylpropan-1-one (**3a**) is proposed based on our previous work (Scheme 2). Initially, the nitro group of the 3-(1*H*-indol-3-yl)-3-(2-nitrophenyl)-1-phenylpropan-1-one (**3a**) is reduced to an amino group by Fe/HCl, then it attacks the carbonyl group in the presence of FeCl_3_ in the solvent, forming a dihydroquinoline intermediate. The dihydroquinoline intermediate compound is unstable, and undergoes aromatization by the loss of hydrogen or the indole moiety to give either **4a** or **5a**. Besides, we also anticipate that indolylquinoline derivative **4a** may also undergo a slow decomposition to indole-cleaved product **5a** through reductive elimination. To explore this reductive elimination possibility, the indolylquinoline **4a** was treated with Fe/AcOH under the identical conditions used in the preparation of indolylquinoline derivatives. We obtained around 14% of indole-cleaved product along with 82% of the unchanged indolylquinoline **4a** after 24 h. The result was similar even when reaction was conducted with Fe/HCl used as reagent in ethanol. However, when the reaction was performed with FeCl_3_, the indolylquinoline was unchanged. From these experiments, we cannot exclude this route for the formation of the indole-cleaved product.

## 3. Experimental Section

### 3.1. General Information

All chemicals were purchased from various commercial sources and used directly without further purification. Analytical thin-layer chromatography was performed using E. Merck (New York, NY, USA) silica gel 60F glass plates and E. Merck silica gel 60 (230–400 mesh) was used in flash chromatography separations. MS were measured by a JMS-HX110 spectrometer (JEOL, Hsinchu, Japan). HRMS spectra were recorded using ESI-TOF or EI+ mode or FAB+. ^1^H- (400 MHz) and ^13^C-NMR (100 MHz) spectra were recorded with an Advance EX 400 MHz spectrometer (Bruker, San Francisco, CA, USA). Chemical shifts are reported in parts per million (δ) using TMS as an internal standard and coupling constant were expressed in hertz. IR spectra were performed on a 100 series FT-IR instrument (Perkin Elmer, Waltham, MA, USA). Melting points were recorded using a capillary melting point apparatus (Electrothermal, Staffordshire, UK) and are uncorrected. All substrates were prepared using literature procedures.

### 3.2. General Procedure for the Reaction of Indoles with Nitrochalcones to give Products ***3a***–***3v***

The mixture of nitrochalcone (2.0 mmol), indole (2.2 mmol), and sulfamic acid (1 mmol, 0.5 eq.) was heated at 90 °C until the complete consumption of starting materials, which was monitored by TLC. Then, the reaction mixture was allowed to cool to room temperature, diluted with ethyl acetate (20 mL) and washed with water, followed by brine. The organic layer was separated, dried over anhydrous MgSO_4_, and concentrated in vacuum to obtain the crude product. The residue was purified by flash column chromatography (petroleum ether/ethyl acetate) to obtain the desired products **3a**–**3v**. (The ^1^H-, ^13^C-NMR spectra of the compounds (**3a**–**3v**) was showed in supplementary).

*3-(1H-Indol-3-yl)-3-(2-nitrophenyl)-1-phenylpropan-1-one* (**3a**): Pale crystalline yellow solid (crystallized from ethyl acetate and hexane) with a melting point of 176–178 °C; IR (KBr): 3320, 3050, 1684, 1525, 1354, 741 cm^−1^. ^1^H-NMR (DMSO-*d*_6_) δ 10.99 (s, 1H), 8.00 (d, *J* = 7.5 Hz, 2H), 7.83 (d, *J* = 8.0 Hz, 1H), 7.61 (d, *J* = 6.3 Hz, 2H), 7.54–7.48 (m, 3H), 7.42–7.36 (m, 2H), 7.38–7.33 (m, 2H), 7.04 (t, *J* = 7.3 Hz, 1H), 6.90 (t, *J* = 4.7 Hz, 1H), 5.45 (t, *J* = 6.8 Hz, 1H), 4.04 (dd, *J* = 18.0, 6.4 Hz, 1H), 3.88 (dd, *J* = 18.0, 7.6 Hz, 1H); ^13^C-NMR (DMSO-*d*_6_) δ 197.8, 149.7, 138.0, 136.4, 136.4, 133.3, 132.6, 130.1, 128.6, 128.0, 127.2, 126.2, 123.7, 123.1, 121.2, 118.6, 118.4, 116.2, 111.5, 44.5, 31.5; MS (EI) *m*/*z* (relative intensity) 370 (M^+^, 36), 353 (47), 251 (100), 231 (59), 204 (65), 105 (87); HRMS (EI) *m*/*z* calcd for C_23_H_18_N_2_O_3_ (M^+^) 370.1317, found 370.1310.

*3-(5-Fluoro-2-nitrophenyl)-3-(1H-indol-3-yl)-1-phenylpropan-1-one* (**3b**): Purified by column chromatography using 1:5 ethyl acetate and hexane. After concentration *in vacuo* it give a brown oil; IR (KBr): 3340, 3056, 1670, 1601, 1520, 1474, 1347, 1287, 974, 623 cm^−1^. ^1^H-NMR (CDCl_3_) δ 8.16 (s, 1H), 7.97–7.95 (m, 2H), 7.90 (dd, *J* = 9.0, 5.2 Hz, 1H), 7.56 (t, *J* = 7.4 Hz, 1H), 7.47–7.43 (m, 3H), 7.32 (d, *J* = 8.2 Hz, 1H), 7.16 (dt, *J* = 7.6, 0.7 Hz, 1H), 7.12–7.09 (m, 2H), 7.03 (dt, *J* = 7.1, 0.4 Hz, 1H), 6.95 (ddd, *J* = 9.2, 6.5, 2.7 Hz, 1H), 5.79 (t, *J* = 7.1 Hz, 1H), 3.86 (dd, *J* = 17.3, 7.4 Hz, 1H), 3.76 (dd, *J* = 17.3, 7.4 Hz, 1H); ^13^C-NMR (CDCl_3_) δ 197.4, 164.7 (d, *J_C–F_* = 254 Hz), 146.1 (d, *J_C–F_* = 3 Hz), 143.2, 143.1, 136.7 (d, *J_C–F_* = 10 Hz), 133.6, 128.9, 128.3, 127.5 (d, *J_C–F_* = 10 Hz), 126.5, 122.8, 122.2, 120.1, 119.4, 117.0 (d, *J_C–F_* = 24 Hz), 116.9, 114.6 (d, *J_C–F_* = 23 Hz), 111.5, 44.9, 33.2; HRMS (EI) *m*/*z* calcd for C_23_H_18_N_2_O_3_F ([M + H]^+^) 389.1301, found 389.1318.

*3-(5-Chloro-2-nitrophenyl)-3-(1H-indol-3-yl)-1-phenylpropan-1-one* (**3c**): Purified by column chromatography using 1:4 ethyl acetate and hexane. After concentration *in vacuo* a pale brown solid with a melting point of 151–153 °C was obtained; ^1^H-NMR (CDCl_3_) δ 8.09 (s, 1H), 7.95 (d, *J* = 8.0 Hz, 2H), 7.78 (d, *J* = 8.7, 1H), 7.55 (t, *J* = 7.4 Hz, 1H), 7.46–7.42 (m, 3H), 7.38 (d, *J* = 2.0, 1H), 7.33 (d, *J* = 8.1, 1H), 7.25 (s, 1H), 7.16 (t, *J* = 7.4, 1H), 7.12 (s, 1H), 7.03 (t, *J* = 7.6, 1H), 5.73 (t, *J* = 7.2, 1H), 3.87 (dd, *J* = 17.4, 6.8 Hz, 1H), 3.75 (dd, *J* = 17.2, 7.7 Hz, 1H); ^13^C-NMR (CDCl_3_) δ 197.3, 148.4, 141.4, 139.1, 136.8, 136.7, 133.6, 130.2, 128.9, 128.3, 127.6, 126.5, 126.1, 122.8, 122.2, 120.1, 119.4, 116.8, 111.5, 44.9, 32.9; MS (EI) *m*/*z* (relative intensity) 406 ([M + 2]^+^, 8), 404 (M^+^ , 25), 387 (29), 285 (85), 265 (41), 253 (33), 204 (26), 203 (11), 132 (11), 105 (100); HRMS (EI) *m*/*z* calcd. for C_23_H_17_N_2_O_3_Cl (M^+^) 404.0928, found 404.0926.

*3-(5-Bromo-2-nitrophenyl)-3-(1H-indol-3-yl)-1-phenylpropan-1-one* (**3d**): Purified by column chromatography using 1:4 ethyl acetate and hexane. Concentration *in vacuo* give a pale brown solid with a melting point of 166–168 °C; ^1^H-NMR (CDCl_3_) δ 8.14 (s, 1H), 7.93 (d, *J* = 7.4 Hz, 2H), 7.66 (d, *J* = 8.6, 1H), 7.55–7.52 (m, 2H), 7.46–7.40 (m, 3H), 7.37 (dd, *J* = 8.6, 2.0 Hz, 1H), 7.29 (d, *J* = 8.2 Hz, 1H), 7.14 (t, *J* = 7.3 Hz, 1H), 7.07 (d, *J* = 1.9 Hz, 1H), 7.01 (t, *J* = 7.5 Hz, 1H), 5.70 (t, *J* = 7.1 Hz, 1H), 3.85 (dd, *J* = 17.3, 6.8 Hz, 1H), 3.72 (dd, *J* = 17.2, 7.6 Hz, 1H); ^13^C-NMR (CDCl_3_) δ 197.3, 149.0, 141.4, 136.8, 136.7, 133.6, 133.2, 130.6, 128.9, 128.3, 127.5, 126.5, 126.1, 122.8, 122.2, 120.1, 119.4, 116.8, 111.5, 44.9, 32.8; MS (EI) *m*/*z* (relative intensity) 450 ([M + 2]^+^, 23), 448 (M^+^, 23), 431 (26), 329 (100), 311 (63), 285 (30), 217 (30), 206 (54), 176 (16), 132 (27), 105 (95); HRMS (EI) *m*/*z* calcd. for C_23_H_17_N_2_O_3_Br (M^+^) 448.0423, found 448.0432.

*1-(2-Chlorophenyl)-3-(1H-indol-3-yl)-3-(2-nitrophenyl)propan-1-one* (**3e**): Yellow crystalline solid (crystallized from ethyl acetate and hexane) with a melting point of 114–116 °C; ^1^H-NMR (CDCl_3_) δ 8.07 (s, 1H), 7.80 (d, *J* = 8.1 Hz, 1H), 7.43–7.21 (m, 9H), 7.14 (m, 2H), 6.98 (t, *J* = 7.5 Hz, 1H), 5.55 (t, *J* = 7.4 Hz, 1H), 3.84 (dd, *J* = 17.0, 7.0 Hz, 1H), 3.77 (dd, *J* = 17.0, 8.1 Hz, 1H); ^13^C-NMR (CDCl_3_) δ 201.3, 149.8, 138.9, 138.4, 136.7, 132.8, 131.9, 130.8, 130.6, 130.2, 129.0, 127.4, 127.1, 126.5, 124.5, 122.6, 122.5, 119.8, 119.3, 116.7, 111.4, 49.3, 33.3; MS (EI) *m*/*z* (relative intensity) 406 ([M + 2]^+^, 6), 404 (M^+^ , 18), 354 (24), 269 (44), 251 (74), 207 (46), 204 (42), 139 (100), 105 (76); HRMS (EI) *m*/*z* calcd for C_23_H_17_N_2_O_3_Cl (M^+^) 404.0928, found 404.0929.

*1-(2-Bromophenyl)-3-(1H-indol-3-yl)-3-(2-nitrophenyl)propan-1-one* (**3f**): Purified by column chromatography using 1:5 ethyl acetate and hexane. After concentration *in vacuo* a yellow crystalline solid (from ethyl acetate and hexane) with a melting point of 132–134 °C was obtained; ^1^H-NMR (CDCl_3_) δ 8.08 (s, 1H), 7.81 (d, *J* = 8.1 Hz, 1H), 7.54 (d, *J* = 7.6 Hz, 1H), 7.44–7.38 (m, 2H), 7.33–7.30 (m, 3H), 7.26–7.19 (m, 3H), 7.15–7.12 (m, 2H), 6.98 (t, *J* = 7.7 Hz, 1H), 5.54 (t, *J* = 7.4 Hz, 1H), 3.83 (dd, *J* = 16.9, 6.8 Hz, 1H), 3.74 (dd, *J* = 16.9, 8.0 Hz, 1H); ^13^C-NMR (CDCl_3_) δ 202.0, 149.9, 141.1, 138.3, 136.7, 133.8, 132.9, 131.8, 130.3, 128.7, 127.6, 127.5, 126.5, 124.5, 122.7, 122.5, 119.8, 119.3, 118.7, 116.6, 111.4, 49.0, 33.4; MS (EI) *m*/*z* (relative intensity) 450 ([M + 2]^+^, 10), 448 (M^+^, 10), 354 (27), 251 (60), 204 (50), 183 (100), 155 (16), 132 (14), 105 (13); HRMS (EI) *m*/*z* calcd. for C_23_H_17_N_2_O_3_Br (M^+^) 448.0423, found 448.0420.

*3-(1H-Indol-3-yl)-3-(2-nitrophenyl)-1-p-tolylpropan-1-one* (**3g**): Purified by column chromatography using 1:5 ethyl acetate and hexane. After concentration *in vacuo* an orange oil was obtained; ^1^H-NMR (CDCl_3_) δ 8.01 (s, 1H), 7.86 (d, *J* = 7.9 Hz, 2H), 7.79 (d, *J* = 8.0 Hz, 1H), 7.46–7.38 (m, 3H), 7.31 (t, *J* = 8.2 Hz, 2H), 7.24 (d, *J* = 8.0 Hz, 2H), 7.15 (t, *J* = 7.3 Hz, 1H), 7.11 (s, 1H), 7.00 (t, *J* = 7.6 Hz, 1H), 5.68 (t, *J* = 7.2 Hz, 1H), 3.84 (dd, *J* = 16.8, 7.4 Hz, 1H), 3.77 (dd, *J* = 17.2, 7.8 Hz, 1H), 2.39 (s, 3H); ^13^C-NMR (CDCl_3_) δ 197.4, 150.1, 144.3, 139.0, 136.8, 134.4, 132.7, 130.1, 129.5, 128.4, 127.3, 126.6, 124.4, 122.5, 122.3, 119.8, 119.4, 117.4, 111.4, 44.9, 33.2, 21.8; MS (EI) *m*/*z* (relative intensity) 384 (M^+^, 46), 350 (39), 251 (100), 232 (40), 206 (38), 119 (63); HRMS (EI) *m*/*z* calcd for C_24_H_20_N_2_O_3_ (M^+^) 384.1474, found 384.1473.

*3-(1H-Indol-3-yl)-1-(4-methoxyphenyl)-3-(2-nitrophenyl)propan-1-one* (**3h**): Purified by column chromatography using 1:5 ethyl acetate and hexane. After concentration *in vacuo* a yellow solid with a melting point of 207–209 °C was obtained; ^1^H-NMR (400 MHz,DMSO-*d*_6_) δ 10.96 (s, 1H), 7.98 (d, *J* = 8.8 Hz, 2H), 7.81 (d, *J* = 8.0 Hz, 1H), 7.59 (d, *J* = 7.2 Hz, 1H), 7.52 (t, *J* = 7.3 Hz, 1H), 7.39–7.31 (m, 4H), 7.05–7.00 (m, 3H), 6.89 (t, *J* = 7.4 Hz, 1H), 5.41 (t, *J* = 7.2 Hz, 1H), 3.95 (dd, *J* = 17.7, 6.6 Hz, 1H), 3.83 (s, 3H), 3.77 (dd, *J* = 17.7, 8.0 Hz, 1H); ^13^C-NMR (DMSO-*d*_6_) δ 196.1, 163.2, 149.7, 138.8, 136.3, 132.6, 130.4, 130.0, 129.5, 127.1, 126.2, 123.7, 123.0, 121.2, 118.5, 118.4, 116.3, 113.8, 111.4, 55.5, 44.0, 31.6; MS (EI) *m*/*z* (relative intensity) 400 (M^+^, 25), 366 (29), 250 (63), 222 (29), 206 (22), 135 (100); HRMS (EI) *m*/*z* calcd for C_24_H_20_N_2_O_4_ (M^+^) 400.1423, found 400.1428.

*1-(Benzo[d][1,3]dioxol-5-yl)-3-(1H-indol-3-yl)-3-(2-nitrophenyl)propan-1-one* (**3i**): Purified by column chromatography using 1:5 ethyl acetate and hexane. After concentration *in vacuo* a pale yellow solid with a melting point of 150–152 °C was obtained; ^1^H-NMR (CDCl_3_) δ 8.05 (s, 1H), 7.78 (dd, *J* = 8.0, 0.8 Hz, 1H), 7.58 (dd, *J* = 8.2, 1.7 Hz, 1H), 7.44–7.37 (m, 4H), 7.30–7.26 (m, 2H), 7.13 (dt, *J* = 7.2, 0.5 Hz, 1H), 7.08 (d, *J* = 2.0 Hz, 1H), 6.99 (dt, *J* = 7.5, 0.5 Hz, 1H), 6.81 (d, *J* = 8.1 Hz, 1H), 6.01 (s, 2H), 5.65 (t, *J* = 7.2 Hz, 1H), 3.77 (dd, *J* = 16.8, 7.2 Hz, 1H), 3.70 (dd, *J* = 17.0, 7.5 Hz, 1H); ^13^C-NMR (CDCl_3_) δ 195.8, 152.1, 150.2, 148.4, 138.9, 136.8, 132.7, 131.8, 130.2, 127.3, 126.6, 124.6, 124.5, 122.5, 122.3, 119.9, 119.5, 117.4, 111.4, 108.2, 108.1, 102.0, 44.8, 33.4; MS (EI) *m*/*z* (relative intensity) 414 (M^+^, 18), 380 (17), 251 (65), 207 (20), 148 (100); HRMS (EI) *m*/*z* calcd for C_24_H_18_N_2_O_5_ (M^+^) 414.1216, found 414.1209.

*3-(1H-Indol-3-yl)-1-(naphthalen-2-yl)-3-(2-nitrophenyl)propan-1-one* (**3j**): Purified by column chromatography using 1:5 ethyl acetate and hexane. After concentration *in vacuo* a yellow solid with a melting point of 185–187 °C was isolated; ^1^H-NMR (DMSO-*d*_6_) δ 10.98 (s, 1H), 8.79 (s, 1H), 8.12 (d, *J* = 7.8 Hz, 1H), 7.99–7.96 (m, 3H), 7.84 (dd, *J* = 8.1, 1.1 Hz, 1H), 7.67–7.62 (m, 3H), 7.54 (dt, *J* = 7.7, 1.1 Hz, 1H), 7.44 (d, *J* = 2.2 Hz, 1H), 7.42–7.38 (m, 2H), 7.33 (d, *J* = 8.1 Hz, 1H), 7.04 (dt, *J* = 7.1, 0.8 Hz, 1H), 6.90 (dt, *J* = 7.4, 0.6 Hz, 1H), 5.49 (t, *J* = 7.2 Hz, 1H), 4.19 (dd, *J* = 17.9, 6.8 Hz, 1H), 3.99 (dd, *J* = 17.8, 7.7 Hz, 1H); ^13^C-NMR (DMSO-*d*_6_) δ 197.7, 149.7, 138.8, 136.4, 135.1, 133.7, 132.7, 132.2, 130.1, 130.1, 129.6, 128.7, 128.2, 127.6, 127.2, 126.9, 126.2, 123.8, 123.5, 123.1, 121.2, 118.6, 118.4, 116.2, 111.5, 44.5, 31.7; MS (EI) *m*/*z* (relative intensity) 420 (M^+^, 5), 251 (19), 155 (100), 127 (36); HRMS (EI) *m*/*z* calcd for C_27_H_20_N_2_O_3_ (M^+^) 420.1474, found 420.1465.

*3-(1H-Indol-3-yl)-3-(2-nitrophenyl)-1-(thiophen-2-yl)propan-1-one* (**3k**): Purified by column chromatography using 1:5 ethyl acetate and hexane. After concentration *in vacuo* a pale brown solid with a melting point of 146–148 °C was obtained; ^1^H-NMR (CDCl_3_) δ 8.04 (s, 1H), 7.81–7.79 (m, 2H), 7.61 (d, *J* =4.7 Hz, 1H), 7.47–7.40 (m, 2H), 7.36–7.28 (m, 3H), 7.17–7.10 (m, 3H), 7.00 (t, *J* = 7.5 Hz, 1H), 5.67 (t, *J* = 7.4 Hz, 1H), 3.79 (dd, *J* = 14.5, 5.8 Hz, 1H), 3.74 (dd, *J* = 14.6, 5.6 Hz, 1H); ^13^C-NMR (CDCl_3_) δ 190.7, 150.0, 144.1, 138.6, 136.7, 134.1, 132.8, 132.3, 130.3, 128.4, 127.5, 126.6, 124.5, 122.5, 119.8, 119.3, 116.9, 111.5, 45.6, 33.6; MS (EI) *m*/*z* (relative intensity) 251 (22), 204 (25), 111 (100); HRMS (EI) *m*/*z* calcd for C_21_H_26_N_2_O_3_S (M^+^) 376.0882, found 376.0880.

*3-(5-Fluoro-1H-indol-3-yl)-3-(2-nitrophenyl)-1-phenylpropan-1-one* (**3l**): Purified by column chromatography using 1:4 ethyl acetate and hexane. After concentration *in vacuo* a yellow solid with a melting point of 192–194 °C was obtained; ^1^H-NMR (DMSO-*d*_6_) δ 11.10 (s, 1H), 7.99 (d, *J* = 7.7 Hz, 2H), 7.83 (d, *J* = 8.1 Hz, 1H), 7.63–7.61 (m, 2H), 7.56–7.48 (m, 4H), 7.38 (t, *J* = 7.8 Hz, 1H), 7.33 (dd, *J* = 8.8, 4.6 Hz, 1H), 7.15 (dd, *J* = 10.2, 2.0 Hz, 1H), 6.88 (dt, *J* = 9.2, 2.2 Hz, 1H), 5.37 (t, *J* = 7.3 Hz, 1H), 4.00 (dd, *J* = 18.1, 6.2 Hz, 1H), 3.92 (dd, *J* = 18.1, 8.1 Hz, 1H); ^13^C-NMR (DMSO-*d*_6_) δ 197.8, 156.7 (d, *J_C–F_* = 230 Hz), 149.8, 138.5, 136.4, 133.3, 133.1, 132.7, 129.9, 128.6, 128.0, 127.2, 126.4 (d, *J_C–F_* = 10 Hz), 125.3, 123.8, 116.6 (d, *J_C–F_* = 5 Hz), 112.5 (d, *J_C–F_* = 10 Hz), 109.4 (d, *J_C–F_* = 26 Hz), 103.2 (d, *J_C–F_* = 23 Hz); MS (EI) *m*/*z* (relative intensity) 388 (M^+^, 29), 371 (25), 269 (92), 222 (56); HRMS (EI) *m*/*z* calcd for C_23_H_17_N_2_O_3_F (M^+^) 388.1223, found 388.1227.

*3-(5-Chloro-1H-indol-3-yl)-3-(2-nitrophenyl)-1-phenylpropan-1-one* (**3m**): Purified by column chromatography using 1:4 ethyl acetate and hexane. After concentration *in vacuo* a pale yellow solid with a melting point of 198–200 °C was obtained; ^1^H-NMR (DMSO-*d*_6_) δ 11.22 (s, 1H), 7.99 (d, *J* = 7.4 Hz, 2H), 7.84 (d, *J* = 8.0 Hz, 1H), 7.64–7.61 (m, 2H), 7.56–7.48 (m, 4H), 7.46 (d, *J* = 1.5 Hz, 1H), 7.40–7.34 (m, 2H), 7.04 (dd, *J* = 8.6, 1.8 Hz, 1H), 5.38 (t, *J* = 7.2 Hz, 1H), 4.00 (dd, *J* = 18.2, 6.4 Hz, 1H), 3.92 (dd, *J* = 18.2, 8.4 Hz, 1H); ^13^C-NMR (DMSO-*d*_6_) δ 197.7, 149.8, 138.5, 136.4, 134.8, 133.3, 132.8, 130.0, 128.6, 127.3, 127.3, 125.1, 123.9, 123.3, 121.2, 117.7, 116.2, 113.1, 44.5, 31.3; MS (EI) *m*/*z* (relative intensity) 406 ([M + 2]^+^, 10), 404 (M^+^, 31), 387 (25), 285 (92), 265 (30), 253 (29), 205 (32), 204 (19), 105 (94), 84 (100); HRMS (EI) *m*/*z* calcd for C_23_H_17_N_2_O_3_Cl (M^+^) 404.0928, found 404.0930.

*3-(5-Bromo-1H-indol-3-yl)-3-(2-nitrophenyl)-1-phenylpropan-1-one* (**3n**): Purified by column chromatography using 1:5 ethyl acetate and hexane. Concentration *in vacuo* gave a brown solid with a melting point of 152–154 °C; ^1^H-NMR (DMSO-*d*_6_) δ 11.25 (s, 1H), 8.00 (d, *J* = 7.9 Hz, 2H), 7.84 (d, *J* = 8.1 Hz, 1H), 7.63–7.60 (m, 3H), 7.56–7.52 (m, 2H), 7.50 (t, *J* = 7.6 Hz, 2H), 7.38 (t, *J* = 8.0 Hz, 1H), 7.32 (d, *J* = 8.6 Hz, 1H), 7.16 (d, *J* = 8.6 Hz, 1H), 5.40 (t, *J* = 7.2, 1H), 4.01 (dd, *J* = 18.2, 6.0 Hz, 1H), 3.92 (dd, *J* = 18.2, 8.4 Hz, 1H); ^13^C-NMR (DMSO-*d*_6_) δ 197.7, 149.7, 138.4, 136.4, 135.0, 133.3, 132.8, 129.9, 128.6, 128.0, 128.0, 127.3, 124.9, 123.8, 123.7, 120.7, 116.1, 113.5, 111.2, 44.5, 31.2; MS (EI) *m*/*z* (relative intensity) 450 ([M + 2]^+^, 7), 448 (M^+^, 7), 431 (7), 329 (26), 311 (23), 284 (16), 217 (13), 205 (23), 105 (100); HRMS (EI) *m*/*z* calcd for C_23_H_17_N_2_O_3_Br (M^+^) 448.0423, found 448.0414.

*3-(5-Methoxy-1H-indol-3-yl)-3-(2-nitrophenyl)-1-phenylpropan-1-one* (**3o**): Purified by column chromatography using 1:5 ethyl acetate and hexane. After concentration *in vacuo* a green oil was obtained; ^1^H-NMR (CDCl_3_) δ 8.17 (s, 1H), 7.92 (s, 1H), 7.89 (d, *J* = 1.3 Hz, 1H), 7.71 (dd, *J* = 8.0, 1.1 Hz, 1H), 7.49 (t, *J* = 7.5 Hz, 1H), 7.37 (m, 3H), 7.31 (dt, *J* = 7.7, 1.0 Hz, 1H), 7.19 (dt, *J* = 7.5, 1.2 Hz, 1H), 7.08 (d, *J* = 8.8 Hz, 1H), 6.98 (d, *J* = 2.1 Hz, 1H), 6.88 (d, *J* = 2.4 Hz, 1H), 6.73 (dd, *J* = 8.8, 2.4 Hz, 1H), 5.61 (t, *J* = 7.2 Hz, 1H), 3.79 (dd, *J* = 17.2, 6.9 Hz, 1H), 3.72 (dd, *J* = 17.2, 7.5 Hz, 1H), 3.66 (s, 3H); ^13^C-NMR (CDCl_3_) δ 197.7, 154.3, 150.4, 138.8, 136.8, 133.5, 132.7, 131.8, 130.2, 128.9, 128.3, 127.4, 127.1, 124.4, 122.7, 117.4, 112.9, 112.1, 101.2, 55.9, 45.0, 33.0, MS (EI) *m*/*z* (relative intensity) 400 (M^+^ , 32), 383 (25), 281 (68), 261 (39), 249 (24), 204 (16), 162 (12), 105 (100); HRMS (EI) *m*/*z* calcd for C_24_H_20_N_2_O_4_ (M^+^) 400.1423, found 400.1431.

*3-(7-Methyl-1H-indol-3-yl)-3-(2-nitrophenyl)-1-phenylpropan-1-one* (**3p**): Purified by column chromatography using 1:5 ethyl acetate and hexane. After concentration *in vacuo* a pale green solid with a melting point of 139–141 °C was obtained; ^1^H-NMR (CDCl_3_) δ 7.97–7.95 (m, 3H), 7.80 (d, *J* = 8.1 Hz, 1H), 7.55 (t, *J* = 7.36 Hz, 1H), 7.46–7.39 (m, 4H), 7.31–7.23 (m, 2H), 7.11 (d, *J* = 1.8 Hz, 1H), 6.96–6.91 (m, 2H), 5.67 (t, *J* = 7.2 Hz, 1H), 3.87 (dd, *J* = 16.7, 7.2 Hz, 1H), 3.80 (dd, *J* = 16.7, 6.6 Hz, 1H), 2.45 (s, 3H); ^13^C-NMR (CDCl_3_) δ 197.8, 150.1, 138.9, 136.8, 136.3, 133.4, 132.8, 130.1, 128.8, 128.3, 127.3, 126.1, 124.5, 123.1, 122.0, 120.6, 120.1, 117.8, 117.2, 45.0, 33.3, 16.7; MS (EI) *m*/*z* (relative intensity) 384 (M^+^, 23), 367 (28), 265 (60), 245 (41), 219 (32), 204 (12), 146 (10), 105 (100); HRMS (EI) *m*/*z* calcd for C_24_H_20_N_2_O_3_ (M^+^) 384.1474, found 384.1476.

*3-(1-Methyl-1H-indol-3-yl)-3-(2-nitrophenyl)-1-phenylpropan-1-one* (**3q**): Purified by column chromatography using 1:5 ethyl acetate and hexane. After concentration *in vacuo* a yellow solid with a melting point of 166–168 °C was obtained; ^1^H-NMR (CDCl_3_) δ 7.96 (d, *J* = 7.7 Hz, 2H), 7.80 (d, *J* = 8.1 Hz, 1H), 7.55 (t, *J* = 7.2 Hz, 1H), 7.47–7.39 (m, 5H), 7.30–7.24 (m, 2H), 7.18 (t, *J* = 7.2 Hz, 1H), 7.00 (t, *J* = 7.3 Hz, 1H), 6.95 (s, 1H), 5.67 (t, *J* = 7.2 Hz, 1H), 3.86 (dd, *J* = 16.9, 7.2 Hz, 1H), 3.80 (dd, *J* = 16.8, 6.8 Hz, 1H), 3.74 (s, 3H); ^13^C-NMR (CDCl_3_) δ 197.6, 150.1, 139.1, 137.5, 136.9, 133.4, 132.7, 130.1, 128.8, 128.3, 127.3, 127.0, 127.0, 124.5, 122.2, 119.6, 119.4, 115.9, 109.5, 45.2, 33.1, 33.0; MS (EI) *m*/*z* (relative intensity) 384 (M^+^, 24), 367 (55), 265 (100), 248 (45), 218 (58), 217 (23), 146 (20), 105 (58); HRMS (EI) *m*/*z* calcd for C_24_H_20_N_2_O_3_ (M^+^) 384.1474, found 384.1478.

*3-(2-Methyl-1H-indol-3-yl)-3-(2-nitrophenyl)-1-phenylpropan-1-one* (**3r**): Purified by column chromatography using 1:5 ethyl acetate and hexane. Concentration *in vacuo* gave a yellow solid with a melting point of 129–131 °C; ^1^H-NMR (CDCl_3_) δ 7.88 (s, 1H), 7.86 (d, *J* = 1.0 Hz, 1H), 7.77 (s, 1H), 7.72 (d, *J* = 7.9 Hz, 1H), 7.68 (d, *J* = 8.0 Hz, 1H), 7.53–7.47 (m, 2H), 7.38 (t, *J* = 7.8 Hz, 2H), 7.31 (t, *J* = 8.0 Hz, 2H), 7.20 (d, *J* = 80 Hz, 1H), 7.04 (t, *J* = 7.2 Hz, 1H), 6.95 (t, *J* = 7.3 Hz, 1H), 5.71 (t, *J* = 7.2 Hz, 1H), 3.95 (dd, *J* = 16.9, 7.8 Hz, 1H), 3.84 (dd, J = 16.9, 6.7 Hz, 1H); ^13^C-NMR (CDCl_3_) δ 198.0, 150.4, 138.6, 137.0, 135.7, 133.3, 133.1, 132.3, 129.3, 128.8, 128.2, 127.6, 127.3, 124.9, 121.1, 119.7, 118.6, 111.5, 110.8, 43.3, 33.1, 12.2; MS (EI) *m*/*z* (relative intensity) 384 (M^+^, 53), 367 (17), 265(100), 247 (92), 218 (60), 217 (46), 146 (67), 105 (55); HRMS (EI) *m*/*z* calcd for C_24_H_20_N_2_O_3_ (M^+^) 384.1474, found 384.1481.

*3-(2-Nitrophenyl)-1-phenyl-3-(2-phenyl-1H-indol-3-yl)propan-1-one* (**3s**): Purified by column chromatography using 1:5 ethyl acetate and hexane. After concentration *in vacuo* a deep brown oil was obtained; IR (KBr): 3390, 3059, 1682, 1601, 1526, 1451, 1351, 741, 698 cm^−1^. ^1^H-NMR (CDCl_3_) δ 8.15 (s,1H), 7.74 (d, *J* = 7.4 Hz, 2H), 7.67 (t, *J* = 7.1 Hz, 2H), 7.60 (d, *J* = 7.9 Hz, 1H), 7.43 (t, *J* = 7.4 Hz, 1H), 7.36–7.20 (m, 10H), 7.11 (t, *J* = 7.2 Hz, 1H), 7.04 (t, *J* = 7.5 Hz, 1H), 5.80 (t, *J* = 7.3 Hz, 1H), 4.05 (dd, *J* = 17.4, 8.5 Hz, 1H), 3.78 (dd, *J* = 17.3, 6.1 Hz, 1H); ^13^C-NMR (CDCl_3_) δ 197.6, 149.2, 139.0, 136.6, 136.5, 136.0, 132.9, 132.5, 132.5, 130.1, 128.6, 128.5, 128.4, 128.1, 127.9, 127.8, 127.2, 124.5, 121.9, 119.9, 119.8, 111.8, 111.7, 44.1, 33.8; HRMS (ESI) *m*/*z* calcd for C_29_H_22_N_2_O_3_Na ([M + Na]^+^) 469.1528, found 469.1519.

*3-(1H-Indol-3-yl)-3-(3-methyl-2-nitrophenyl)-1-phenylpropan-1-one* (**3t**): Purified by column chromatography using 1:5 ethyl acetate and hexane. After concentration *in vacuo* a pale pink solid with a melting point of 191–193 °C was obtained; ^1^H-NMR (CDCl_3_) δ 8.00 (s, 1H), 7.93 (d, *J* = 7.6 Hz, 2H), 7.54 (t, *J* = 7.3 Hz, 1H), 7.45–7.38 (m, 3H), 7.26 (t, *J* = 8.1 Hz, 1H), 7.21–7.20 (m, 2H), 7.14–7.10 (m, 2H), 7.02–6.98 (m, 2H), 5.07 (t, *J* = 6.8 Hz, 1H), 3.81 (dd, *J* = 16.8, 8.5 Hz, 1H), 3.69 (dd, *J* = 16.8, 6.0 Hz, 1H), 2.31 (s, 3H); ^13^C-NMR (CDCl_3_) δ 197.6, 151.5, 136.9, 136.8, 135.9, 133.4, 130.2, 129.7, 129.6, 128.9, 128.4, 127.0, 126.6, 122.6, 122.3, 119.9, 119.5, 117.0, 111.3, 45.1, 34.0, 17.7; MS (EI) *m*/*z* (relative intensity) 384 (M^+^, 22), 367 (25), 265 (23), 247 (60), 221 (42), 204 (18); HRMS (EI) *m*/*z* calcd for C_24_H_20_N_2_O_3_ (M^+^) 384.1474, found 384.1476.

*3-(2,5-Dimethyl-1H-indol-3-yl)-3-(2-nitrophenyl)-1-phenylpropan-1-one* (**3u**): Purified by column chromatography using 1:4 ethyl acetate and hexane. After concentration *in vacuo* a green solid with a melting point of 119–121 °C was obtained; ^1^H-NMR (CDCl_3_) δ 7.88 (dd, *J* = 7.8, 1.3 Hz, 2H), 7.71–7.67 (m, 3H), 7.54–7.48 (m, 2H), 7.40 (t, *J* = 7.9 Hz, 2H), 7.32 (dt, *J* = 8.4, 1.2 Hz, 1H), 7.11–7.09 (m, 2H), 6.87 (d, *J* = 8.1 Hz, 1H), 5.69 (t, *J* = 7.1 Hz, 1H), 3.94 (dd, *J* = 17.0, 7.6 Hz, 1H), 3.84 (dd, *J* = 17.0, 6.8 Hz, 1H), 2.34 (s, 3H), 2.33 (s, 3H); ^13^C-NMR (CDCl_3_) δ 198.0, 150.3, 138.7, 137.1, 133.9, 133.2, 133.2, 132.3, 129.5, 128.7, 128.7, 128.2, 127.9, 127.2, 124.8, 122.6, 118.5, 111.0, 110.5, 43.4, 33.1, 21.9, 12.3; MS (EI) *m*/*z* (relative intensity) 398 (M^+^, 25), 262 (53), 261 (36), 207 (33), 160 (48), 105 (100); HRMS (EI) *m*/*z* calcd for C_25_H_22_N_2_O_3_ (M^+^) 398.1630, found 398.1629.

*3-(2,5-Dimethyl-1H-indol-3-yl)-3-(3-methyl-2-nitrophenyl)-1-phenylpropan-1-one* (**3v**): Purified by column chromatography using 1:4 ethyl acetate and hexane. After concentration *in vacuo* a pale yellow solid with a melting point of 173–175 °C was obtained; ^1^H-NMR (CDCl_3_) δ 77.88–7.86 (m, 2H), 7.63 (s, 1H), 7.55–7.50 (m, 2H), 7.40 (t, *J* = 7.8 Hz, 2H), 7.31 (t, *J* = 7.8 Hz, 1H), 7.20 (s, 1H), 7.13 (d, *J* = 7.6 Hz, 1H), 7.09 (d, *J* = 8.2 Hz, 1H), 6.87 (d, *J* = 8.2 Hz, 1H), 5.18 (dd, *J* = 8.2, 6.2 Hz, 1H), 3.96 (dd, *J* = 16.9, 8.4 Hz, 1H), 3.80 (dd, *J* = 16.9, 6.2 Hz, 1H), 2.35 (s, 3H), 2.32 (s, 3H), 2.23 (s, 3H); ^13^C-NMR (CDCl_3_) δ 197.9, 151.8, 137.1, 135.7, 133.9, 133.2, 133.0, 130.0, 129.9, 129.4, 128.7, 128.5, 128.2, 127.6, 126.2, 122.5, 118.5, 110.7, 110.4 ,43.2, 33.0, 21.9, 17.6, 12.1; MS (EI) *m*/*z* (relative intensity) 412 (M^+^, 25), 275 (39), 247 (16), 221 (50), 160 (37), 105 (100); HRMS (EI) *m*/*z* calcd for C_26_H_24_N_2_O_3_ (M^+^) 412.1787, found 412.1780.

### 3.3. General Procedure for Reductive Cyclization (Synthesis of ***4a***–***4u***)

To a stirred solution of **3a** (1 mmol) in ethanol (10 mL), powdered Fe (6 mmol) and HCl (1 mmol) were added and the reaction mixture was kept stirring at reflux until TLC analysis showed complete consumption of **3a**. Then, the reaction mixture was quenched by saturated aq. NaHCO_3_, filtered by celite and concentrated, then the residue was extracted with ethyl acetate three times (10 mL each time). The combined organic layers were dried over magnesium sulfate, filtered and concentrated. The residue was purified by recrystallization or flash chromatography (EtOAc/hexane) to afford the final product **4a**. (The ^1^H-, ^13^C-NMR spectra of the compounds (**4a**–**4u**) was showed in Supplementary).

*4-(1H-Indol-3-yl)-2-phenylquinoline* (**4a**): Pale yellow crystalline solid (crystallized from ethyl acetate and hexane) with a melting point of 229–231 °C; IR (KBr): 3200, 3050, 1591, 1546, 1498, 1237, 828, 741 cm^−1^. ^1^H-NMR (DMSO-*d*_6_) δ 11.78 (s, 1H), 8.31 (d, *J* = 7.2 Hz, 2H), 8.17–8.13 (m, 3H), 7.90 (d, *J* = 1.8 Hz, 1H), 7.79 (t, *J* = 7.3 Hz, 1H), 7.59–7.48 (m, 6H), 7.23 (t, *J* = 7.3 Hz, 1H), 7.11 (t, *J* = 7.5 Hz, 1H); ^13^C-NMR (DMSO-*d*_6_) δ 155.9, 148.5, 142.9, 139.0, 136.6, 129.7, 129.6, 129.4, 128.8, 127.3, 126.6, 126.2, 126.1, 126.0, 125.7, 121.9, 120.0, 119.1, 118.5, 112.2, 112.0; MS (EI) *m*/*z* (relative intensity) 320 (M^+^, 100); HRMS (EI) *m*/*z* calcd for C_23_H_16_N_2_ (M^+^) 320.1313, found 320.1307.

*6-Fluoro-4-(1H-indol-3-yl)-2-phenylquinoline* (**4b**): Purified by column chromatography using 1:4 ethyl acetate and hexane. After concentration *in vacuo* a pale brown solid with a melting point of 154–156 °C was obtained; IR (KBr): 3320, 3048, 1588, 1545, 1494,1240, 830, 750 cm^−1^. ^1^H-NMR (DMSO-*d*_6_) δ 11.81 (s, 1H), 8.31–8.28 (m, 2H), 8.21 (dd, *J* = 17.4, 5.7 Hz, 1H), 8.16 (s, 1H), 7.94 (d, *J* = 2.4 Hz, 1H), 7.79 (dd, *J* = 10.5, 2.6 Hz, 1H), 7.70 (dt, *J* = 8.4, 2.7 Hz, 1H), 7.60–7.48 (m, 5H), 7.24 (t, *J* = 7.6 Hz, 1H), 7.13 (t, *J* = 7.8 Hz, 1H); ^13^C-NMR (DMSO-*d*_6_) δ 159.3 (d, *J_C–F_* = 244 Hz), 155.5 (d, *J_C–F_* = 3 Hz), 145.7, 142.5 (d, *J_C–F_* = 3 Hz), 138.7, 136.6, 132.5 (d, *J_C–F_* = 5 Hz), 129.5, 128.8, 127.2, 126.7, 126.4 (d, *J_C–F_* = 9 Hz), 126.0, 122.0, 120.1, 119.6 (d, *J_C–F_* = 25 Hz), 119.1, 118.9, 112.3, 111.5, 109.3 (d, *J_C–F_* = 23 Hz); MS (EI) *m*/*z* (relative intensity) 338 (M^+^, 100), 337 (65), 285 (24), 149 (87), 105 (30); HRMS (EI) *m*/*z* calcd for C_23_H_15_N_2_F (M^+^) 338.1219, found 338.1213.

*6-Chloro-4-(1H-indol-3-yl)-2-phenylquinoline* (**4c**): Purified by column chromatography using 1:4 ethyl acetate and hexane. After concentration *in vacuo* a pale yellow solid with a melting point of 209–211 °C was obtained; ^1^H-NMR (DMSO-*d*_6_) δ 11.83 (s, 1H), 8.29 (d, *J* = 8.6 Hz, 2H), 8.16–8.13 (m, 2H), 8.10 (d, *J* = 1.8 Hz, 1H), 7.94 (s, 1H), 7.77 (dd, *J* = 8.9, 2.1 Hz, 1H), 7.60–7.48 (m, 5H), 7.24 (t, *J* = 7.3 Hz, 1H), 7.12 (t, *J* = 7.7 Hz, 1H); ^13^C-NMR (DMSO-*d*_6_) δ 156.4, 147.0, 142.4, 138.6, 136.6, 131.8, 130.6, 130.2, 129.7, 128.8, 127.3, 126.9, 126.5, 126.1, 124.8, 122.1, 120.2, 119.4, 118.9, 112.3, 111.4; MS (EI) *m*/*z* (relative intensity) 356 ([M + 2]^+^, 8), 354 (M^+^, 24), 353 (12), 149 (100); HRMS (EI) *m*/*z* calcd for C_23_H_15_N_2_Cl (M^+^) 354.0924, found 354.0919.

*6-Bromo-4-(1H-indol-3-yl)-2-phenylquinoline* (**4d**): Purified by column chromatography using 1:4 ethyl acetate and hexane. After concentration *in vacuo* a yellow solid with a melting point of 214–216 °C was obtained; ^1^H-NMR (DMSO-*d*_6_) δ 11.83 (s, 1H), 8.28 (d, *J* = 7.4 Hz, 2H), 8.26 (d, *J* = 1.8 Hz, 1H), 8.15 (s, 1H), 8.06 (d, *J* = 8.9 Hz, 1H), 7.93 (d, *J* = 2.1 Hz, 1H), 7.86 (dd, *J* = 8.9, 1.8 Hz, 1H), 7.60 (d, *J* = 8.1 Hz, 1H), 7.54–7.46 (m, 4H), 7.24 (t, *J* = 7.3 Hz, 1H), 7.11 (t, *J* = 7.6 Hz, 1H); ^13^C-NMR (DMSO-*d*_6_) δ 156.5, 147.2, 142.3, 138.6, 136.6, 132.7, 131.9, 129.7, 128.8, 128.1, 127.4, 127.0, 126.9, 126.1, 122.1, 120.2, 119.4, 119.2, 118.9, 112.3, 111.4; MS (EI) *m*/*z* (relative intensity) 400 ([M + 2]^+^, 100), 398 (M^+^, 98), 319 (34), 318 (27); HRMS (EI) *m*/*z* calcd for C_23_H_15_N_2_Br (M^+^) 398.0419, found 398.0417.

*2-(2-Chlorophenyl)-4-(1H-indol-3-yl)quinoline* (**4e**): Purified by column chromatography using 1:4 ethyl acetate and hexane. After concentration *in vacuo* a pale yellow solid with a melting point of 242–244 °C was obtained; ^1^H-NMR (DMSO-*d*_6_) δ 11.78 (s, 1H), 8.26 (d, *J* = 8.2 Hz, 1H), 8.13 (d, *J* = 8.2 Hz, 1H), 7.88 (d, *J* = 2.6 Hz, 1H), 7.85- 7.77 (m, 3H), 7.65–7.51 (m, 6H), 7.22 (t, *J* = 7.3 Hz, 1H), 7.12 (t, *J* = 7.5 Hz, 1H); ^13^C-NMR (DMSO-*d*_6_) δ 156.3, 148.4, 141.7, 139.3, 136.6, 131.8, 131.3, 130.2, 129.8, 129.6, 129.6, 127.4, 126.8, 126.6, 126.1, 126.0, 125.4, 122.0, 122.0, 120.1, 118.8, 112.2, 111.4; MS (EI) *m*/*z* (relative intensity) 356 ([M + 2]^+^, 42), 354 (M^+^, 100); HRMS (EI) *m*/*z* calcd for C_23_H_15_N_2_Cl (M^+^) 354.0924, found 354.0924.

*2-(2-Bromophenyl)-4-(1H-indol-3-yl)quinoline* (**4f**): Purified by column chromatography using 1:4 ethyl acetate and hexane). After concentration *in vacuo* a pale yellow solid with a melting point of 236–238 °C was obtained; ^1^H-NMR (DMSO-*d*_6_) δ 11.80 (s, 1H), 8.27 (d, *J* = 7.9 Hz, 1H), 8.13 (d, *J* = 8.1 Hz, 1H), 7.88 (d, *J* = 2.6 Hz, 1H), 7.83–7.78 (m, 3H), 7.73 (dd, *J* = 7.6, 1.6 Hz, 1H), 7.65–7.62 (m, 2H), 7.58–7.52 (m, 2H), 7.41 (dt, *J* = 7.8, 1.8 Hz, 1H), 7.22 (t, *J* = 7.3 Hz, 1H), 7.11 (t, *J* = 7.3 Hz, 1H); ^13^C-NMR (DMSO-*d*_6_) δ 157.8, 148.3, 141.7, 141.3, 136.6, 132.9, 131.7, 130.3, 129.6, 129.6, 127.8, 126.8, 126.6, 126.1, 126.0, 125.4, 122.0, 122.0, 121.1, 120.1, 118.9, 112.2, 111.4; MS (EI) *m*/*z* (relative intensity) 400 ([M + 2]^+^, 100), 399 ([M + 1]^+^, 95), 398 (M^+^, 90), 338 (13), 319 (34), 204 (22), 159 (12); HRMS (EI) *m*/*z* calcd for C_23_H_15_N_2_Br (M^+^) 398.0419, found 398.0412.

*4-(1H-Indol-3-yl)-2-p-tolylquinoline* (**4g**): Purified by column chromatography using 1:4 ethyl acetate and hexane. After concentration *in vacuo* an orange solid with s melting point of 277–279 °C was obtained; IR (KBr): 3300, 3040, 1585, 1439, 1364, 1240, 814, 734 cm^−1^. ^1^H-NMR (DMSO-*d*_6_) δ 11.74 (s, 1H), 8.21 (d, *J* = 8.1 Hz, 2H), 8.13 (d, *J* = 8.7 Hz, 2H), 8.09 (s, 1H), 7.88 (d, *J* = 2.2 Hz, 1H), 7.78 (t, *J* = 6.8 Hz, 1H), 7.57–7.51 (m, 3H), 7.37 (d, *J* = 8.0 Hz, 2H), 7.23 (t, *J* = 7.4 Hz, 1H), 7.11 (t, *J* = 7.4 Hz, 1H), 2.40 (s, 3H); ^13^C-NMR (DMSO-*d*_6_) δ 155.8, 148.5, 142.8, 139.0, 136.5, 136.2, 129.6, 129.6, 129.4, 127.1, 126.5, 126.2, 126.0, 125.9, 125.6, 121.9, 120.0, 119.1, 118.3, 112.2, 112.0, 20.9; MS (EI) *m*/*z* (relative intensity) 334 (M^+^, 100); HRMS (EI) *m*/*z* calcd for C_24_H_18_N_2_ (M^+^) 334.1470, found 334.1469.

*4-(1H-Indol-3-yl)-2-(4-methoxyphenyl)quinoline* (**4h**): Purified by column chromatography using 1:4 ethyl acetate and hexane. After concentration *in vacuo* a pale yellow solid with a melting point of 231–233 °C was obtained; ^1^H-NMR (DMSO-*d*_6_) δ 11.74 (s, 1H), 8.28 (td, *J* = 8.8, 2.8 Hz, 2H), 8.11 (ddd, *J* = 8.6, 2.3, 1.0 Hz, 2H), 8.08 (s, 1H), 7.88 (d, *J* = 2.6 Hz, 1H), 7.77 (ddd, *J* = 8.1, 7.0, 1.1 Hz, 1H), 7.57 (d, *J* = 8.1 Hz, 1H), 7.53–7.50 (m, 2H), 7.23 (ddd, *J* = 7.8, 7.2, 0.6 Hz, 1H), 7.13–7.09 (m, 3H), 3.85 (s, 3H); ^13^C-NMR (DMSO-*d*_6_) δ 160.5, 155.5, 148.5, 142.6, 136.5, 131.4, 129.5, 129.5, 128.6, 126.4, 126.3, 125.9, 125.6, 125.4, 121.8, 119.9, 119.1, 118.1, 114.1, 112.1, 112.1, 55.2; MS (EI) *m*/*z* (relative intensity) 350 (M^+^, 100), 349 (64); HRMS (EI) *m*/*z* calcd for C_24_H_18_N_2_O (M^+^) 350.1419, found 350.1412.

*2-(Benzo[d][1,3]dioxol-5-yl)-4-(1H-indol-3-yl)quinoline* (**4i**): Purified by column chromatography using 1:3 ethyl acetate and hexane. After concentration *in vacuo* a yellow solid with a melting point of 237–239 °C was obtained; ^1^H-NMR (DMSO-*d*_6_) δ 11.74 (s, 1H), 8.10 (d, *J* = 8.4 Hz, 2H), 8.06 (s, 1H), 7.89–7.85 (m, 3H), 7.77 (t, *J* = 7.2 Hz, 1H), 7.57–7.50 (m, 3H), 7.22 (t, *J* = 7.4 Hz, 1H), 7.13–7.07 (m, 2H), 6.12 (s, 2H); ^13^C-NMR (DMSO-*d*_6_) δ 155.3, 148.6, 148.4, 148.1, 142.8, 136.6, 133.4, 129.6, 129.6, 126.6, 126.3, 126.0, 125.8, 125.6, 121.9, 121.7, 120.0, 119.2, 118.3, 112.2, 112.1, 108.5, 107.3, 101.4; MS (EI) *m*/*z* (relative intensity) 364 (M^+^, 100); HRMS (EI) *m*/*z* calcd for C_26_H_14_N_2_O_2_ (M^+^) 364.1212, found 364.1218.

*4-(1H-Indol-3-yl)-2-(naphthalen-2-yl)quinoline* (**4j**): Purified by column chromatography using 1:4 ethyl acetate and hexane. After concentration *in vacuo* an orange solid with a melting point > 300 °C was obtained; ^1^H-NMR (DMSO-*d*_6_) δ 11.77 (s, 1H), 8.87 (s, 1H), 8.54 (d, *J* = 8.5 Hz, 1H), 8.32 (s, 1H), 8.21 (d, *J* = 8.4 Hz, 1H), 8.16 (d, *J* = 8.3 Hz, 1H), 8.13–8.09 (m, 2H), 8.01–7.99 (m, 1H), 7.93 (s, 1H), 7.83 (t, *J* = 7.2 Hz, 1H), 7.59–7.55 (m, 5H), 7.24 (t, *J* = 7.4 Hz, 1H), 7.13 (t, *J* = 7.4 Hz, 1H); ^13^C-NMR (DMSO-*d*_6_) δ 155.7, 148.5, 143.0, 136.6, 136.3, 133.4, 133.1, 129.7, 129.7, 128.8, 128.3, 127.5, 126.9, 126.8, 126.6, 126.5, 126.3, 126.2, 126.1, 125.8, 124.8, 121.9, 120.0, 119.1, 118.8, 112.2, 112.0; HRMS (ESI) *m*/*z* calcd for C_27_H_19_N_2_ ([M + H]^+^) 371.1548, found 371.1554.

*4-(1H-Indol-3-yl)-2-(thiophen-2-yl)quinoline* (**4k**): Purified by column chromatography using 1:4 ethyl acetate and hexane. After concentration *in vacuo* a yellow solid with a melting point of 201–203 °C was obtained; IR (KBr): 3300, 3054, 1587, 1545, 1425, 1370, 1240, 811, 744 cm^−1^. ^1^H-NMR (DMSO-*d*_6_) δ 11.75 (s, 1H), 8.11 (s, 1H), 8.08 (d, *J* = 8.4 Hz, 1H), 8.04 (d, *J* = 8.3 Hz, 1H), 8.01 (d, *J* = 3.7 Hz, 1H), 7.87 (d, *J* = 2.5 Hz, 1H), 7.78–7.73 (m, 2H), 7.57 (d, *J* = 8.2 Hz, 1H), 7.53–7.50 (m, 2H), 7.25–7.20 (m, 2H), 7.11 (t, *J* = 7.8 Hz, 1H); ^13^C-NMR (DMSO-*d*_6_) δ 151.6, 148.2, 145.0, 142.8, 136.5, 129.8, 129.3, 129.0, 128.5, 126.8, 126.6, 126.3, 126.1, 125.8, 125.8, 121.9, 120.0, 119.1, 117.4, 112.2, 111.7; MS (EI) *m*/*z* (relative intensity) 326 (M^+^, 100), 163 (10), 84 (13), 66 (15); HRMS (EI) *m*/*z* calcd for C_21_H_14_N_2_S (M^+^) 326.0878, found 326.0883.

*4-(5-Fluoro-1H-indol-3-yl)-2-phenylquinoline* (**4l**): Pale yellow crystalline solid (crystallized from ethyl acetate and hexane) with a melting point of 236–238 °C; ^1^H-NMR (DMSO-*d*_6_) δ 11.87 (s, 1H), 8.32 (d, *J* = 7.4 Hz, 2H), 8.16 (d, *J* = 8.4 Hz, 1H), 8.11–8.10 (m, 2H), 7.97 (d, *J* = 2.3 Hz, 1H), 7.80 (t, *J* = 7.6 Hz, 1H), 7.84–7.59 (m, 5H), 7.22 (dd, *J* = 9.9, 1.8 Hz, 1H), 7.08 (dt, *J* = 9.1, 2.1 Hz, 1H); ^13^C-NMR (DMSO-*d*_6_) δ 157.6 (d, *J_C–F_* = 232 Hz), 155.9, 128.5, 142.4, 139.0, 133.3, 129.8, 129.7, 129.4, 128.8, 128.6, 127.3, 126.6 (d, *J_C–F_* = 10 Hz), 126.2, 125.9, 125.6, 118.6, 113.3 (d, *J_C–F_* = 10 Hz), 112.3 (d, *J_C–F_* = 4 Hz), 110.3 (d, *J_C–F_* = 26 Hz), 103.9 (d, *J_C–F_* = 23 Hz); MS (EI) *m*/*z* (relative intensity) 338 (M^+^, 100); HRMS (EI) *m*/*z* calcd for C_23_H_15_N_2_F (M^+^) 338.1219, found 338.1210.

*4-(5-Chloro-1H-indol-3-yl)-2-phenylquinoline* (**4m**): Yellow crystalline solid (crystallized from ethyl acetate and hexane) with a melting point of 204–206 °C; ^1^H-NMR (DMSO-*d*_6_) δ 11.97 (s, 1H), 8.32 (d, *J* = 7.4 Hz, 2H), 8.16 (d, *J* = 8.4 Hz, 1H), 8.12 (s, 1H), 8.07 (d, *J* = 8.3 Hz, 1H), 7.98 (s, 1H), 7.80 (t, *J* = 7.7 Hz, 1H), 7.60–7.48 (m, 5H), 7.46 (d *J* = 1.0 Hz, 1H), 7.20 (dd, *J* = 8.6, 1.4 Hz, 1H); ^13^C-NMR (DMSO-*d*_6_) δ 155.9, 148.5, 142.2, 139.0, 135.1, 129.8, 129.7, 129.4, 128.7, 128.2, 127.5, 127.3, 126.2, 125.8, 125.7, 124.7, 122.0, 118.8, 118.3, 113.8, 111.9; MS (EI) *m*/*z* (relative intensity) 356 ([M + 2]^+^, 33), 354 (M^+^, 100); HRMS (EI) *m*/*z* calcd for C_23_H_15_N_2_Cl (M^+^) 354.0924, found 354.0924.

*4-(5-Bromo-1H-indol-3-yl)-2-phenylquinoline* (**4n**): Purified by column chromatography using 1:4 ethyl acetate and hexane). After concentration *in vacuo* a pale green solid with a melting point of 255–257 °C was obtained; ^1^H-NMR (DMSO-*d*_6_) δ 12.00 (s, 1H), 8.32 (d, *J* = 7.3 Hz, 2H), 8.16 (d, *J* = 8.4 Hz, 1H), 8.12 (s, 1H), 8.07 (d, *J* = 8.2 Hz, 1H), 7.97 (s, 1H), 7.78 (t, *J* = 7.5 Hz, 1H), 7.61–7.46 (m, 6H), 7.34 (d, *J* = 8.6 Hz, 1H); ^13^C-NMR (DMSO-*d*_6_) δ 155.9, 148.4, 142.1, 138.9, 135.3, 129.8, 129.7, 129.4, 128.8, 128.1, 128.0, 127.3, 126.2, 125.8, 125.7, 124.5, 121.2, 118.8, 114.2, 112.5, 111.7; MS (EI) *m*/*z* (relative intensity) 400 ([M + 2]^+^, 85), 398 (M^+^, 100); HRMS (EI) *m*/*z* calcd for C_23_H_15_N_2_Br (M^+^) 398.0419, found 398.0425.

*4-(5-Methoxy-1H-indol-3-yl)-2-phenylquinoline* (**4o**): White crystalline solid (crystallized from ethyl acetate and hexane) with a melting point of 183–185 °C; ^1^H-NMR (DMSO-*d*_6_) δ 11.63 (s, 1H), 8.31 (dd, *J* = 7.2 Hz, 2H), 8.15 (d, *J* = 8.7 Hz, 2H), 8.13 (s, 1H), 7.85 (d, *J* = 2.6 Hz, 1H), 7.79 (dt, *J* = 7.0, 1.8 Hz, 1H), 7.59–7.54 (m, 3H), 7.52–7.46 (m, 2H), 6.96 (d, *J* = 2.3 Hz, 1H), 6.88 (dd, *J* = 8.8, 2.4 Hz, 1H), 3.67 (s, 3H); ^13^C-NMR (DMSO-*d*_6_) δ 155.9, 154.1, 148.6, 143.1, 139.0, 131.7, 129.8, 129.7, 129.4, 128.8, 127.3, 127.2, 126.6, 126.2, 126.0 125.7, 118.4, 113.0,112.1, 112.0, 100.9, 55.3; MS (EI) *m*/*z* (relative intensity) 350 (M^+^, 100), 349 (18); HRMS (EI) *m*/*z* calcd for C_24_H_18_N_2_O (M^+^) 350.1419, found 350.1422.

*4-(7-Methyl-1H-indol-3-yl)-2-phenylquinoline* (**4p**): Purified by column chromatography using 1:4 ethyl acetate and hexane. After concentration *in vacuo* a pale yellow solid with a melting point of 163–165 °C was obtained; ^1^H-NMR (DMSO-*d*_6_) δ 11.74 (s, 1H), 8.30 (d, *J* = 7.3 Hz, 2H), 8.15 (d, *J* = 8.7 Hz, 2H), 8.12 (s, 1H), 7.88 (d, *J* = 2.6 Hz, 1H), 7.79 (t, *J* = 7.7 Hz, 1H), 7.58–7.48 (m, 4H), 7.37–7.34 (m, 1H), 7.03–7.00 (m, 2H), 2.58 (s, 3H); ^13^C-NMR (DMSO-*d*_6_) δ 155.8, 148.5, 143.1, 139.0, 136.1, 129.7, 129.6, 129.4, 128.8, 127.3, 126.3, 126.1, 126.0, 126.0, 125.7, 122.4, 121.4, 120.2, 118.6, 116.7, 112.5, 16.8; MS (EI) *m*/*z* (relative intensity) 334 (M^+^, 100), 333 (68); HRMS (EI) *m*/*z* calcd for C_24_H_18_N_2_ (M^+^) 334.1470 found 334.1477.

*4-(1-Methyl-1H-indol-3-yl)-2-phenylquinoline* (**4q**): White crystalline solid (from ethyl acetate and hexane) with a melting point of 138–140 °C; ^1^H-NMR (DMSO-*d*_6_) δ 8.30 (d, *J* = 8.0 Hz, 2H), 8.17 (dd, *J* = 10.9, 8.8 Hz, 2H), 8.11 (s, 1H), 7.91 (s, 1H), 7.80 (dd, *J* = 8.0, 7.1 Hz, 1H), 7.61 (d, *J* = 8.3 Hz, 1H), 7.59–7.49 (m, 5H), 7.30 (t, *J* = 7.4 Hz, 1H), 7.16 (t, *J* = 7.6 Hz, 1H), 3.96 (s, 3H); ^13^C-NMR (DMSO-*d*_6_) δ 155.8, 148.5, 142.4, 139.0, 137.0, 130.7, 129.7, 129.7, 129.4, 128.8, 127.2, 126.5, 126.1, 125.9, 125.6, 122.0, 120.3, 119.3, 118.4, 111.0, 110.5, 32.8; MS (EI) *m*/*z* (relative intensity) 334 (M^+^, 100), 333 (61); HRMS (EI) *m*/*z* calcd for C_24_H_18_N_2_ (M^+^) 334.1470, found 334.1469.

*4-(2-Methyl-1H-indol-3-yl)-2-phenylquinoline* (**4r**): Purified by column chromatography using 1:4 ethyl acetate and hexane. After concentration *in vacuo* a brown solid with a melting point of 98–100 °C was obtained; ^1^H-NMR (DMSO-*d*_6_) δ 8.73 (s, 1H), 8.29 (d, *J* = 8.4 Hz, 1H), 8.16 (d, *J* = 7.6 Hz, 2H), 7.87 (s, 1H), 7.81 (d, *J* = 8.3 Hz, 1H), 7.68 (t, *J* = 7.9 Hz, 1H), 7.47 (t, *J* = 7.3 Hz, 1H), 7.42–7.29 (m, 4H), 7.17 (t, *J* = 7.3 Hz, 1H), 7.07 (t, *J* = 7.4 Hz, 1H), 2.21 (s, 3H); ^13^C-NMR (DMSO-*d*_6_) δ 157.4, 149.1, 143.5, 140.0, 135.6, 133.8, 129.9, 129.8, 129.4, 129.0, 128.7, 127.9, 127.2, 126.8, 126.0, 121.9, 121.2, 120.3, 119.1, 110.9, 110.8, 12.7; MS (EI) *m*/*z* (relative intensity) 334 (M^+^, 100), 333 (55), HRMS (EI) *m*/*z* calcd for C_24_H_18_N_2_ (M^+^) 334.1470, found 334.1464.

*2-Phenyl-4-(2-phenyl-1H-indol-3-yl)quinoline* (**4s**): Purified by column chromatography using 1:4 ethyl acetate and hexane. After concentration *in vacuo* an orange solid with a melting point of 282–284 °C was obtained; ^1^H-NMR (DMSO-*d*_6_) δ 12.00 (s, 1H), 8.21 (d, *J* = 7.3 Hz, 2H), 8.15 (d, *J* = 8.4 Hz, 1H), 8.04 (s, 1H), 7.72–7.65 (m, 2H), 7.60 (d, *J* = 8.1 Hz, 1H), 7.50–7.42 (m, 5H), 7.22 (t, *J* = 7.7 Hz, 1H), 7.26–7.16 (m, 5H), 7.01 (t, *J* = 7.5 Hz, 1H); ^13^C-NMR (DMSO-*d*_6_) δ 155.9, 148.4, 143.6, 138.7, 136.3, 135.9, 131.9, 129.7, 129.7, 129.5, 128.9, 128.8, 128.6, 127.7, 127.5, 127.2, 126.4, 126.1, 126.0, 122.4, 120.6, 120.1, 118.7, 111.7, 109.1; MS (EI) *m*/*z* (relative intensity) 396 (M^+^, 100), 395 (36), 193 (14); HRMS (EI) *m*/*z* calcd for C_29_H_20_N_2_ (M^+^) 396.1626, found 396.1634.

*4-(1H-Indol-3-yl)-8-methyl-2-phenylquinoline* (**4t**): Purified by column chromatography using 1:4 ethyl acetate and hexane. After concentration *in vacuo* a pale orange solid with a melting point of 102–104 °C was obtained; ^1^H-NMR (CDCl_3_) δ 11.75 (s, 1H), 8.35 (d, *J* = 7.5 Hz, 2H), 8.13 (s, 1H), 7.99 (d, *J* = 8.3 Hz, 1H), 7.86 (d, *J* = 2.3 Hz, 1H), 7.63–7.46 (m, 6H), 7.40 (t, *J* = 8.1 Hz, 1H), 7.22 (t, *J* = 7.2 Hz, 1H), 7.10 (t, *J* = 7.4 Hz, 1H), 2.87 (s, 3H); ^13^C-NMR (CDCl_3_) δ 154.4, 147.3, 143.2, 139.3, 137.0, 136.6, 129.6, 129.3, 128.8, 127.2, 126.5, 126.4, 125.7, 124.0, 121.9, 120.0, 119.1, 118.3, 112.4, 112.2, 18.2; MS (EI) *m*/*z* (relative intensity) 334 (M^+^, 100), 333 (30), 219 (30); HRMS (EI) *m*/*z* calcd for C_24_H_18_N_2_ (M^+^) 334.1470, found 334.1471.

*4-(2,5-Dimethyl-1H-indol-3-yl)-2-phenylquinoline* (**4u**): Purified by column chromatography using 1:4 ethyl acetate and hexane. After concentration *in vacuo* an orange solid with a melting point of 181–183 °C was obtained; ^1^H-NMR (CDCl_3_) δ 8.27 (d, *J* = 8.4 Hz, 1H), 8.20 (d, *J* = 7.4 Hz, 3H), 7.90 (s, 1H), 7.84 (d, *J* = 8.3 Hz, 1H), 7.73 (ddd, *J* = 8.1, 7.1, 1.0 Hz, 1H), 7.54 (t, *J* = 7.2 Hz, 2H), 7.49–7.41 (m, 2H), 7.31 (d, *J* = 8.2 Hz, 1H), 7.12 (s, 1H), 7.05 (d, *J* = 8.2 Hz, 1H), 2.38 (s, 3H), 2.37 (s, 3H); ^13^C-NMR (CDCl_3_) δ 157.3, 149.2, 143.4, 140.3, 133.9, 133.6, 130.3, 129.9, 129.7, 129.4, 129.1, 129.0, 127.9, 127.3, 126.8, 126.1, 123.7, 121.2, 119.0, 110.9, 110.4, 21.7, 13.0; MS (EI) *m*/*z* (relative intensity) 348 (M^+^, 100), 347 (35), 166 (11); HRMS (EI) *m*/*z* calcd for C_25_H_20_N_2_ (M^+^) 348.1626, found 348.1632.

*2-Phenylquinoline* (**5a**): Purified by column chromatography using 1:6 ethyl acetate and hexane. After concentration *in vacuo* a white solid with a melting point of 84–86 °C was obtained; ^1^H-NMR (CDCl_3_) δ 8.46 (d, *J* = 8.7 Hz, 1H), 8.28 (d, *J* = 7.1 Hz, 2H), 8.15 (d, *J* = 8.7 Hz, 1H), 8.08 (d, *J* = 7.8 Hz, 1H), 8.00 (d, *J* = 7.8 Hz, 1H), 7.79 (ddd, *J* = 8.2, 7.0, 1.2 Hz, 1H), 7.62–7.45 (m, 4H); ^13^C-NMR (CDCl_3_) δ 156.1, 147.6, 138.7, 137.2, 130.0, 129.6, 129.1, 128.9, 127.8, 127.2, 127.0, 126.5, 118.8; MS (EI) *m*/*z* (relative intensity) 205 (M^+^, 100), 204 (78); HRMS (EI) *m*/*z* calcd for C_15_H_11_N (M^+^) 205.0891, found 205.0889.

*8-Methyl-2-phenylquinoline* (**5v**): Purified by column chromatography using 1:6 ethyl acetate and hexane. After concentration *in vacuo* an orange oil was obtained; ^1^H-NMR (CDCl_3_) δ 8.30 (d, *J* = 7.5 Hz, 2H), 8.18 (d, *J* = 8.5 Hz, 1H), 7.91 (d, *J* = 8.5 Hz, 1H), 7.67 (d, *J* = 8.0 Hz, 1H), 7.61–7.54 (m, 3H), 7.49 (t, *J* = 7.1 Hz, 1H), 7.43 (t, *J* = 7.5 Hz, 1H), 2.94 (s, 3H); ^13^C-NMR (CDCl_3_) δ 155.7, 147.4, 140.1, 137.9, 137.1, 129.9, 129.4, 129.0, 127.7, 127.3, 126.2, 125.6, 118.4, 18.1; MS (EI) *m*/*z* (relative intensity) 219 (M^+^, 100); HRMS (EI) *m*/*z* calcd for C_16_H_13_N (M^+^) 219.1048, found 219.1044.

## 4. Conclusions

In summary, we have successfully developed a strategy for the synthesis of 4-indolylquinoline derivatives from 2-nitrochalcone derivatives in two steps. The process involves as a first step the Michael addition of indole to nitrochalcones under solvent free conditions catalyzed by sulfamic acid and the second step is a reductive cyclization of the 3-(1*H*-indol-3-yl)-3-(2-nitrophenyl)-1-phenylpropan-1-one derivatives to 4-indolylquinoline derivatives via reductive cyclization by Fe/HCl in ethanol. A wide substrate scope, clean reactions and high yields of the products are the main merits of this strategy. This procedure offers an easy, convenient and alternative method to existing methodologies for the synthesis of indolylquinoline derivatives.

## Supplementary Materials

Supplementary materials can be accessed at: http://www.mdpi.com/1420-3049/20/12/19862/s1.
