# Supplementary material for: Syntheses of 4-Indolylquinoline Derivatives via Reductive Cyclization of Indolylnitrochalcone Derivatives by Fe/HCl
Source: Molecules. 2015 Dec 15;20(12):22499–519. doi: 10.3390/molecules201219862 (PMC6332431; doi:10.3390/molecules201219862)
Supplement: Supplementary file 1 [file molecules-20-19862-s001.pdf]

Supplementary Materials: Syntheses of 4-Indolylquinoline Derivatives via Reductive Cyclization of Indolylnitrochalcone Derivatives by Fe/HCl

Wen-Chang Chen, Chan-Chieh Lin, Veerababurao Kavala, Chun-Wei Kuo, Chia-Yu Huang and Ching-Fa Yao

^{1}H-NMR and ^{13}C-NMR Spectra copies

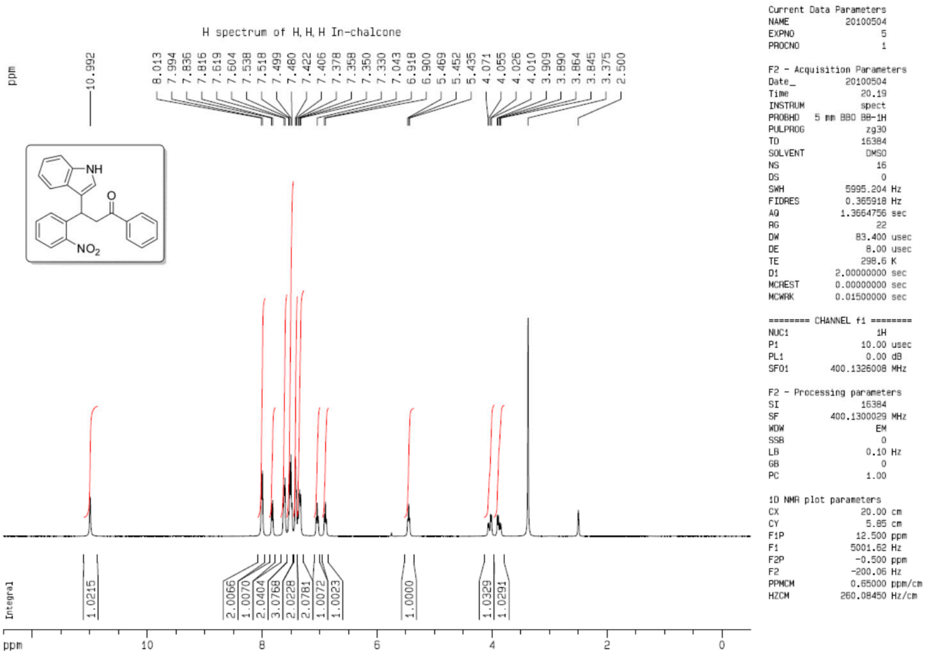

Figure S1. ^{1}H-NMR of 3-(1*H*-indol-3-yl)-3-(2-nitrophenyl)-1-phenylpropan-1-one (**3a**).

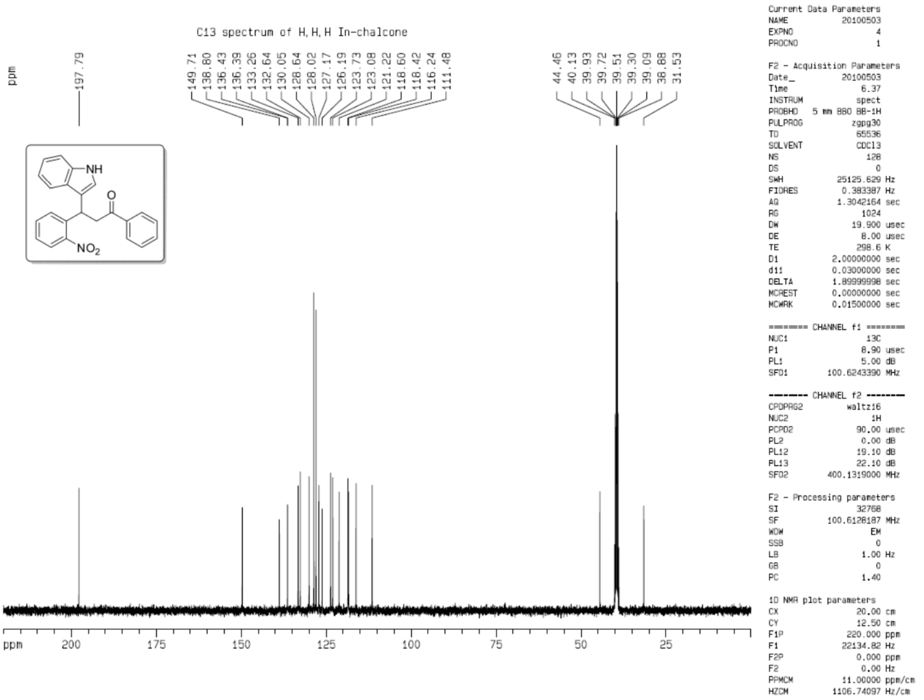

Figure S2. ^{13}C-NMR of 3-(1*H*-indol-3-yl)-3-(2-nitrophenyl)-1-phenylpropan-1-one (**3a**).

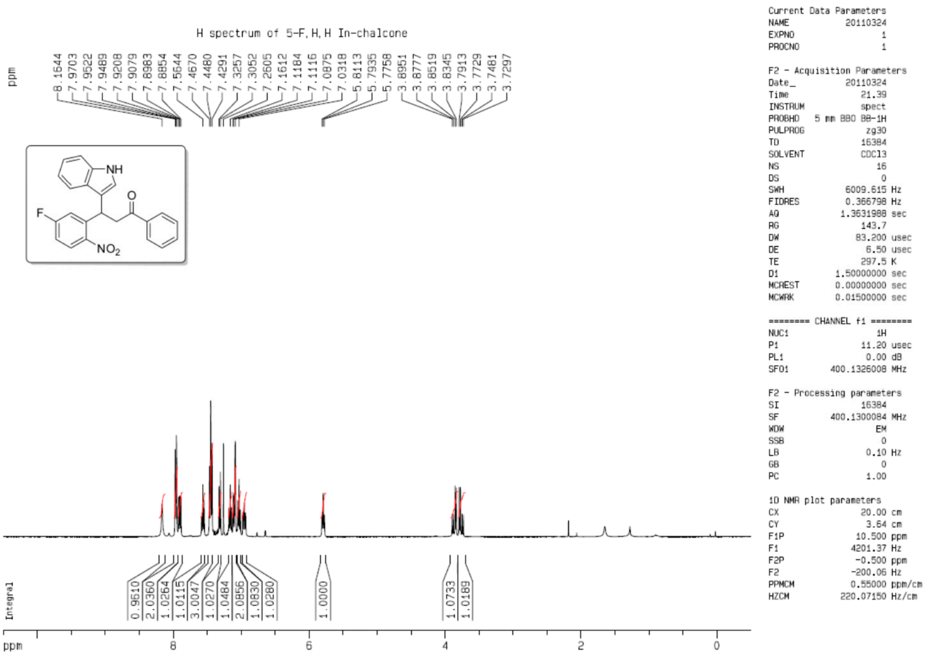

Figure S3. 3-(5-fluoro-2-nitrophenyl)-3-(1*H*-indol-3-yl)-1-phenylpropan-1-one (**3b**).

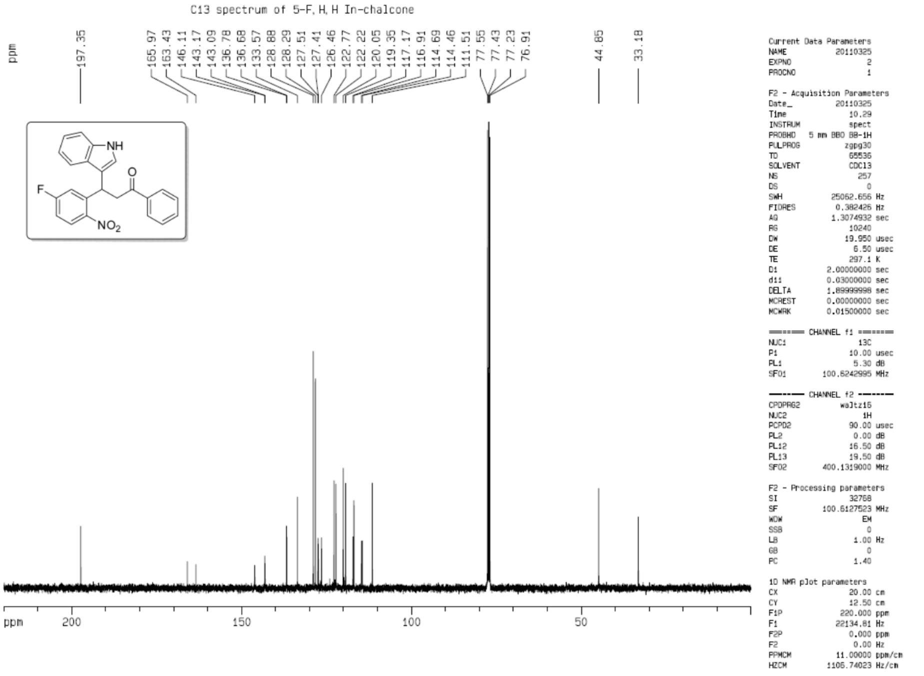

Figure S4. 3-(5-fluoro-2-nitrophenyl)-3-(1*H*-indol-3-yl)-1-phenylpropan-1-one (**3b**).

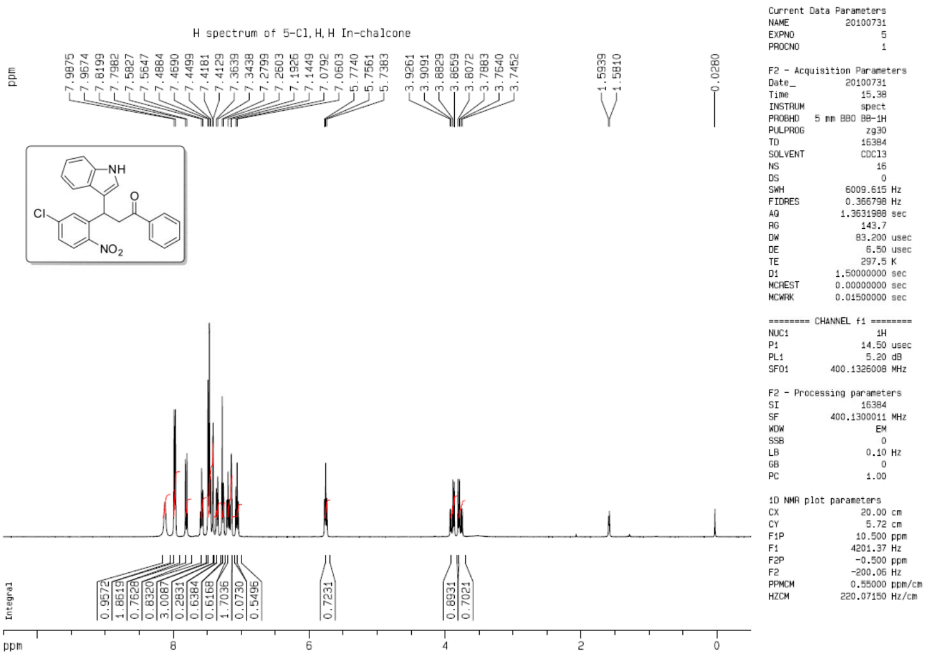

Figure S5. 3-(5-chloro-2-nitrophenyl)-3-(1*H*-indol-3-yl)-1-phenylpropan-1-one (**3c**).

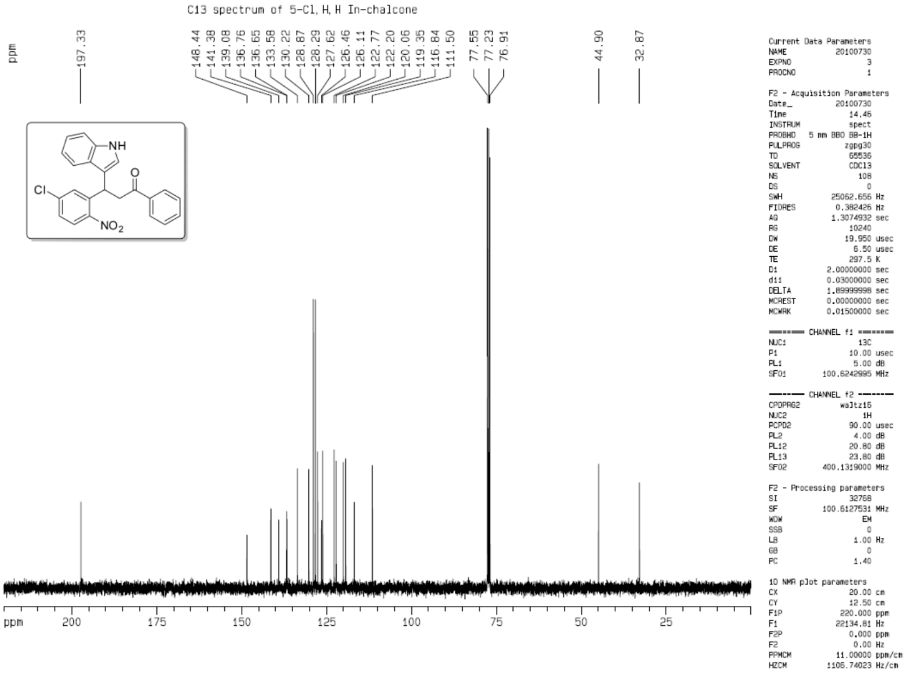

Figure S6. 3-(5-chloro-2-nitrophenyl)-3-(1*H*-indol-3-yl)-1-phenylpropan-1-one (**3c**).

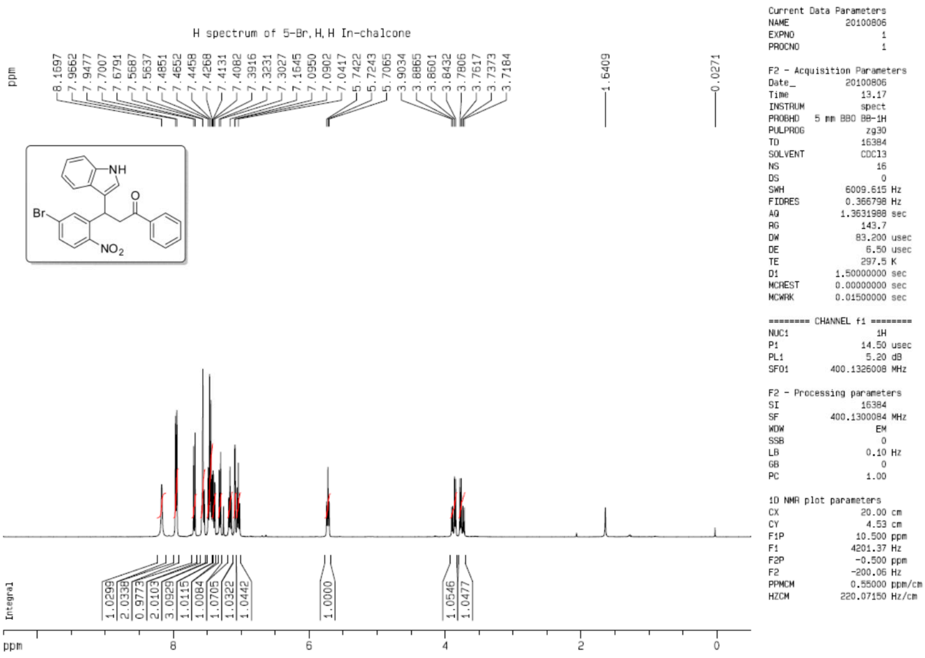

Figure S7. ^{1}H-NMR 3-(5-bromo-2-nitrophenyl)-3-(1*H*-indol-3-yl)-1-phenylpropan-1-one (**3d**).

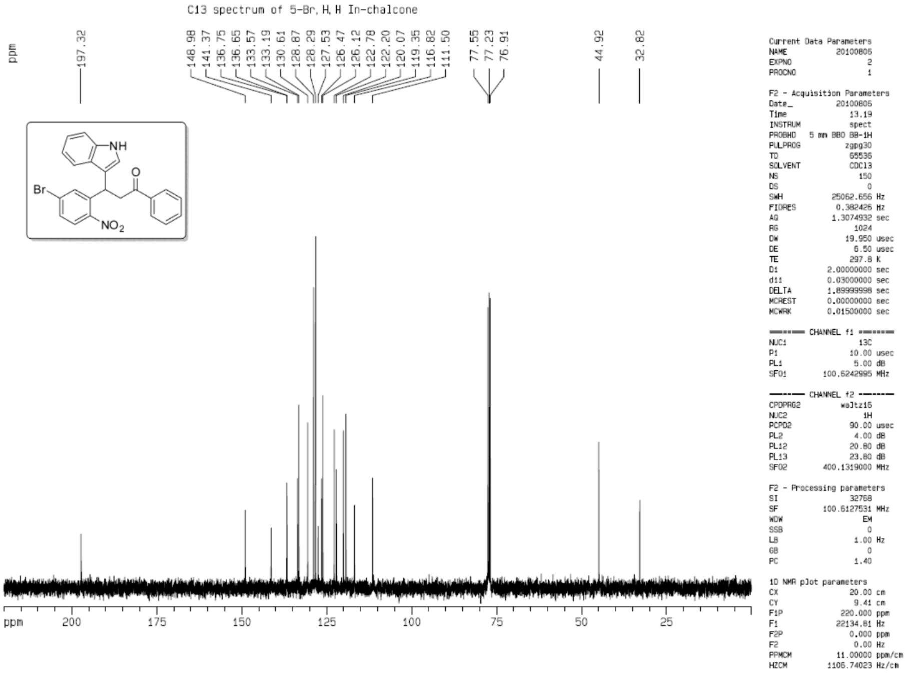

Figure S8. ^{1}H-NMR 3-(5-bromo-2-nitrophenyl)-3-(1*H*-indol-3-yl)-1-phenylpropan-1-one (**3d**).

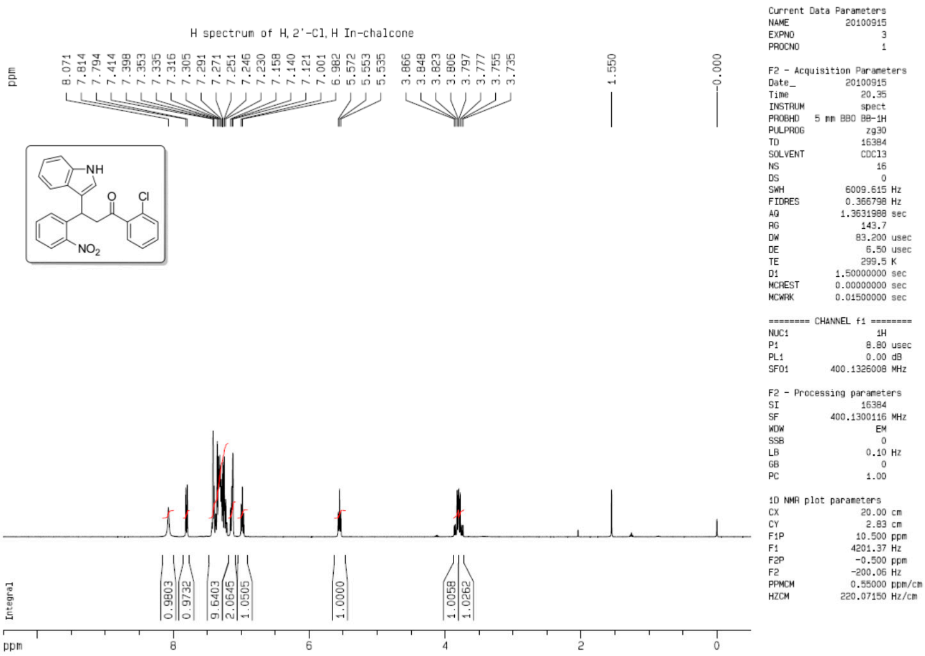

Figure S9. ^{1}H-NMR1-(2-chlorophenyl)-3-(1*H*-indol-3-yl)-3-(2-nitrophenyl)propan-1-one (**3e**).

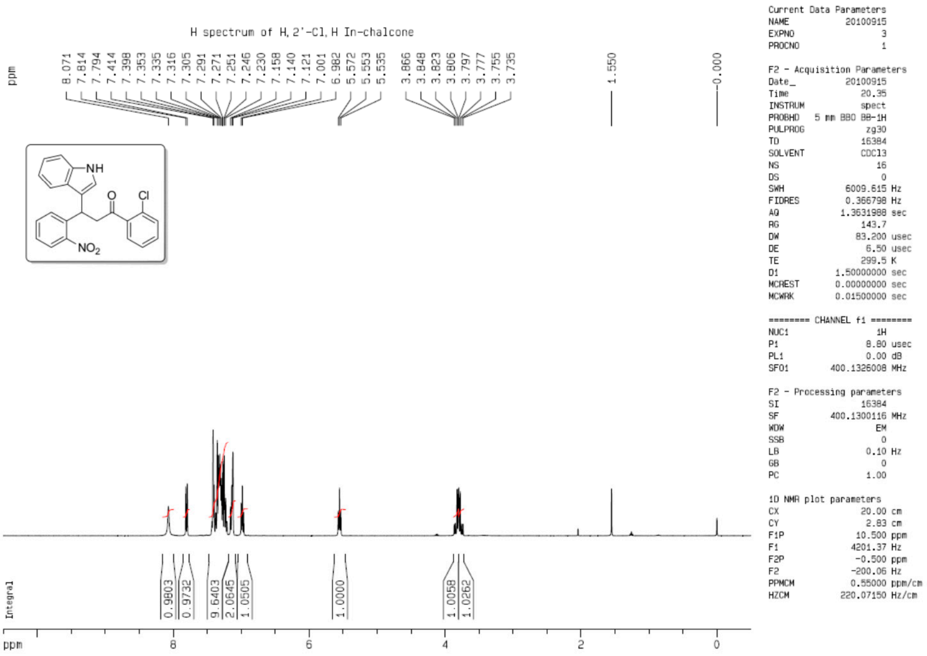

Figure S10. ^{13}C-NMR 1-(2-chlorophenyl)-3-(1*H*-indol-3-yl)-3-(2-nitrophenyl)propan-1-one (**3e**).

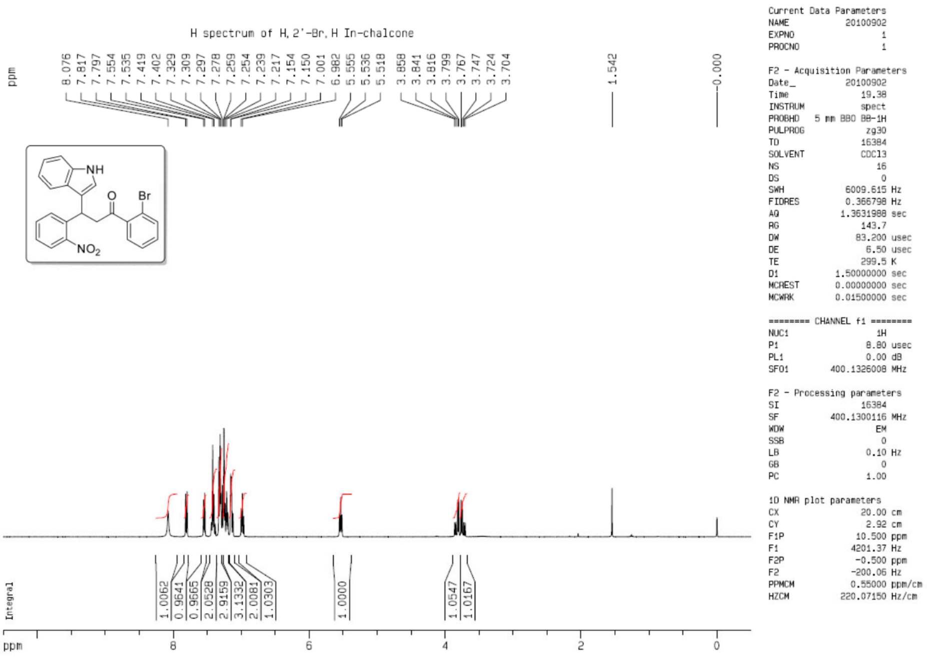

Figure S11. ^{1}H-NMR 1-(2-bromophenyl)-3-(1*H*-indol-3-yl)-3-(2-nitrophenyl)propan-1-one (**3f**).

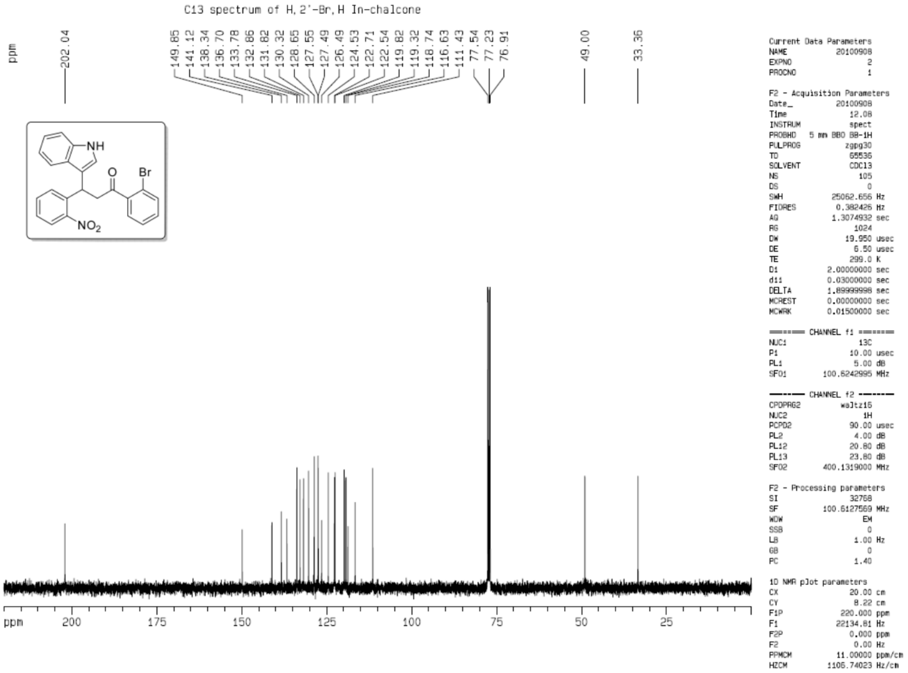

Figure S12. ^{13}C-NMR 1-(2-bromophenyl)-3-(1*H*-indol-3-yl)-3-(2-nitrophenyl)propan-1-one (**3f**).

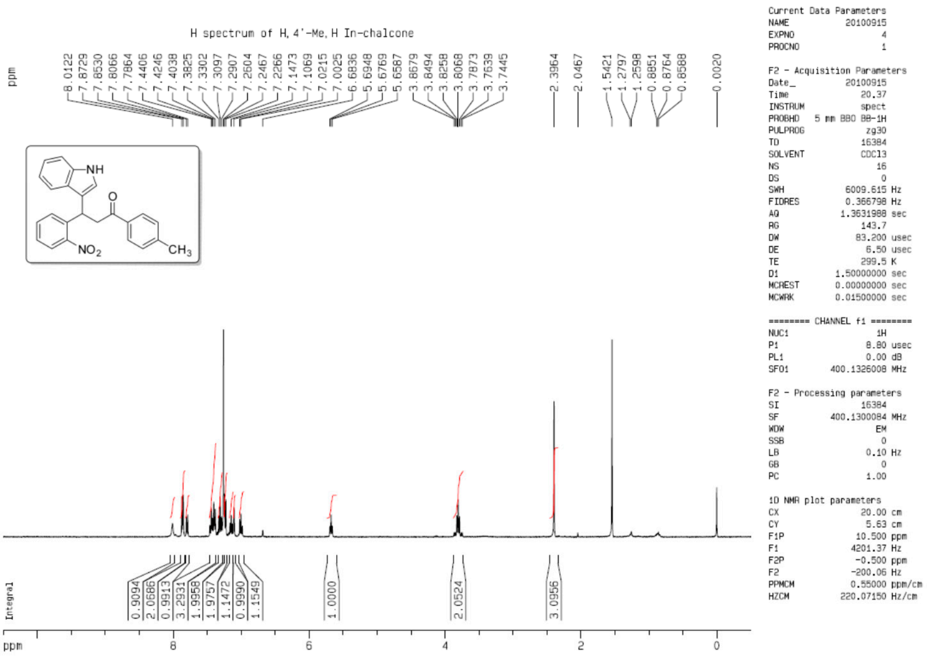

Figure S13. ^{1}H-NMR 3-(1*H*-indol-3-yl)-3-(2-nitrophenyl)-1-*p*-tolylpropan-1-one (**3g**).

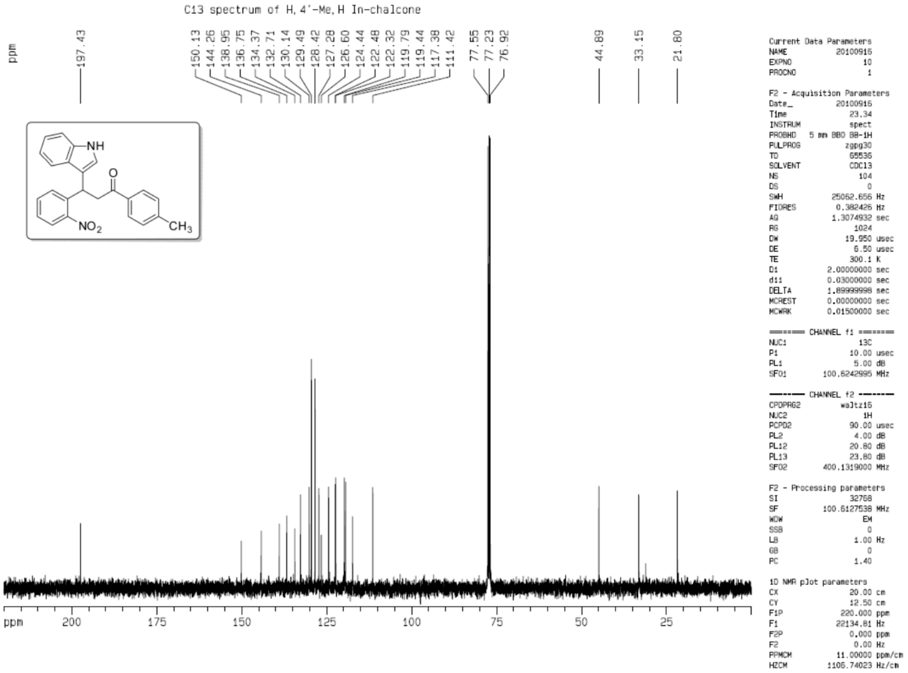

Figure S14. ^{13}C-NMR 3-(1*H*-indol-3-yl)-3-(2-nitrophenyl)-1-p-tolylpropan-1-one (**3g**).

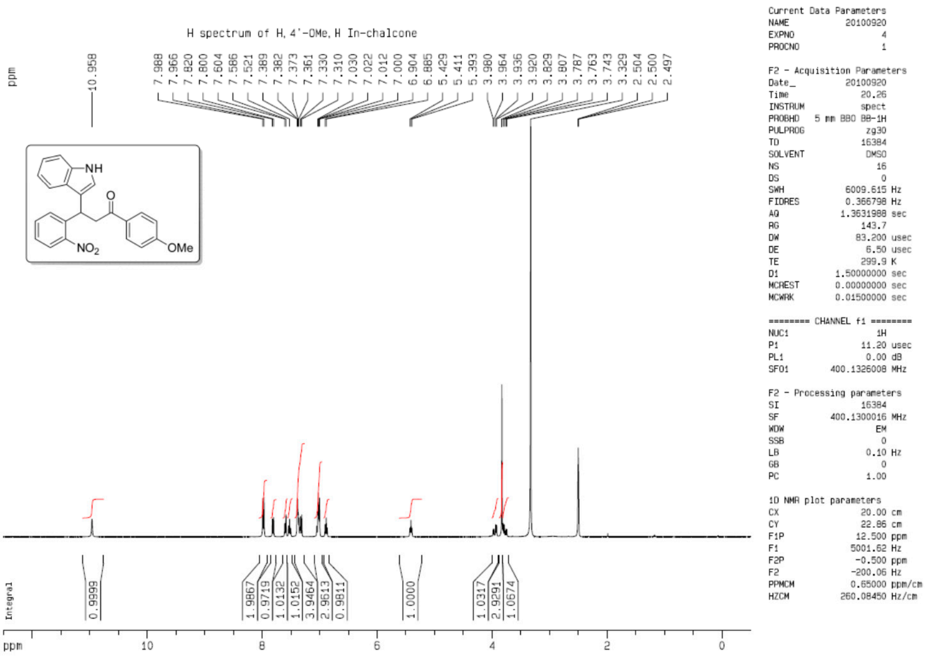

Figure S15. ^{1}H-NMR 3-(1*H*-Indol-3-yl)-1-(4-methoxyphenyl)-3-(2-nitrophenyl)propan-1-one (**3h**).

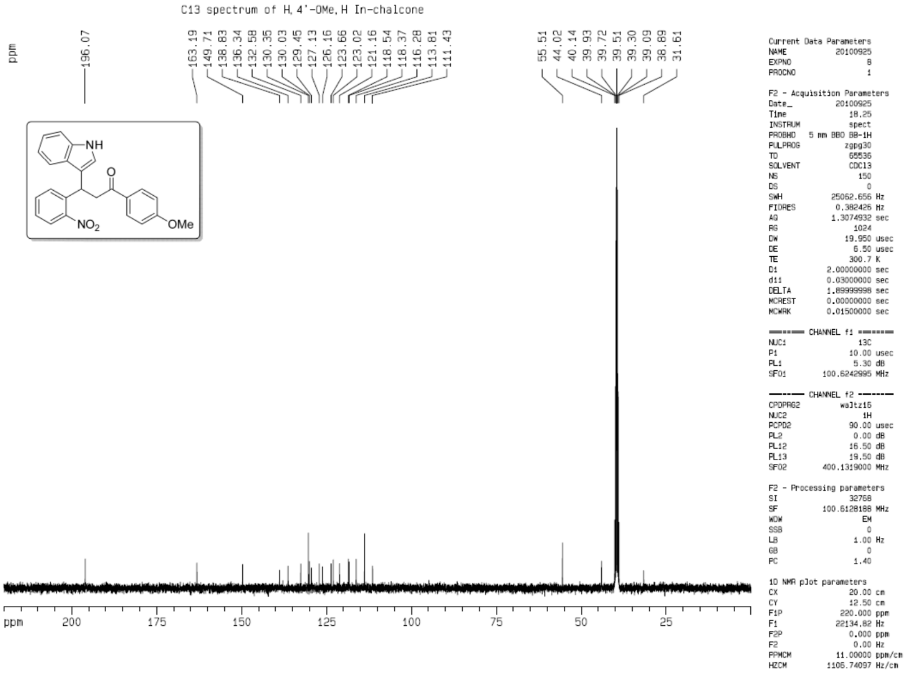

Figure S16. ^{13}C-NMR3-(1*H*-Indol-3-yl)-1-(4-methoxyphenyl)-3-(2-nitrophenyl)propan-1-one (**3h**).

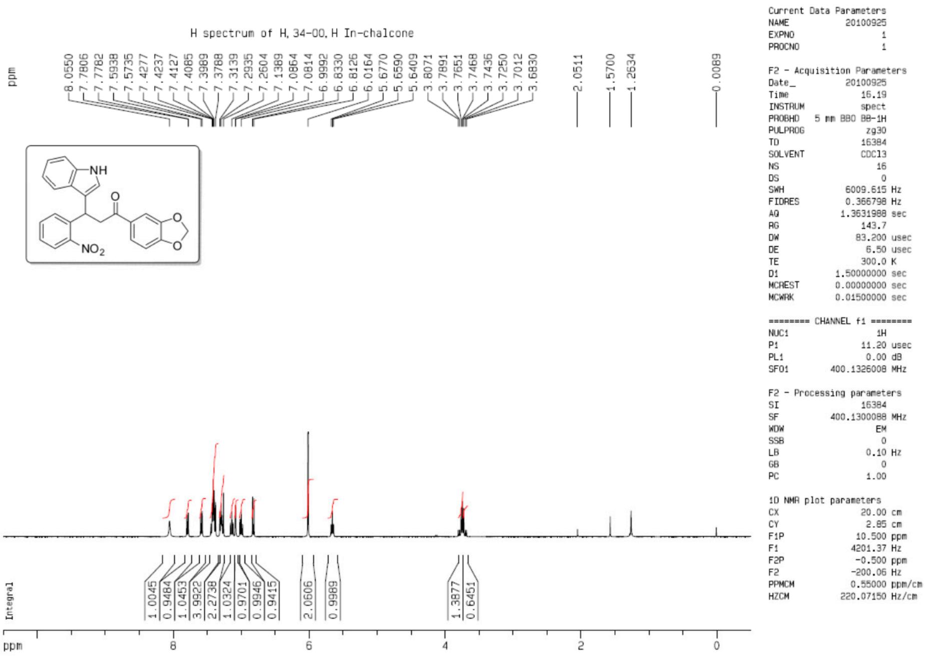

Figure S17. ^{1}H-NMR 1-(Benzo[d][1,3]dioxol-5-yl)-3-(1*H*-indol-3-yl)-3-(2-nitrophenyl)propan-1-one (**3i**).

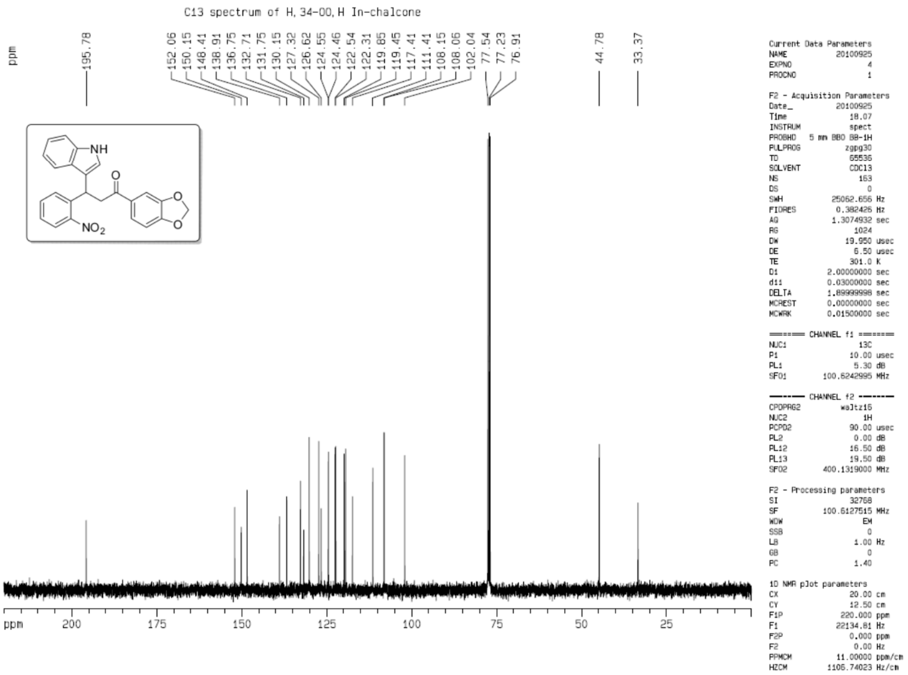

Figure S18. ^{13}C-NMR 1-(Benzo[d][1,3]dioxol-5-yl)-3-(1*H*-indol-3-yl)-3-(2-nitrophenyl)propan-1-one (**3i**).

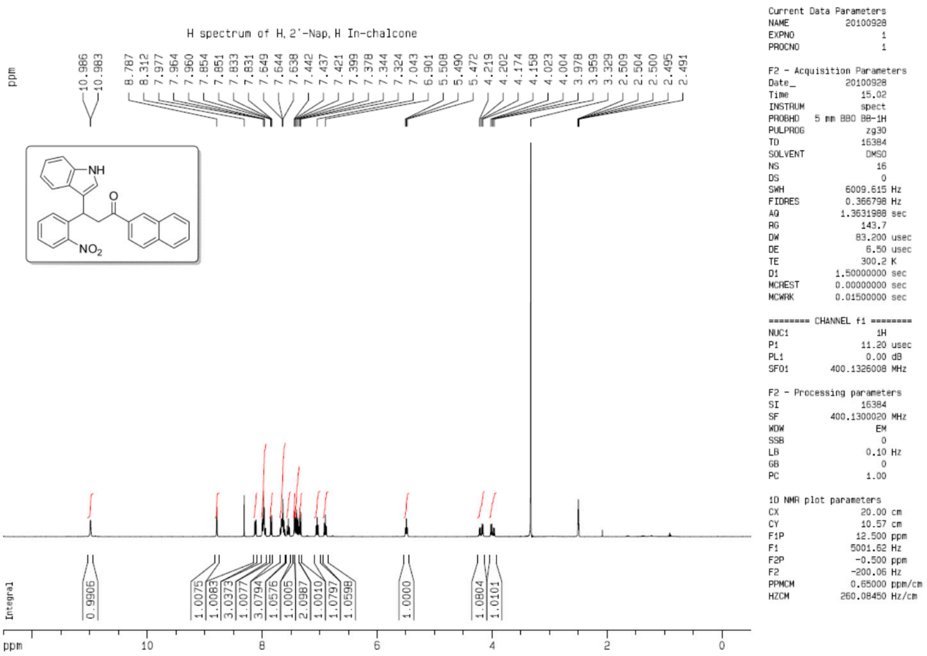

Figure S19. ^{1}H-NMR 3-(1*H*-Indol-3-yl)-1-(naphthalen-2-yl)-3-(2-nitrophenyl)propan-1-one (**3j**).

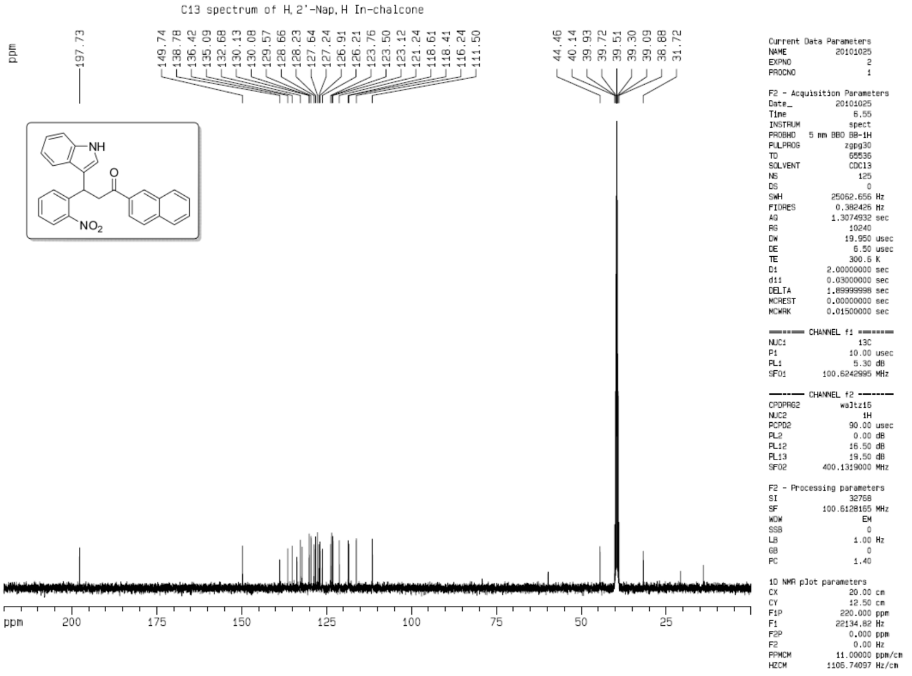

Figure S20. ^{13}C-NMR 3-(1*H*-Indol-3-yl)-1-(naphthalen-2-yl)-3-(2-nitrophenyl)propan-1-one (**3j**).

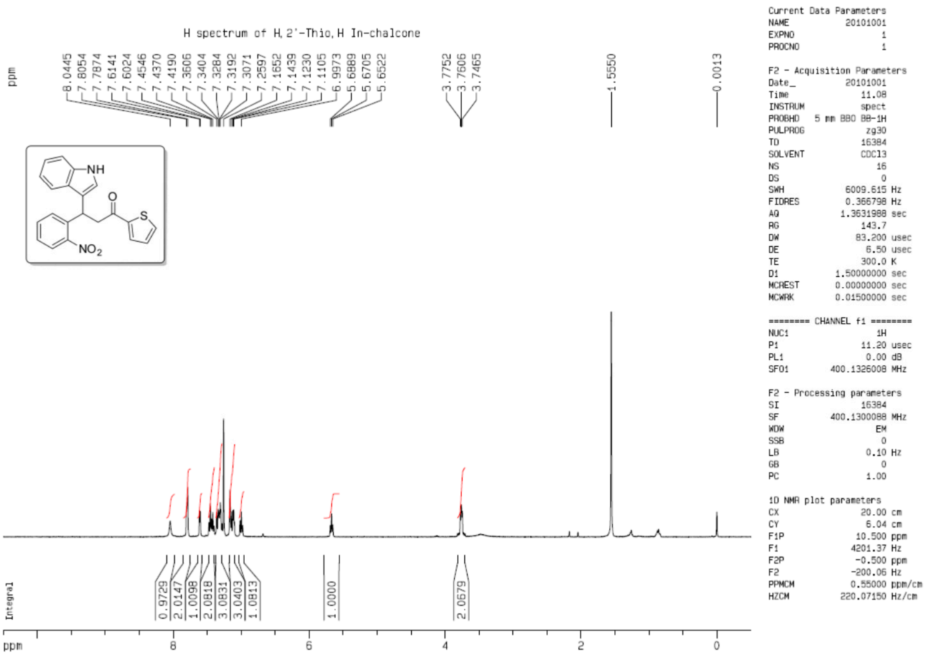

Figure S21. ^{1}H-NMR 3-(1*H*-Indol-3-yl)-3-(2-nitrophenyl)-1-(thiophen-2-yl)propan-1-one (**3k**).

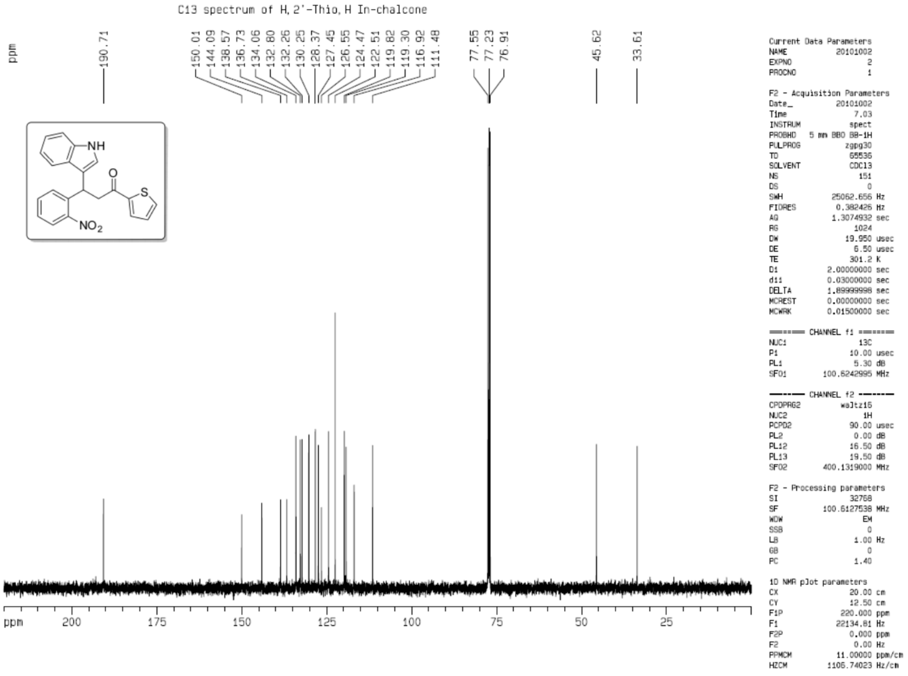

Figure S22. ^{13}C-NMR 3-(1*H*-Indol-3-yl)-3-(2-nitrophenyl)-1-(thiophen-2-yl)propan-1-one (**3k**).

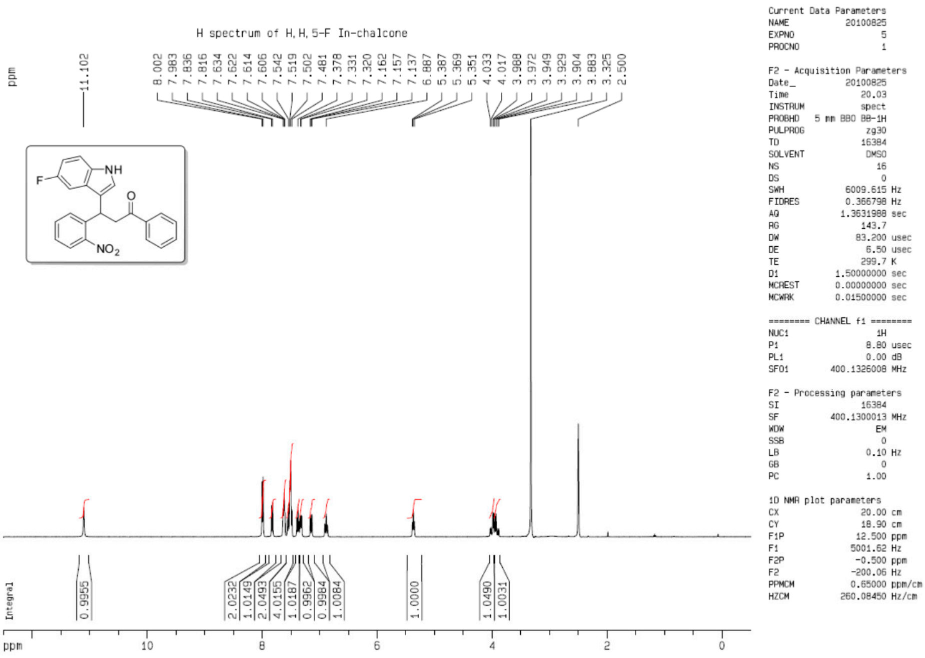

Figure S23. ^{1}H-NMR 3-(5-Fluoro-1*H*-indol-3-yl)-3-(2-nitrophenyl)-1-phenylpropan-1-one (**3l**).

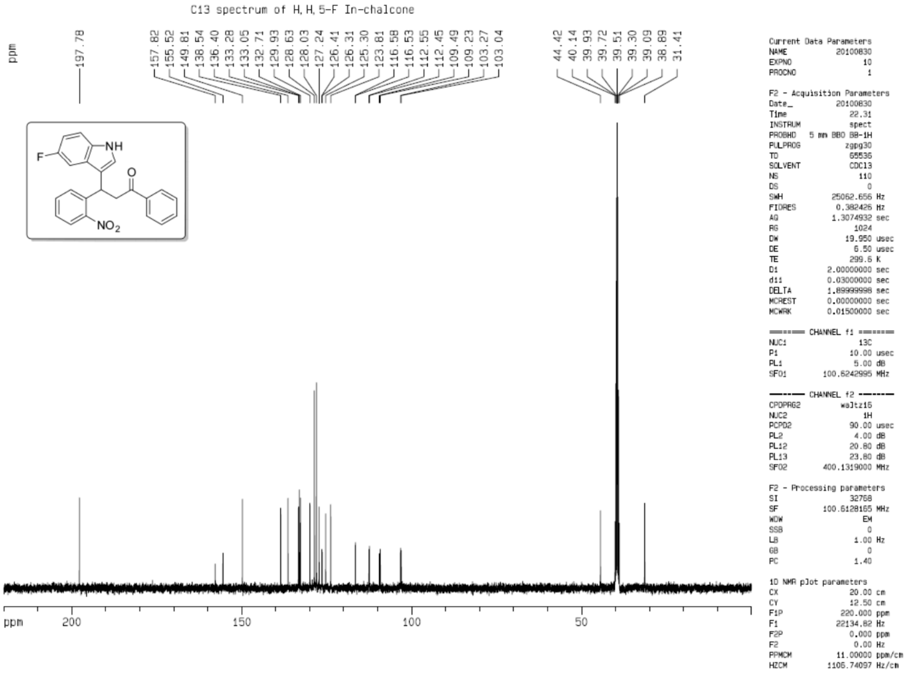

Figure S24. ^{1}H-NMR 3-(5-Fluoro-1*H*-indol-3-yl)-3-(2-nitrophenyl)-1-phenylpropan-1-one (**3l**).

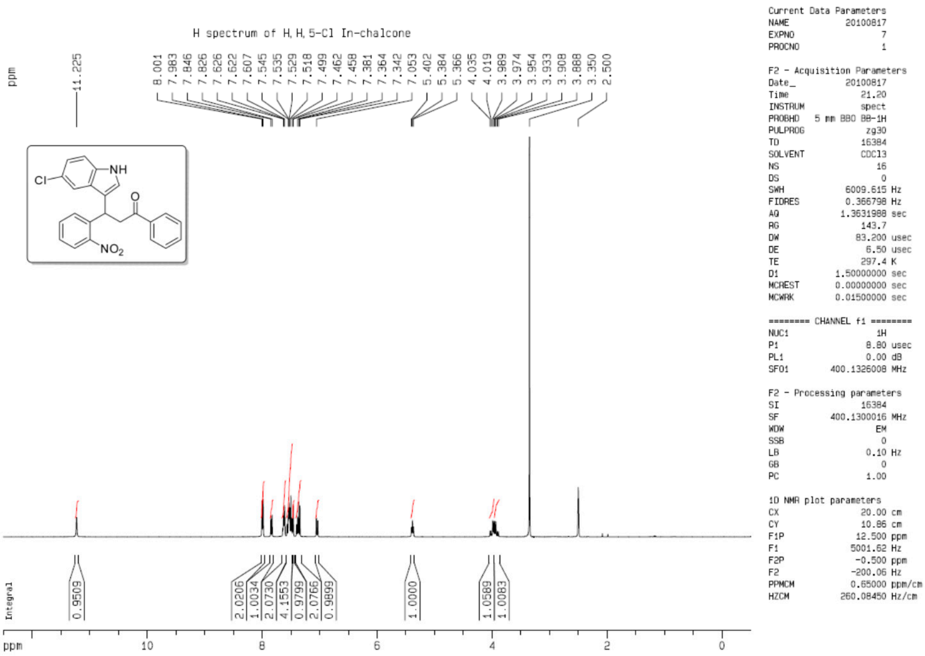

Figure S25. 3-(5-Chloro-1*H*-indol-3-yl)-3-(2-nitrophenyl)-1-phenylpropan-1-one (**3m**).

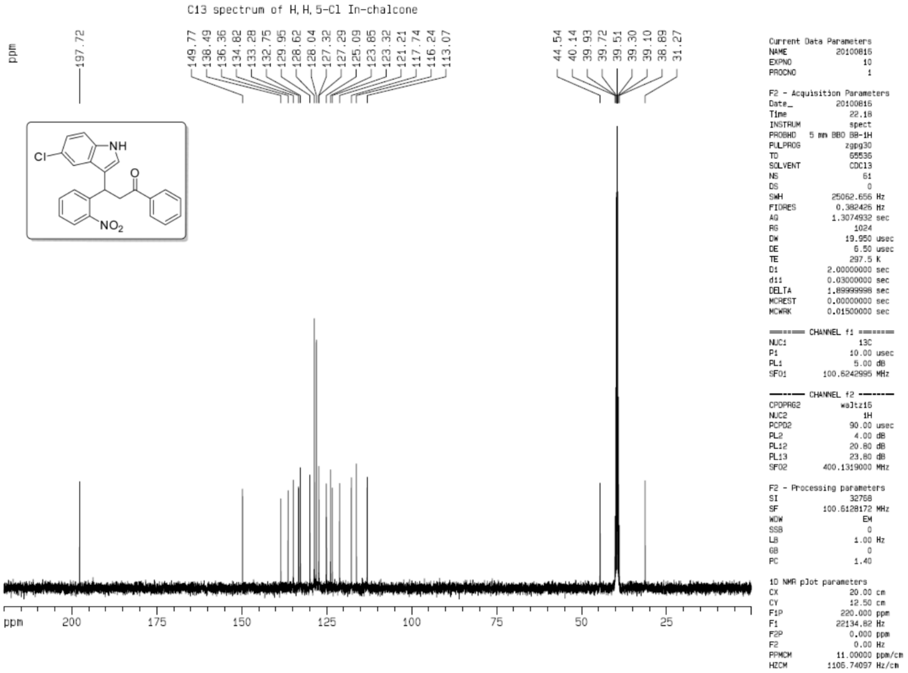

Figure S26. 3-(5-Chloro-1*H*-indol-3-yl)-3-(2-nitrophenyl)-1-phenylpropan-1-one (**3m**).

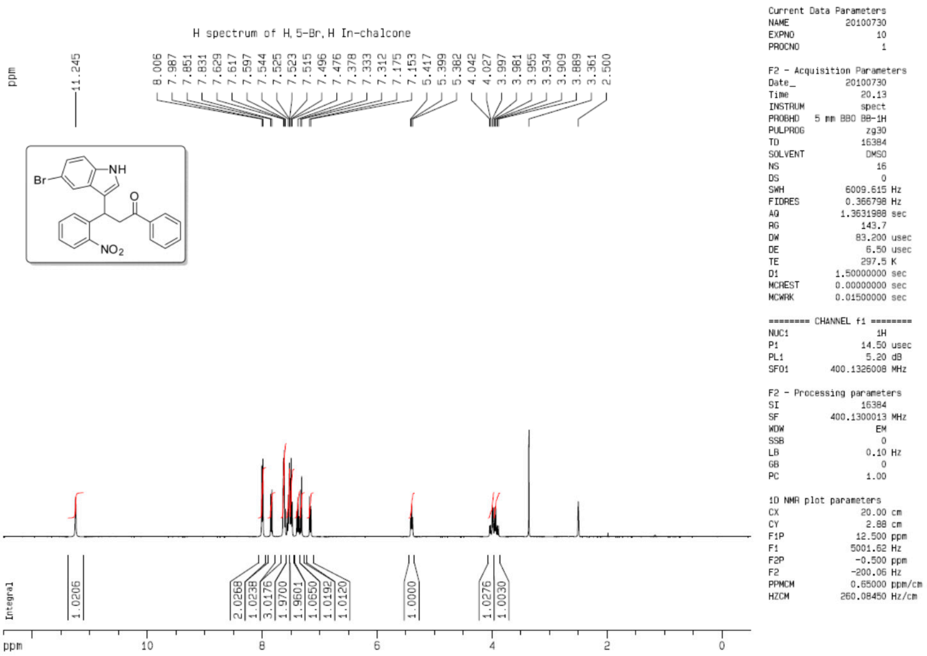

Figure S27. 3-(5-Bromo-1*H*-indol-3-yl)-3-(2-nitrophenyl)-1-phenylpropan-1-one (**3n**).

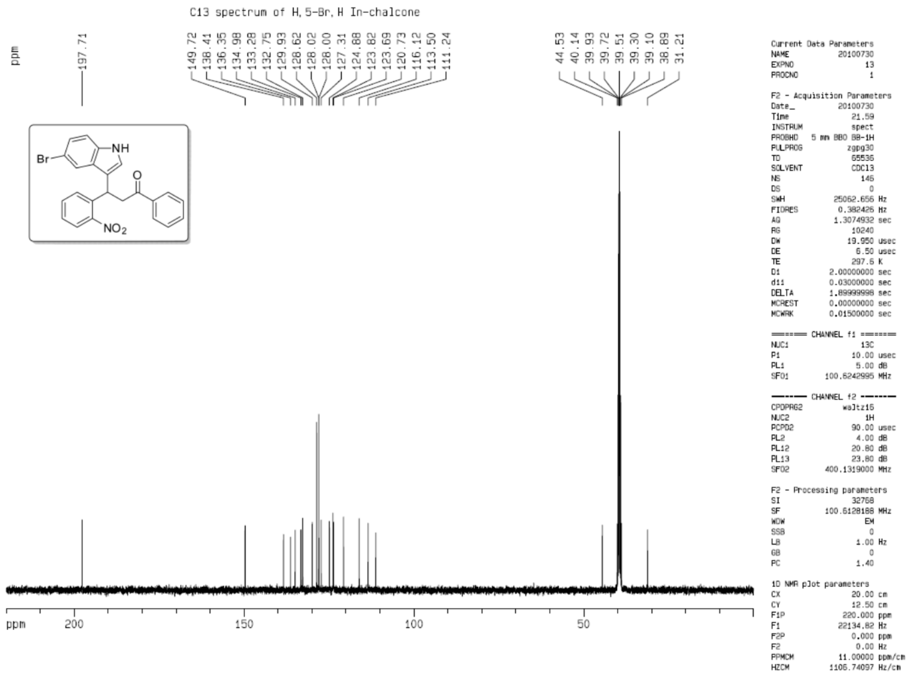

Figure S28. 3-(5-Bromo-1*H*-indol-3-yl)-3-(2-nitrophenyl)-1-phenylpropan-1-one (**3n**).

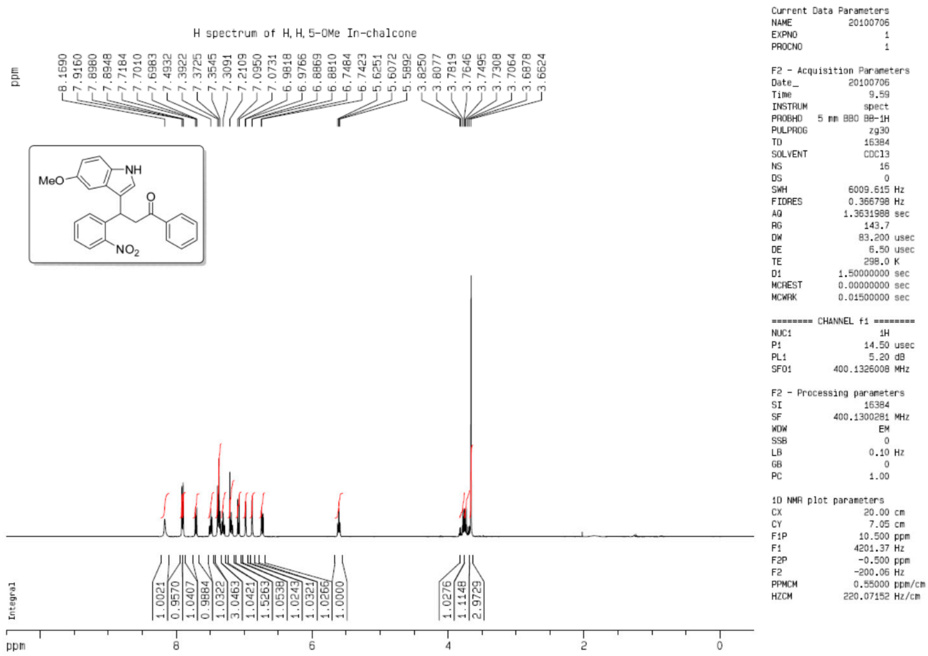

Figure S29. 3-(5-Methoxy-1*H*-indol-3-yl)-3-(2-nitrophenyl)-1-phenylpropan-1-one (**3o**).

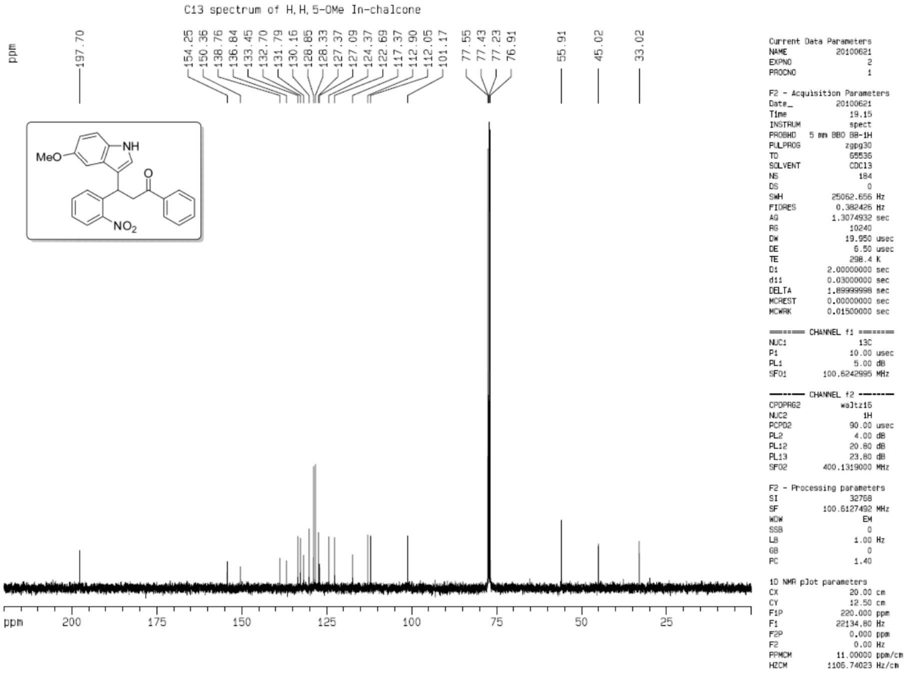

Figure S30. 3-(5-Methoxy-1*H*-indol-3-yl)-3-(2-nitrophenyl)-1-phenylpropan-1-one (**3o**).

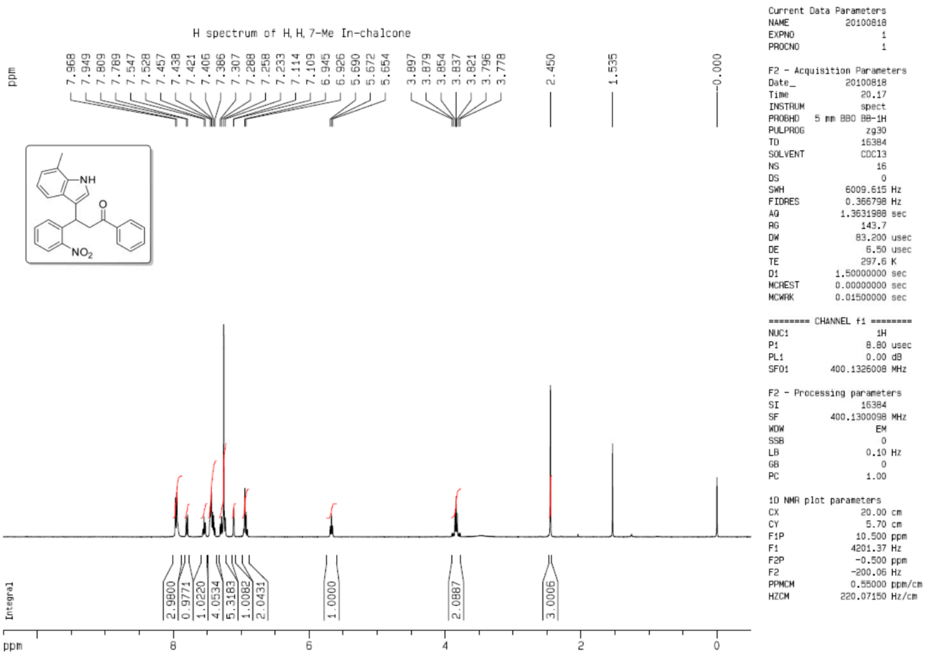

Figure S31. ^{1}H-NMR 3-(7-Methyl-1*H*-indol-3-yl)-3-(2-nitrophenyl)-1-phenylpropan-1-one (**3p**).

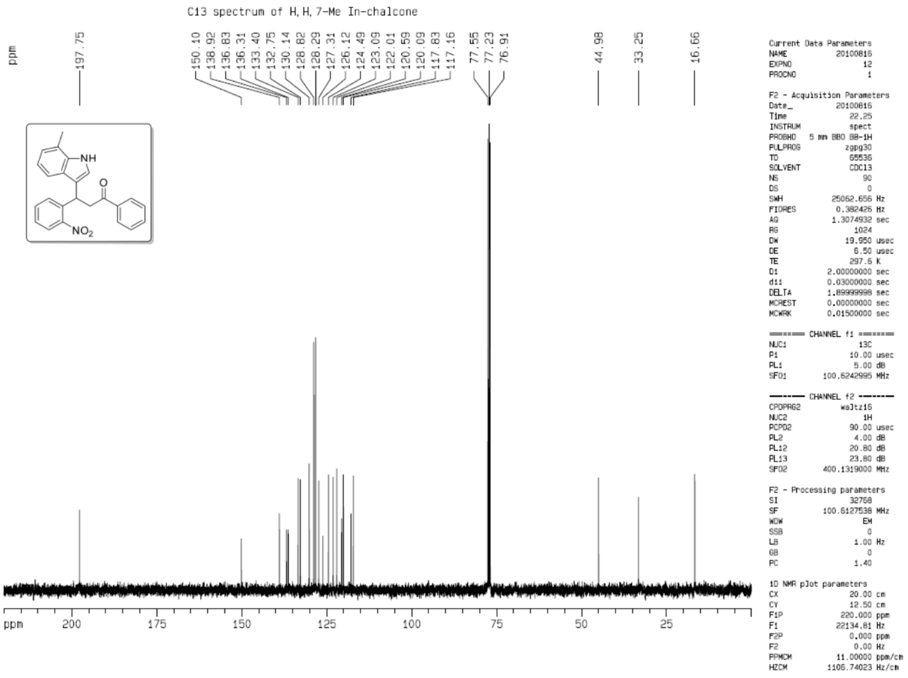

Figure S32. ^{1}H-NMR 3-(7-Methyl-1*H*-indol-3-yl)-3-(2-nitrophenyl)-1-phenylpropan-1-one (**3p**).

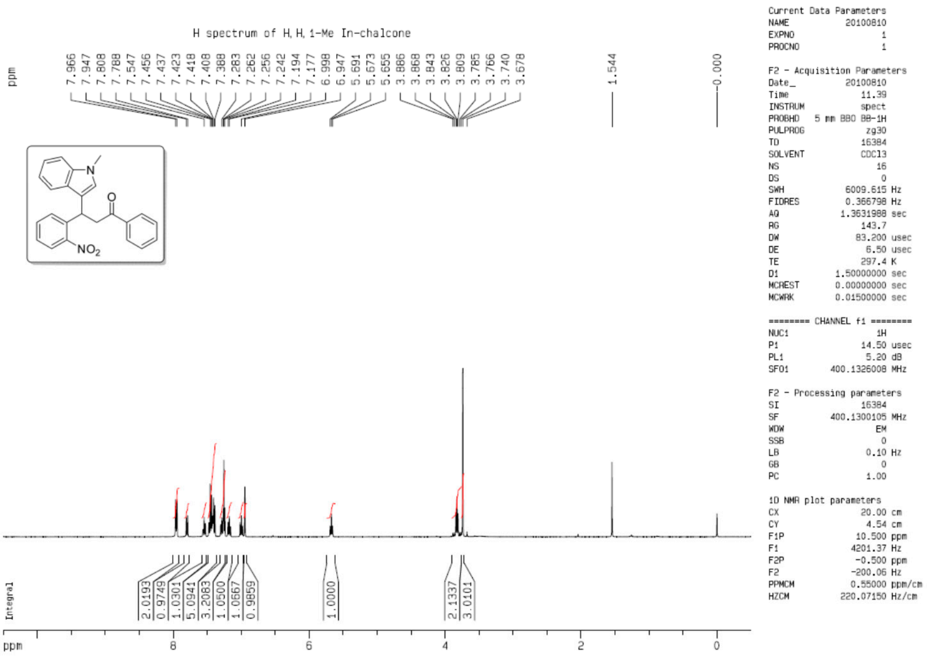

Figure S33. ^{1}H-NMR 3-(1-Methyl-1*H*-indol-3-yl)-3-(2-nitrophenyl)-1-phenylpropan-1-one (**3q**).

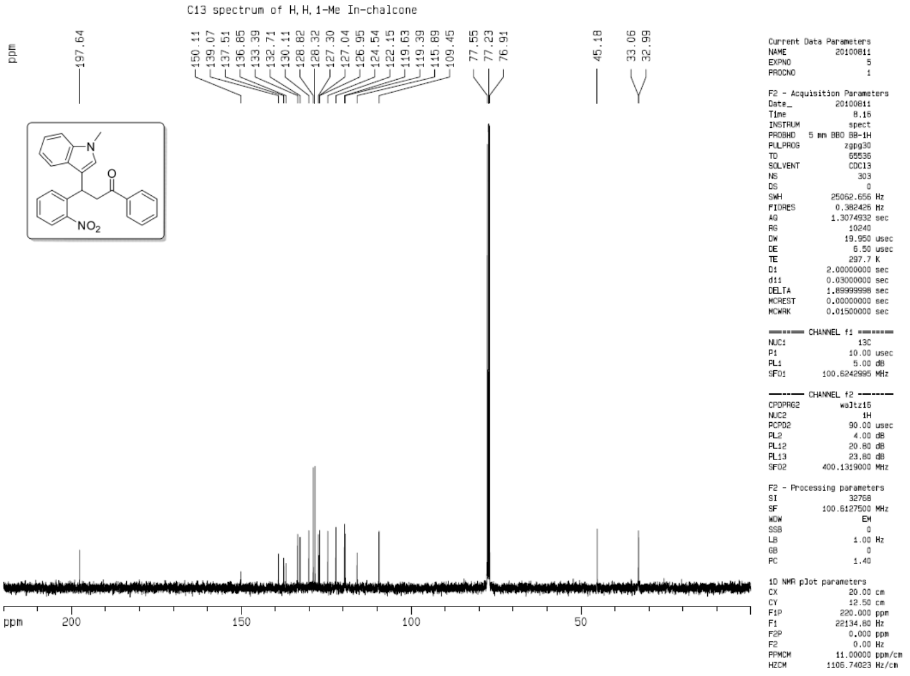

Figure S34. ^{13}C-NMR 3-(1-Methyl-1*H*-indol-3-yl)-3-(2-nitrophenyl)-1-phenylpropan-1-one (**3q**).

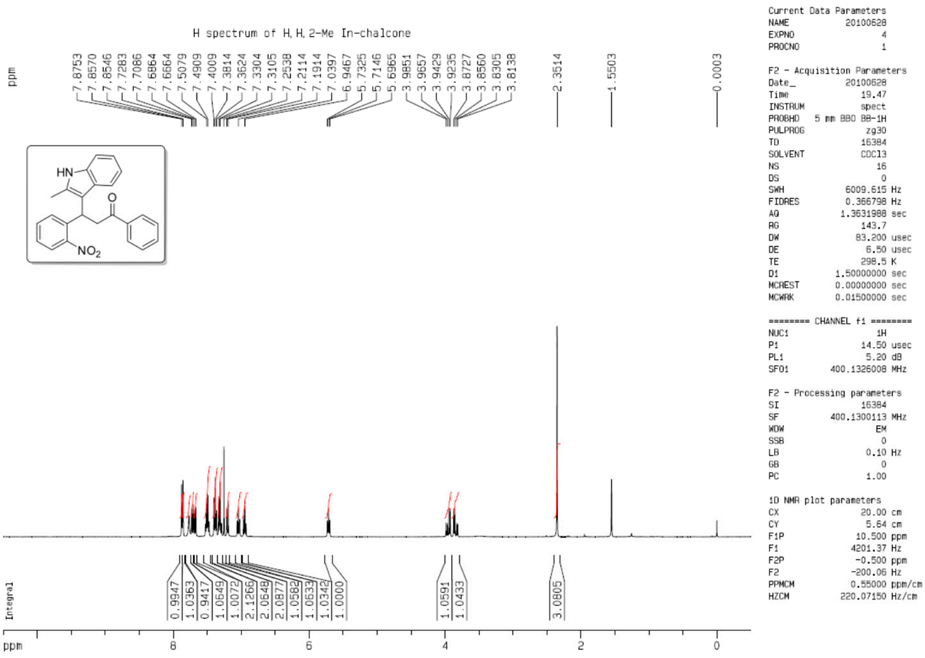

Figure S35. ^{1}H-NMR of 3-(2-Methyl-1*H*-indol-3-yl)-3-(2-nitrophenyl)-1-phenylpropan-1-one (**3r**).

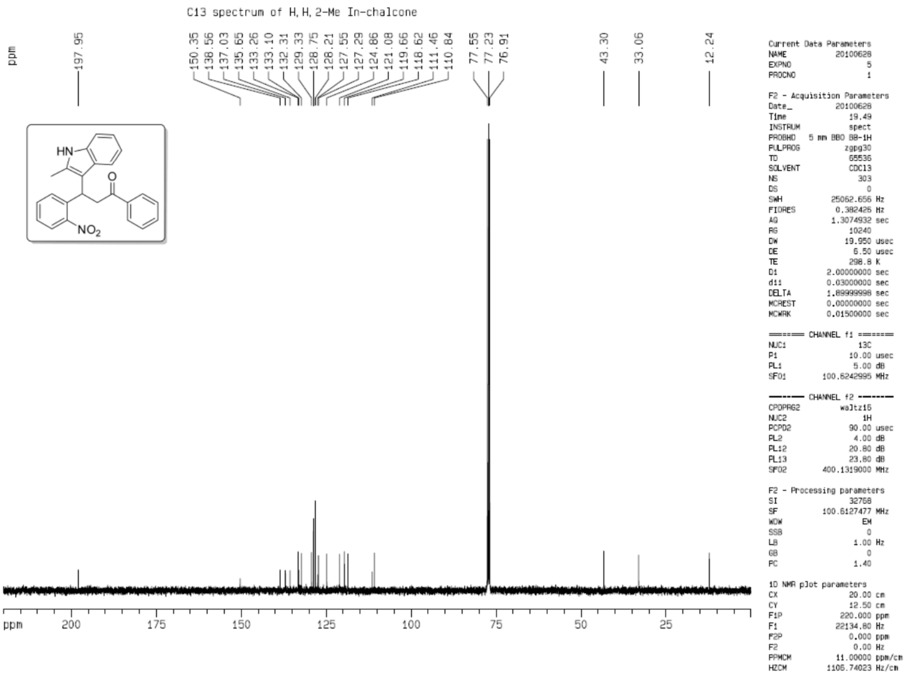

Figure S36. ^{13}C-NMR of 3-(2-Methyl-1H-indol-3-yl)-3-(2-nitrophenyl)-1-phenylpropan-1-one (**3r**).

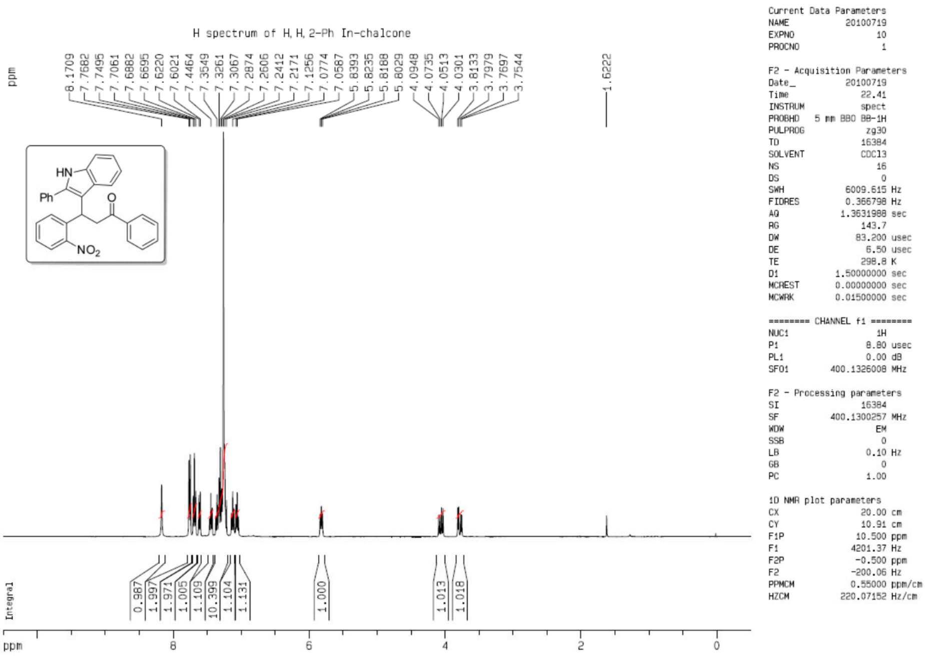

Figure S37. ^{1}H-NMR of 3-(2-Nitrophenyl)-1-phenyl-3-(2-phenyl-1*H*-indol-3-yl)propan-1-one (**3s**).

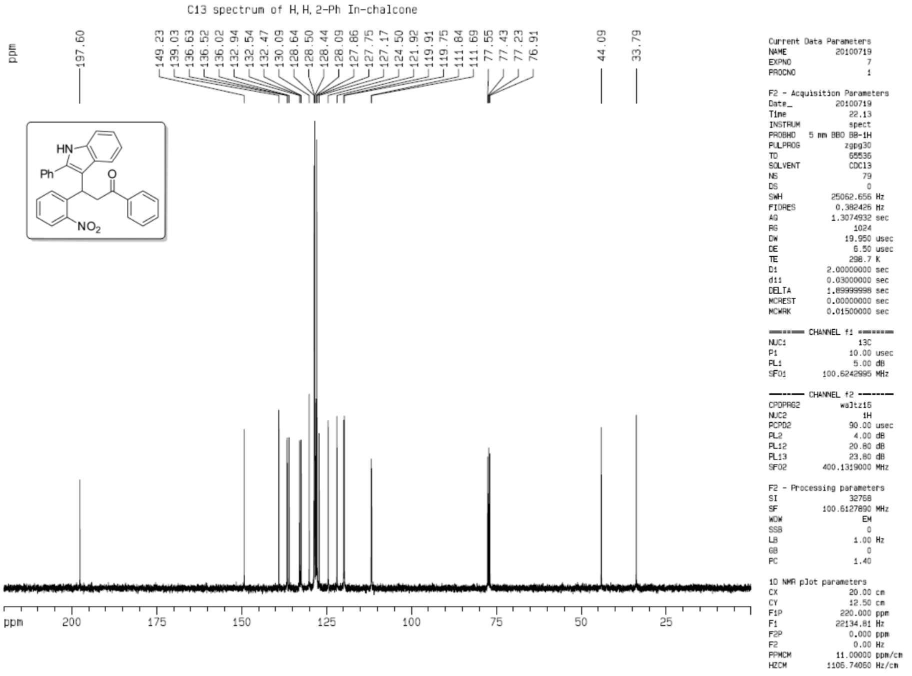

Figure S38. ^{13}C-NMR of 3-(2-Nitrophenyl)-1-phenyl-3-(2-phenyl-1*H*-indol-3-yl)propan-1-one (**3s**).

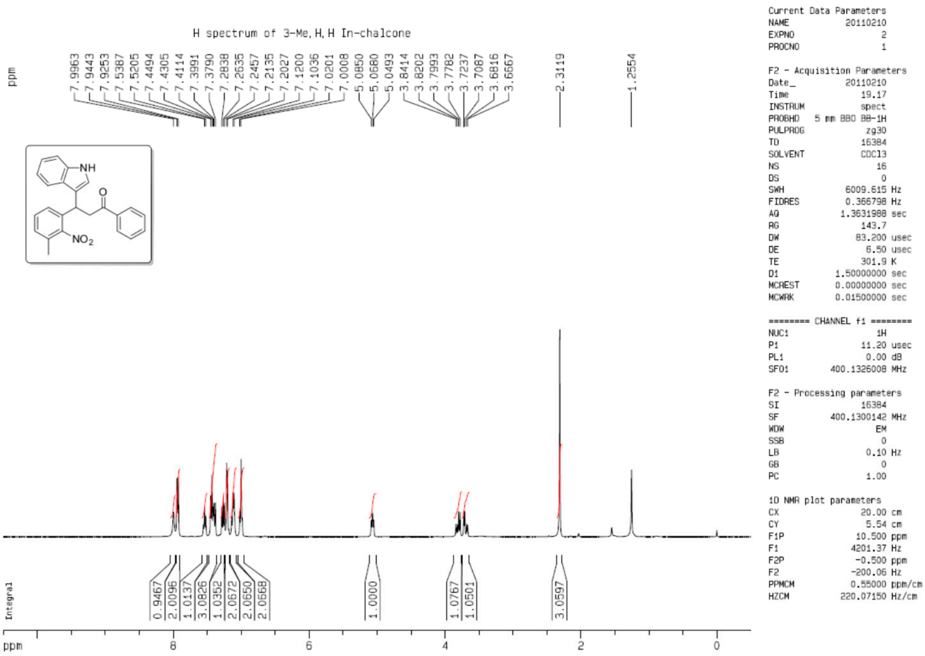

Figure S39. ^{13}C-NMR of 3-(1*H*-Indol-3-yl)-3-(3-methyl-2-nitrophenyl)-1-phenylpropan-1-one (**3t**).

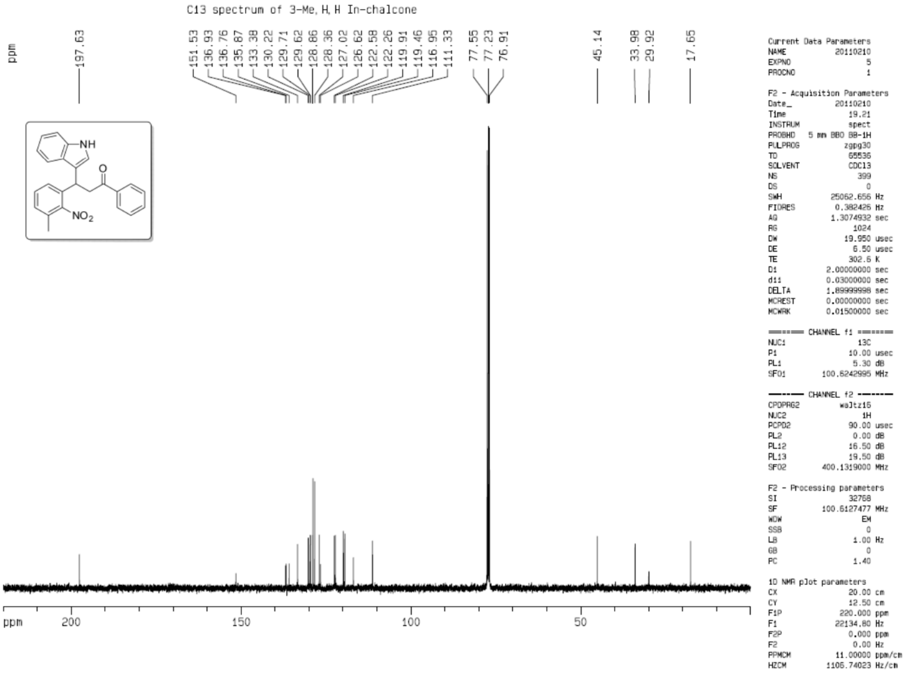

Figure S40. ^{13}C-NMR of 3-(1*H*-Indol-3-yl)-3-(3-methyl-2-nitrophenyl)-1-phenylpropan-1-one (**3t**).

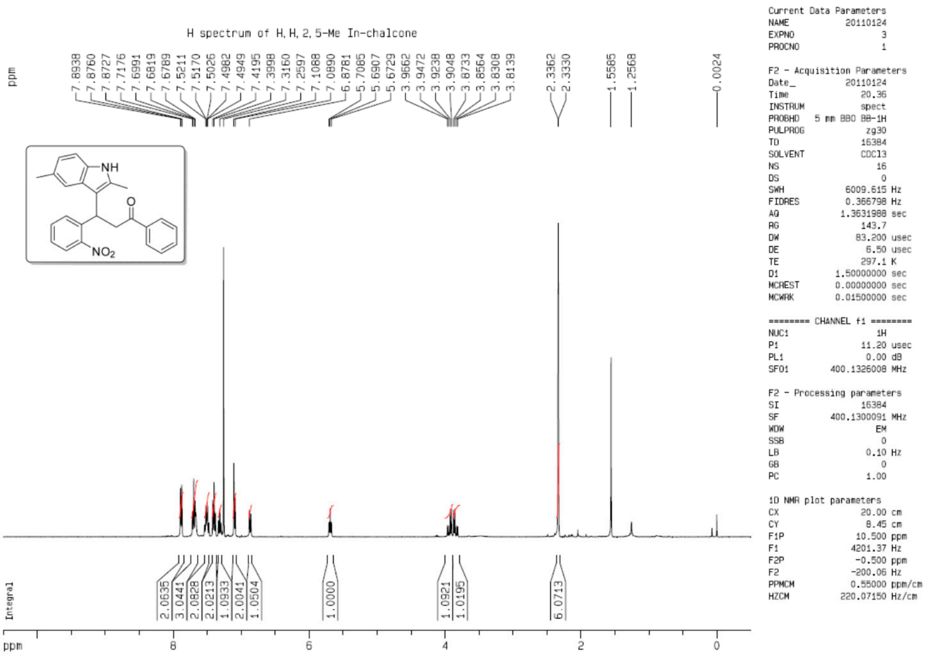

Figure S41. ^{1}H-NMR of 3-(2,5-Dimethyl-1*H*-indol-3-yl)-3-(2-nitrophenyl)-1-phenylpropan-1-one (**3u**).

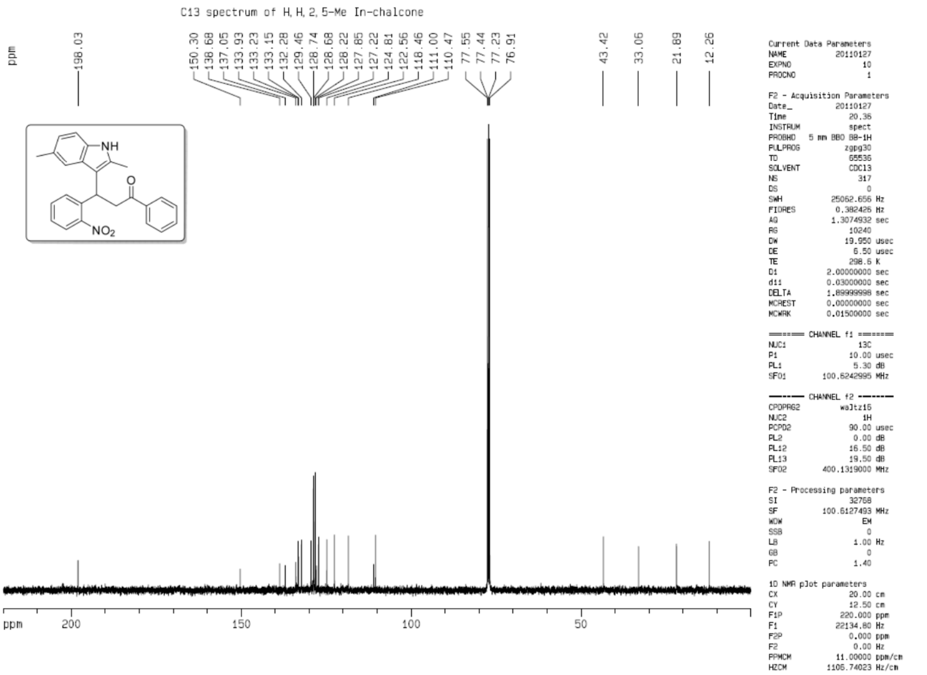

Figure S42. ^{13}C-NMR of 3-(2,5-Dimethyl-1*H*-indol-3-yl)-3-(2-nitrophenyl)-1-phenylpropan-1-one (**3u**).

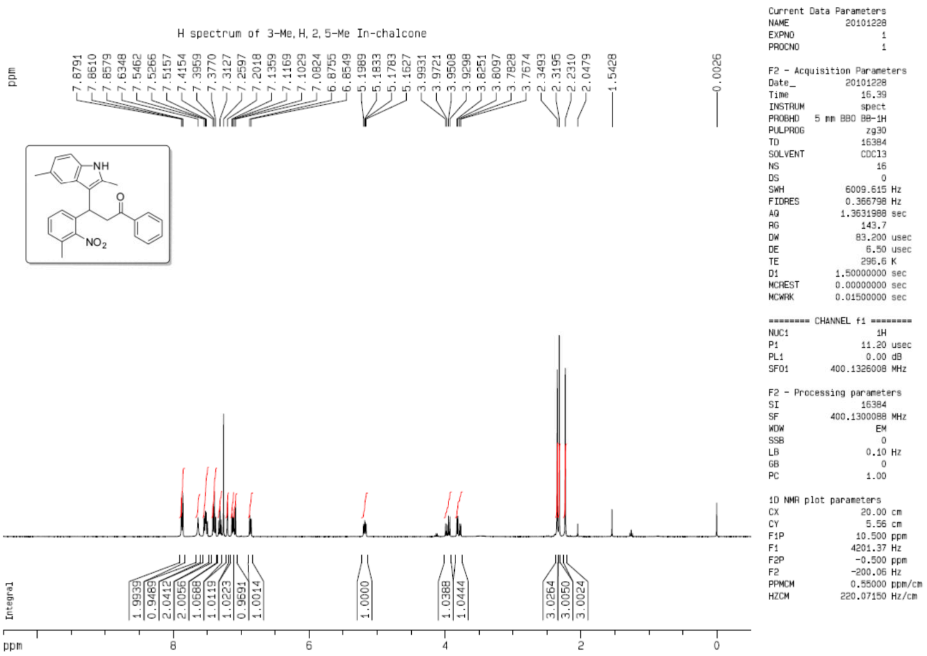

Figure S43. ^{1}H-NMR of 3-(2,5-Dimethyl-1*H*-indol-3-yl)-3-(3-methyl-2-nitrophenyl)-1-phenylpropan-1-one (**3v**).

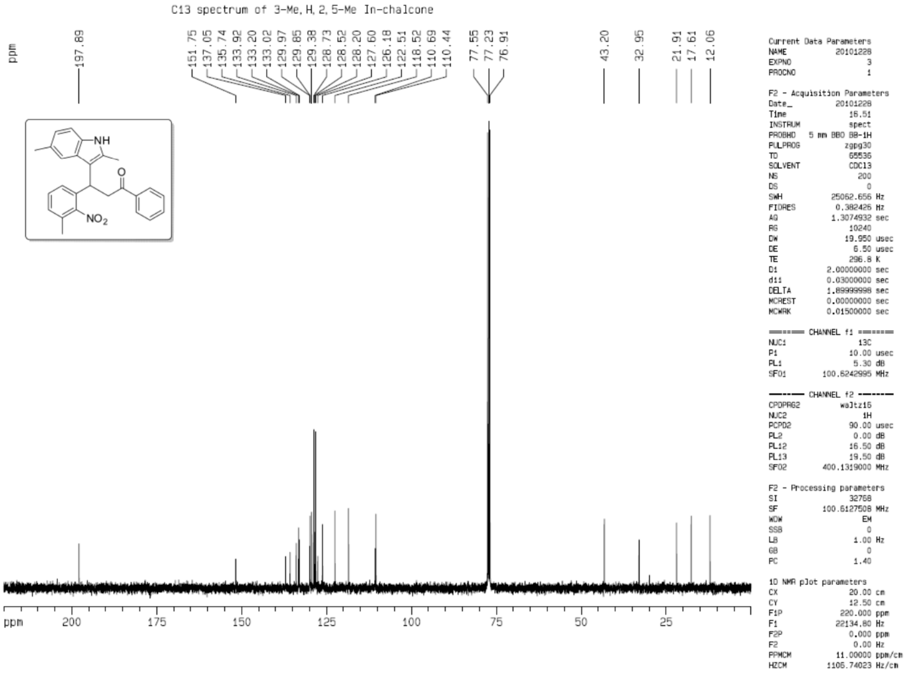

Figure S44. ^{13}C-NMR of 3-(2,5-Dimethyl-1*H*-indol-3-yl)-3-(3-methyl-2-nitrophenyl)-1-phenylpropan-1-one (**3v**).

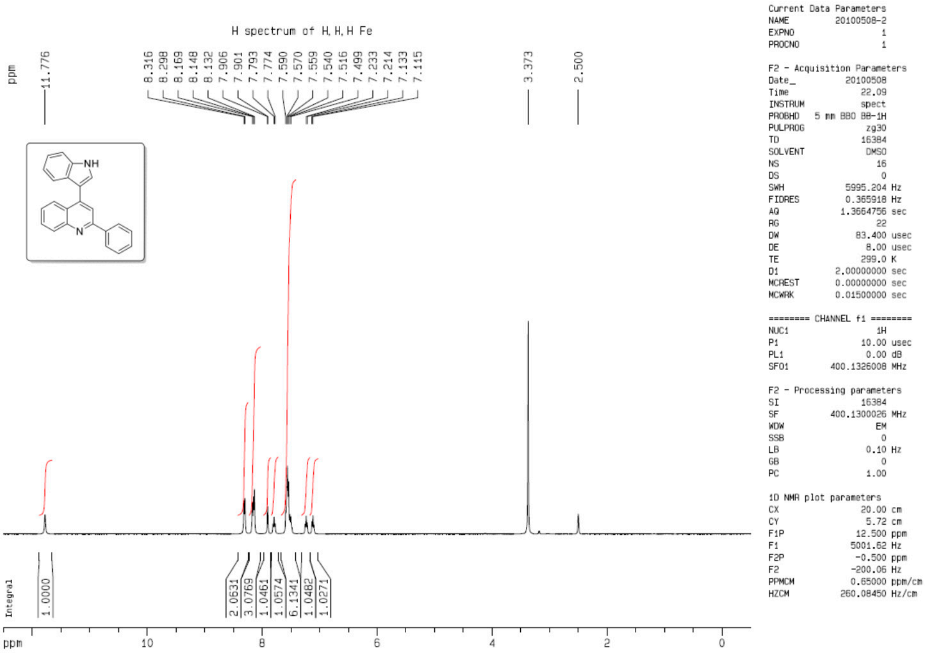

Figure S45. ^{1}H-NMR of 4-(1*H*-Indol-3-yl)-2-phenylquinoline (**4a**).

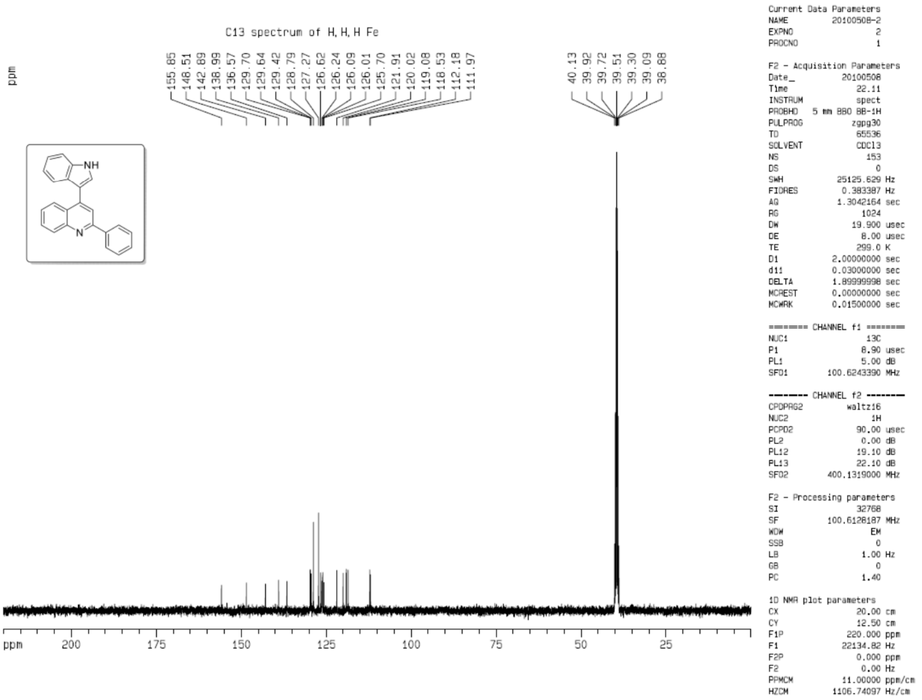

Figure S46. ^{13}C-NMR of 4-(1*H*-Indol-3-yl)-2-phenylquinoline (**4a**).

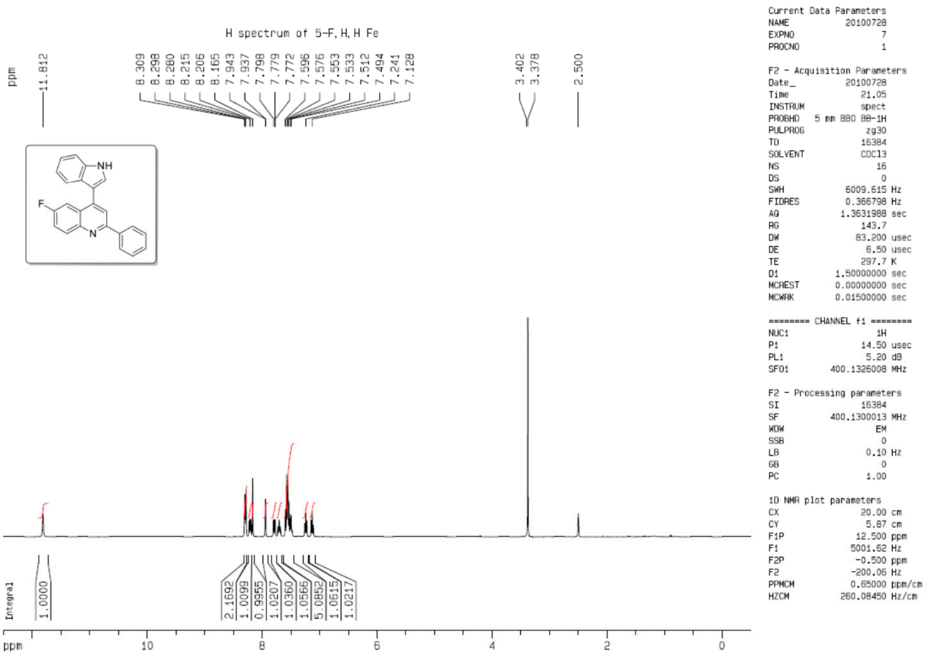

Figure S47. ^{1}H-NMR 6-Fluoro-4-(1*H*-indol-3-yl)-2-phenylquinoline (**4b**).

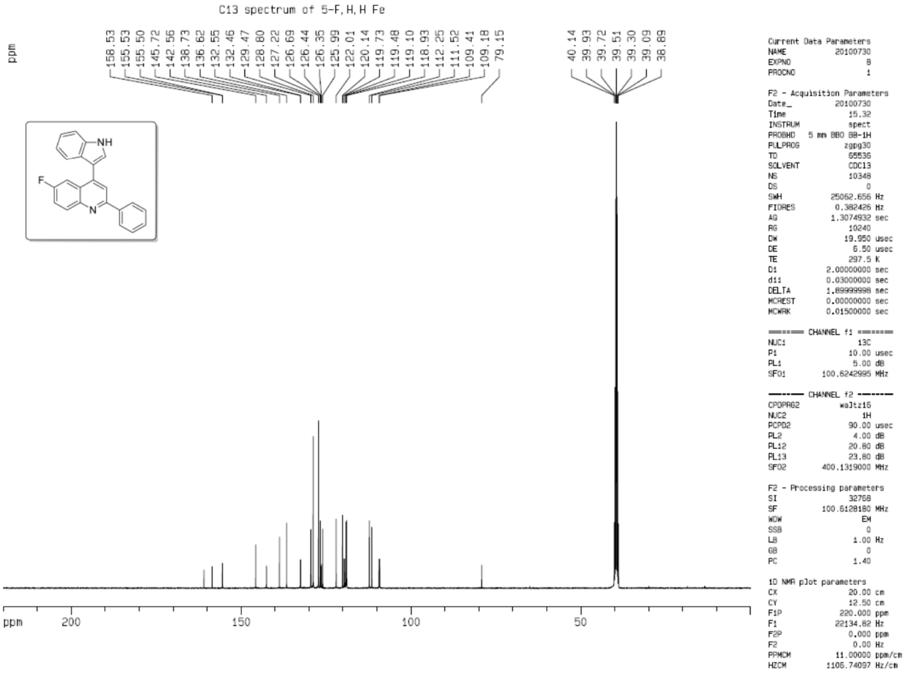

Figure S48. ^{13}C-NMR 6-Fluoro-4-(1*H*-indol-3-yl)-2-phenylquinoline (**4b**).

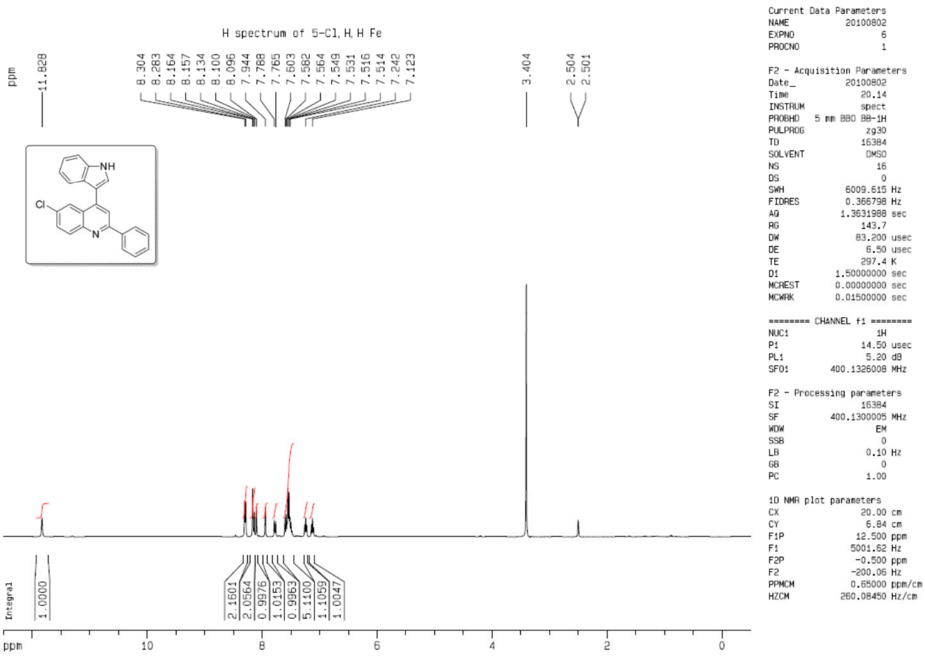

Figure S49. ^{1}H-NMR 6-Chloro-4-(1*H*-indol-3-yl)-2-phenylquinoline (**4c**).

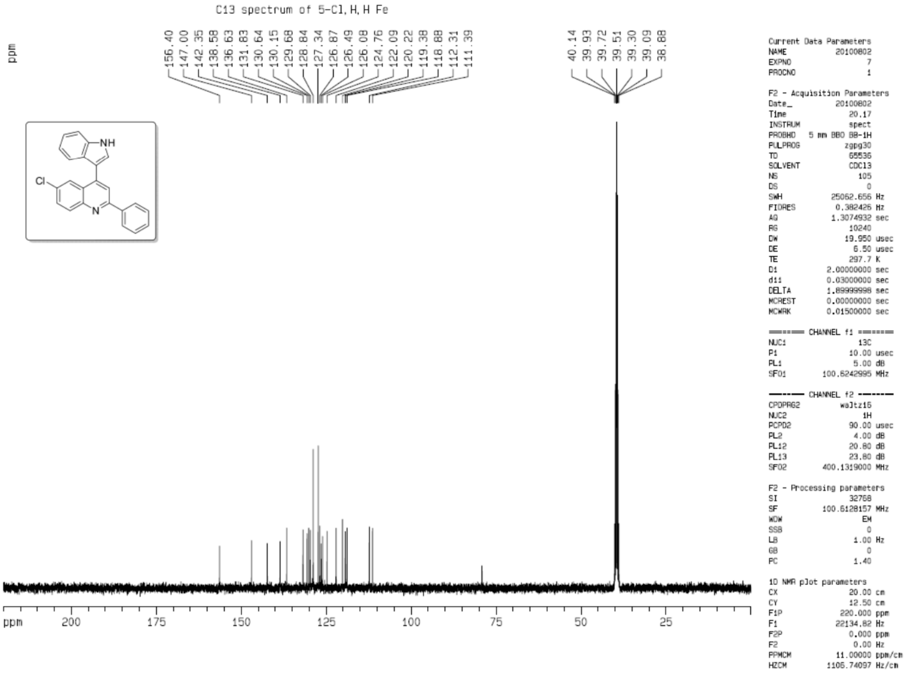

Figure S50. ^{13}C-NMR 6-Chloro-4-(1*H*-indol-3-yl)-2-phenylquinoline (**4c**).

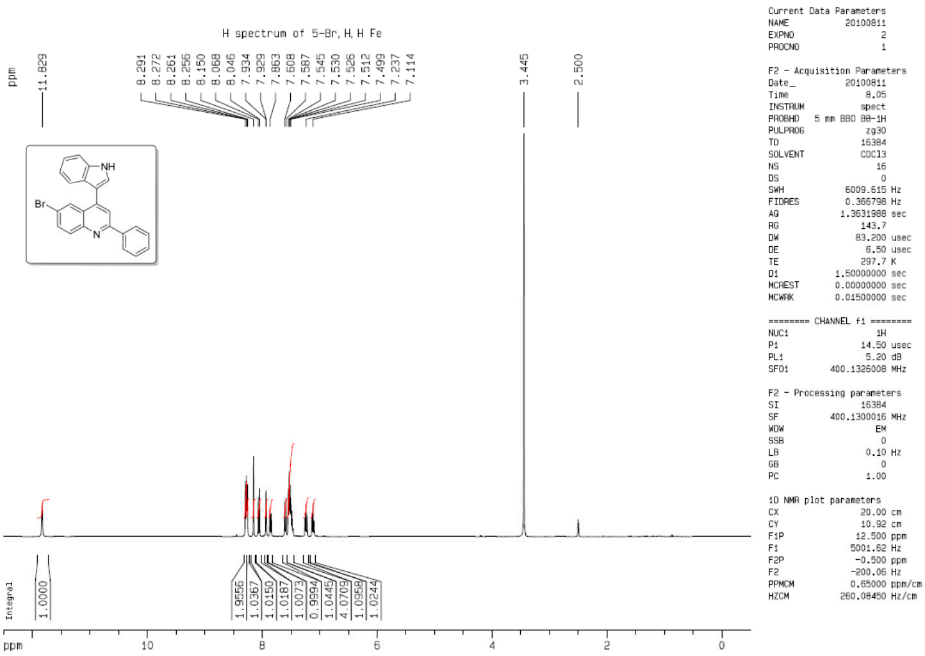

Figure S51. ^{1}H-NMR 6-Bromo-4-(1*H*-indol-3-yl)-2-phenylquinoline (**4d**).

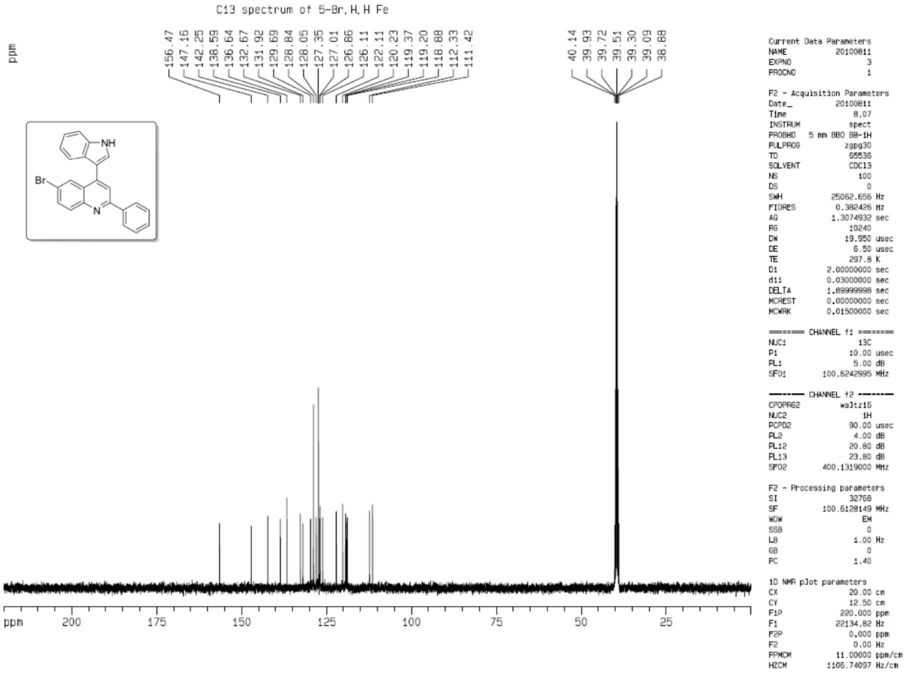

Figure S52. ^{13}C-NMR 6-Bromo-4-(1*H*-indol-3-yl)-2-phenylquinoline (**4d**).

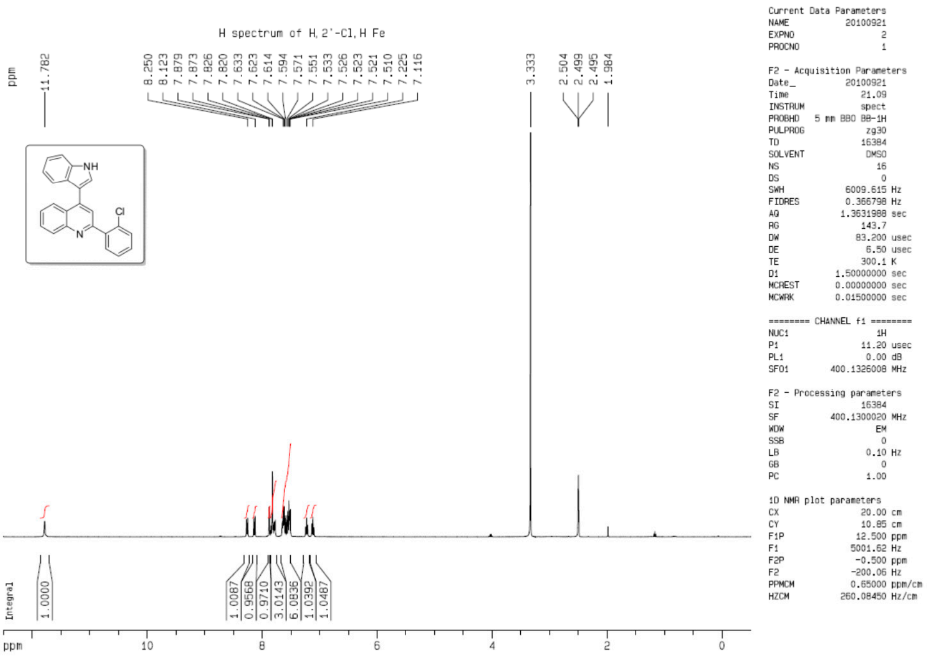

Figure S53. ^{1}H-NMR of 2-(2-Chlorophenyl)-4-(1*H*-indol-3-yl)quinoline (**4e**).

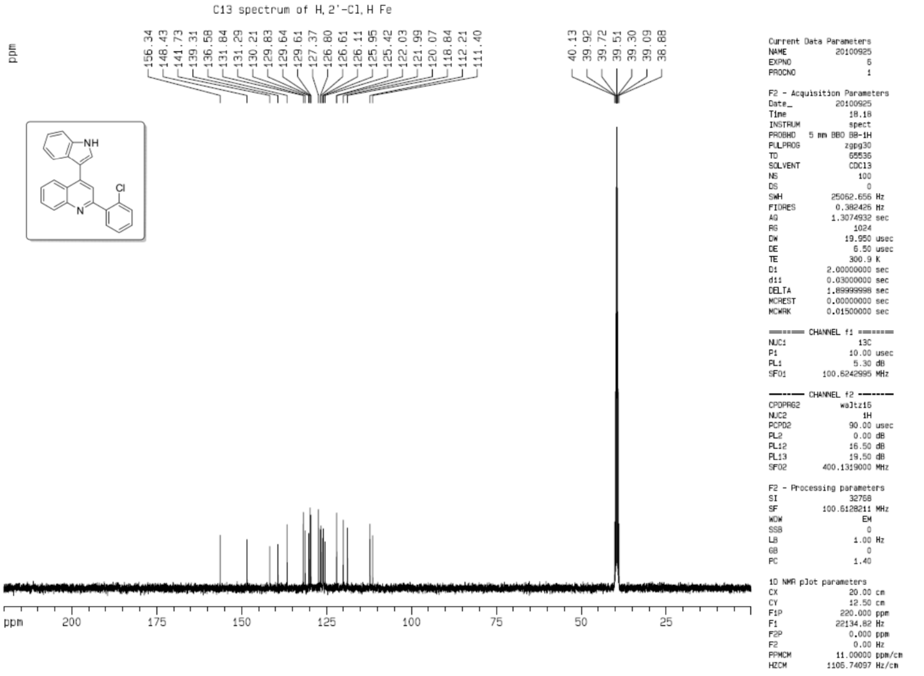

Figure S54. ^{13}C-NMR of 2-(2-Chlorophenyl)-4-(1*H*-indol-3-yl)quinoline (**4e**).

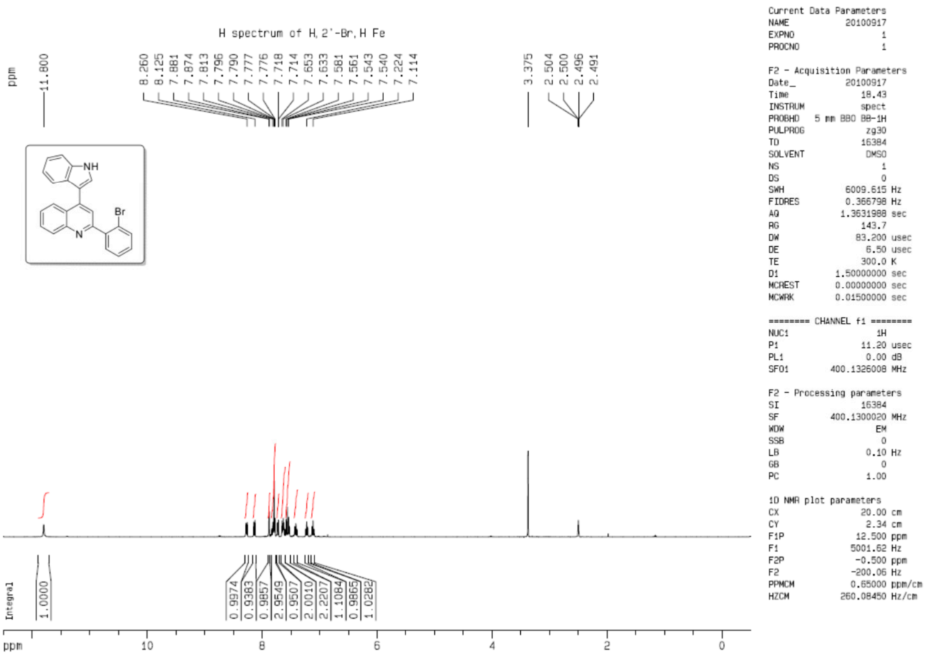

Figure S55. ^{1}H-NMR of 2-(2-Bromophenyl)-4-(1*H*-indol-3-yl)quinoline (**4f**).

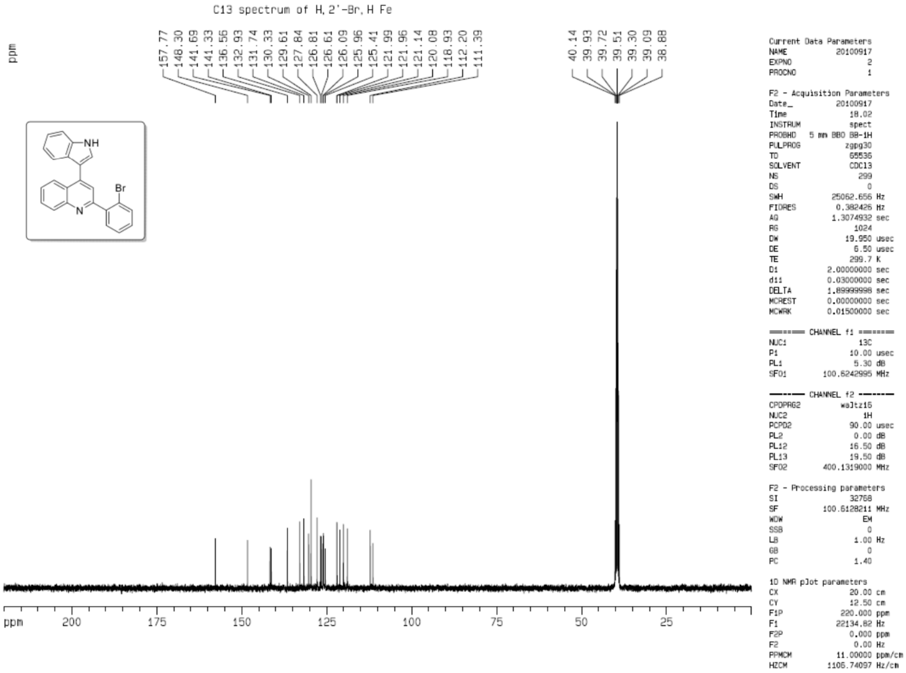

Figure S56. ^{13}C-NMR of 2-(2-Bromophenyl)-4-(1*H*-indol-3-yl)quinoline (**4f**).

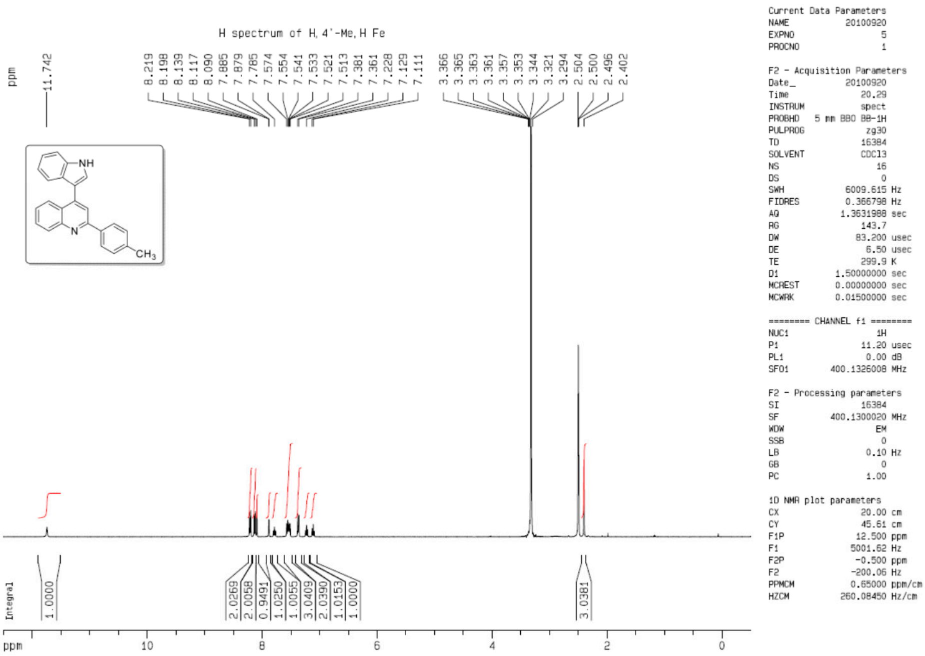

Figure S57. ^{1}H-NMR of 4-(1*H*-Indol-3-yl)-2-p-tolylquinoline (**4g**).

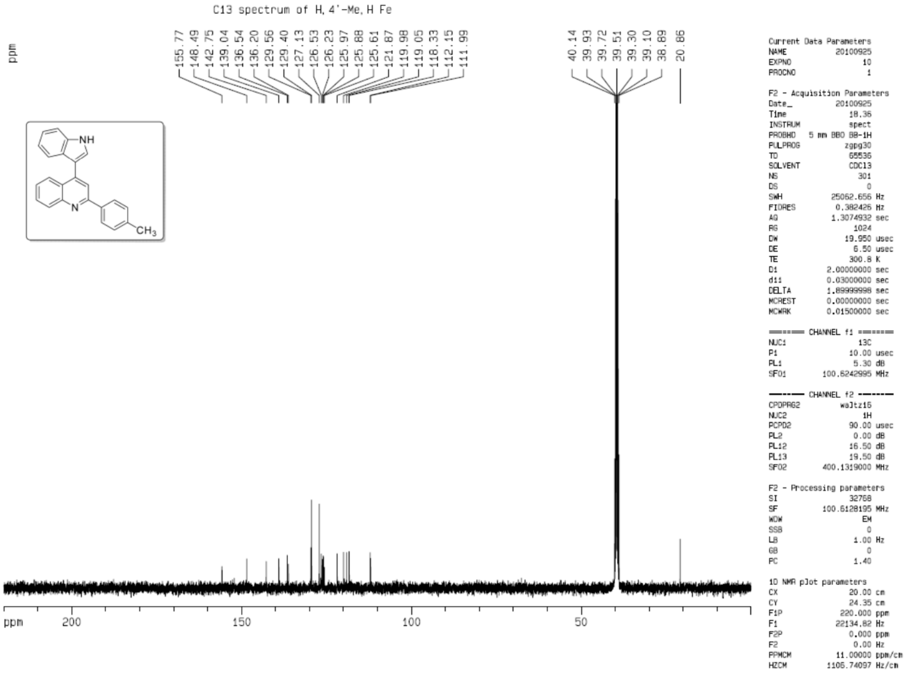

Figure S58. ^{13}C-NMR of 4-(1*H*-Indol-3-yl)-2-p-tolylquinoline (**4g**).

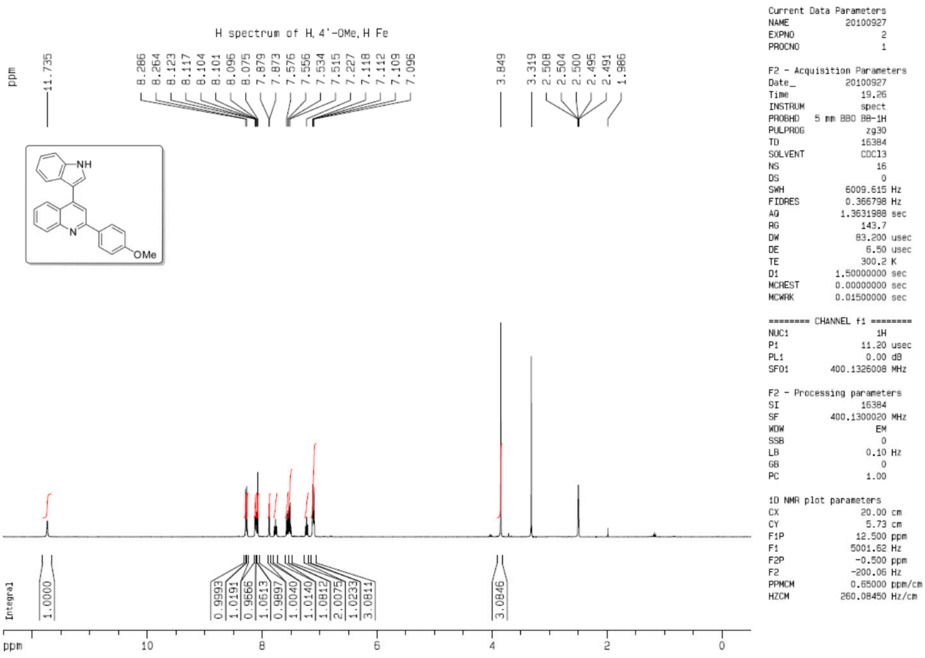

Figure S59. ^{1}H-NMR of 4-(1*H*-Indol-3-yl)-2-(4-methoxyphenyl)quinoline (**4h**).

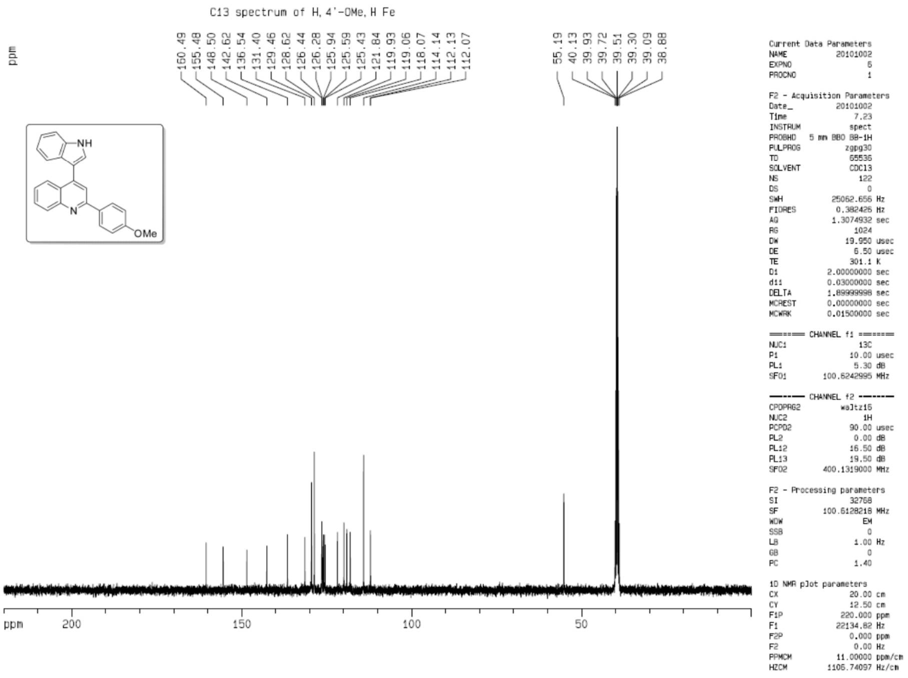

Figure S60. ^{13}C-NMR of 4-(1*H*-Indol-3-yl)-2-(4-methoxyphenyl)quinoline (**4h**).

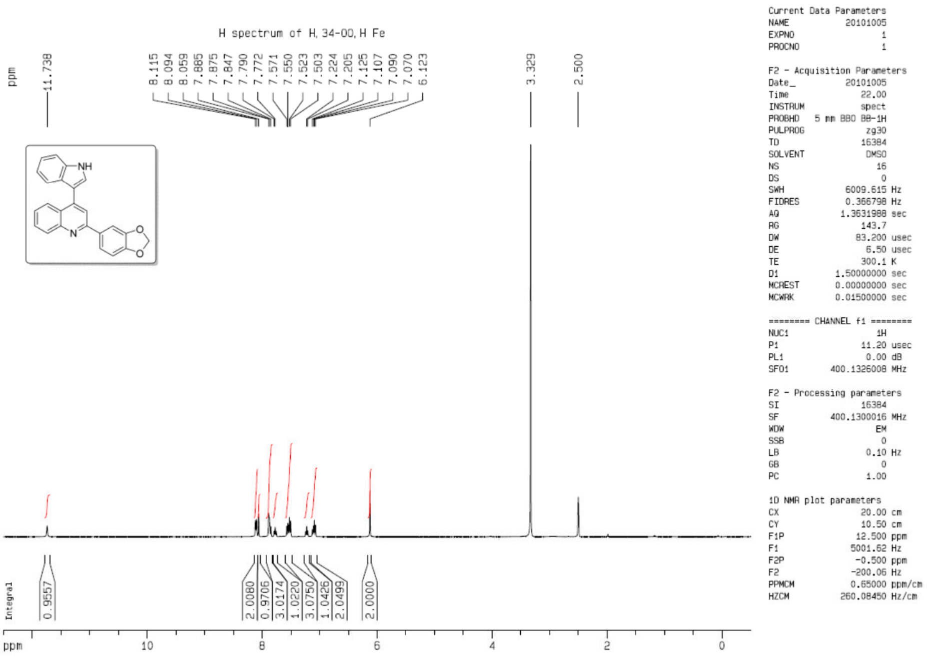

Figure S61. ^{1}H-NMR of 2-(Benzo[d][1,3]dioxol-5-yl)-4-(1*H*-indol-3-yl)quinoline (**4i**).

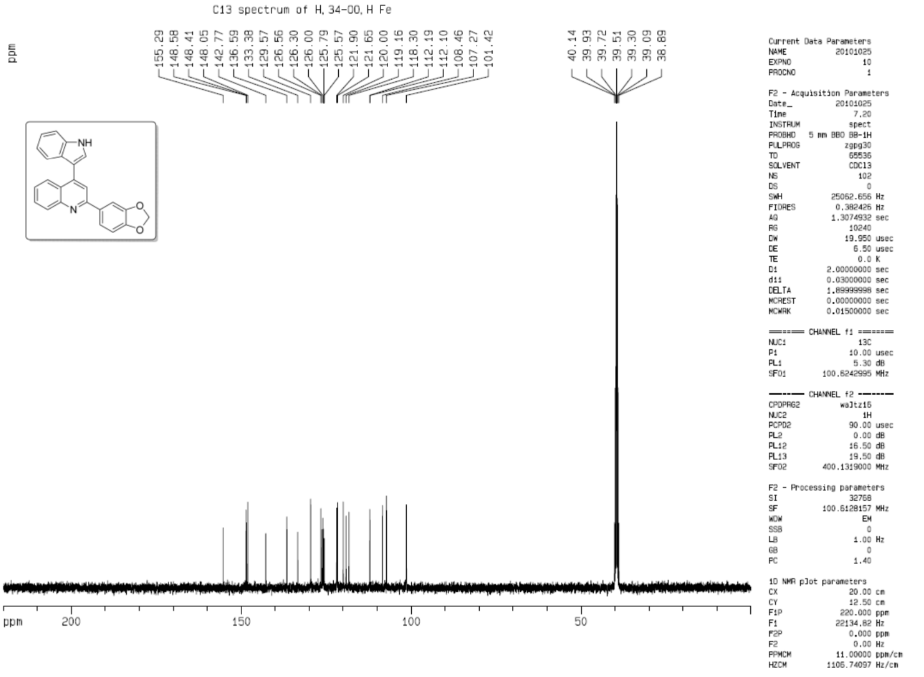

Figure S62. ^{13}C-NMR of 2-(Benzo[d][1,3]dioxol-5-yl)-4-(1*H*-indol-3-yl)quinoline (**4i**).

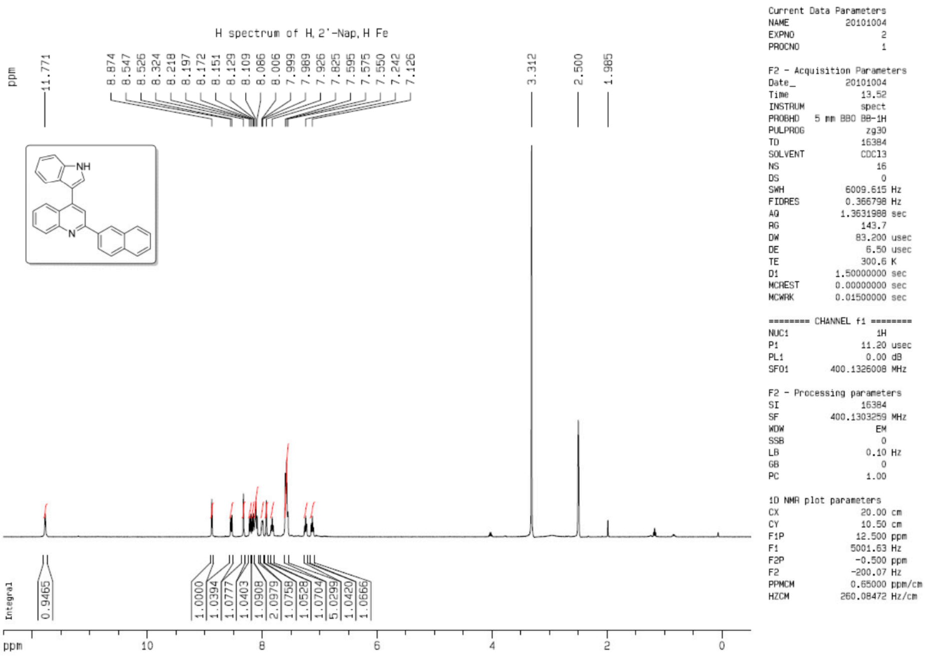

Figure S63. ^{1}H-NMR of 4-(1*H*-Indol-3-yl)-2-(naphthalen-2-yl)quinoline (**4j**).

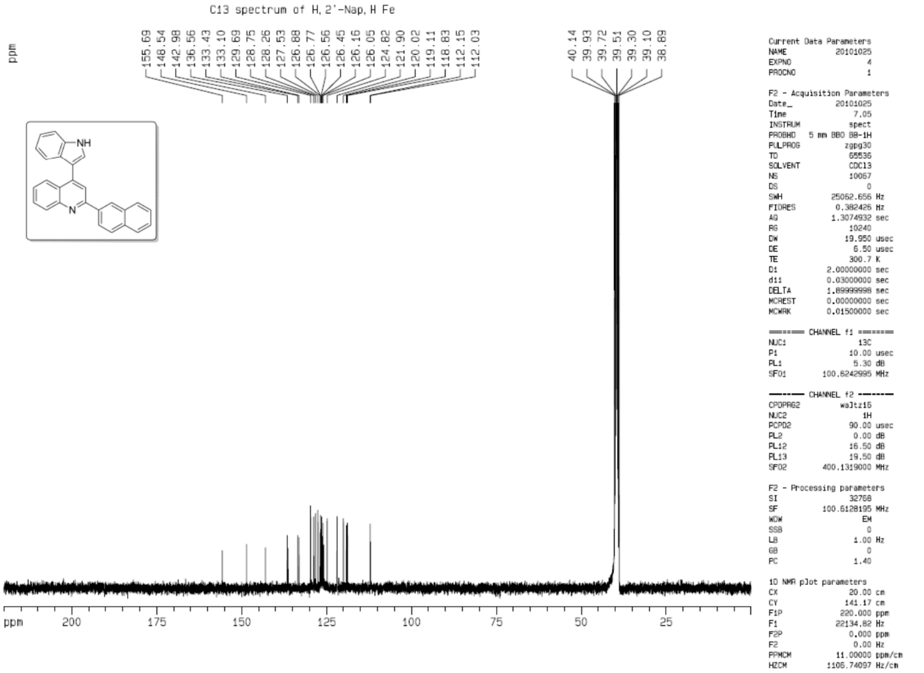

Figure S64. ^{13}C-NMR of 4-(1*H*-Indol-3-yl)-2-(naphthalen-2-yl)quinoline (**4j**).

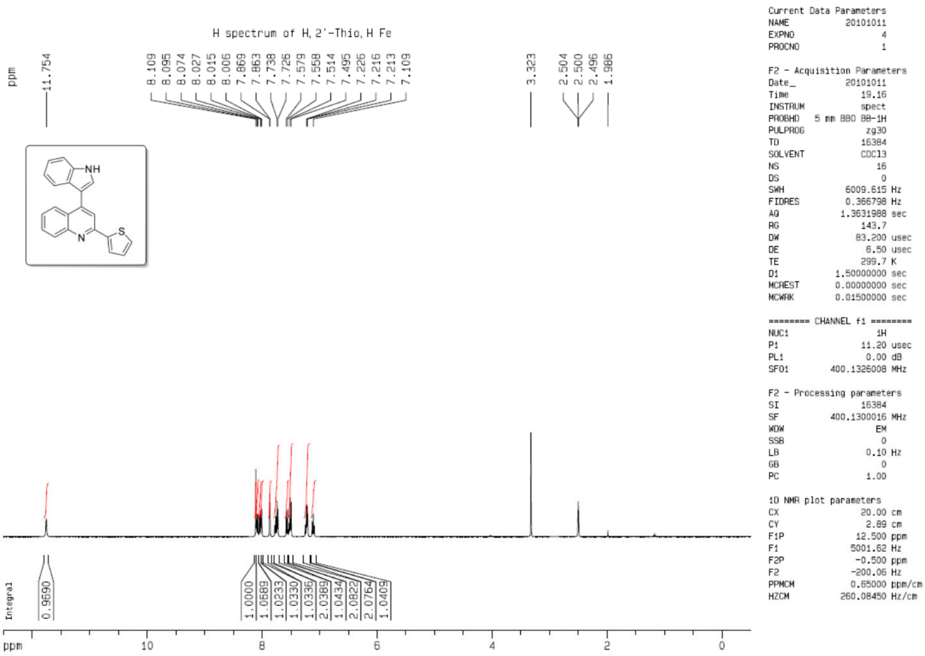

Figure S65. ^{1}H-NMR of 4-(1*H*-Indol-3-yl)-2-(thiophen-2-yl)quinoline (**4k**).

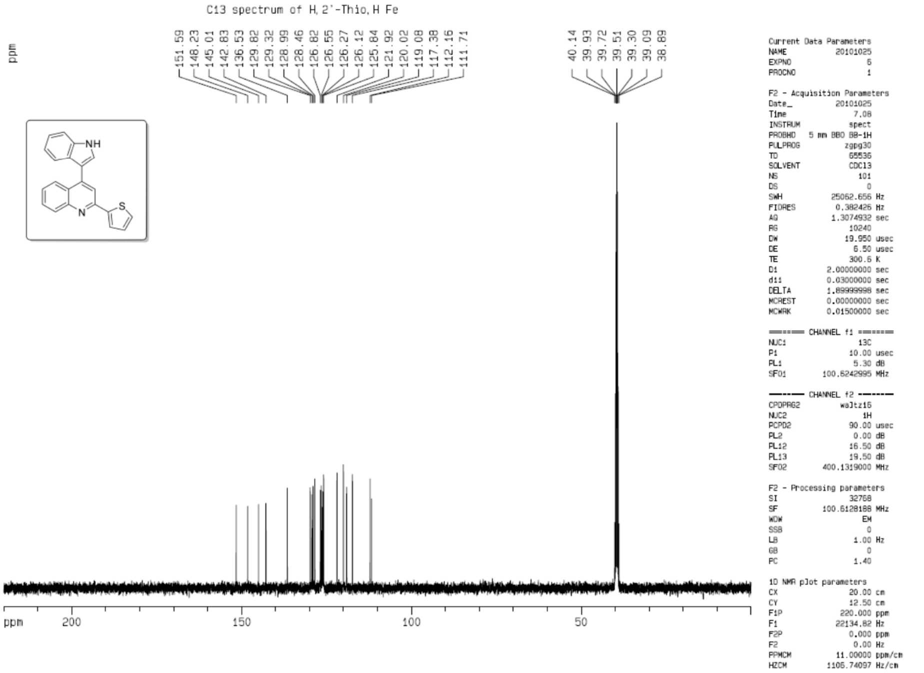

Figure S66. ^{13}C-NMR of 4-(1*H*-Indol-3-yl)-2-(thiophen-2-yl)quinoline (**4k**).

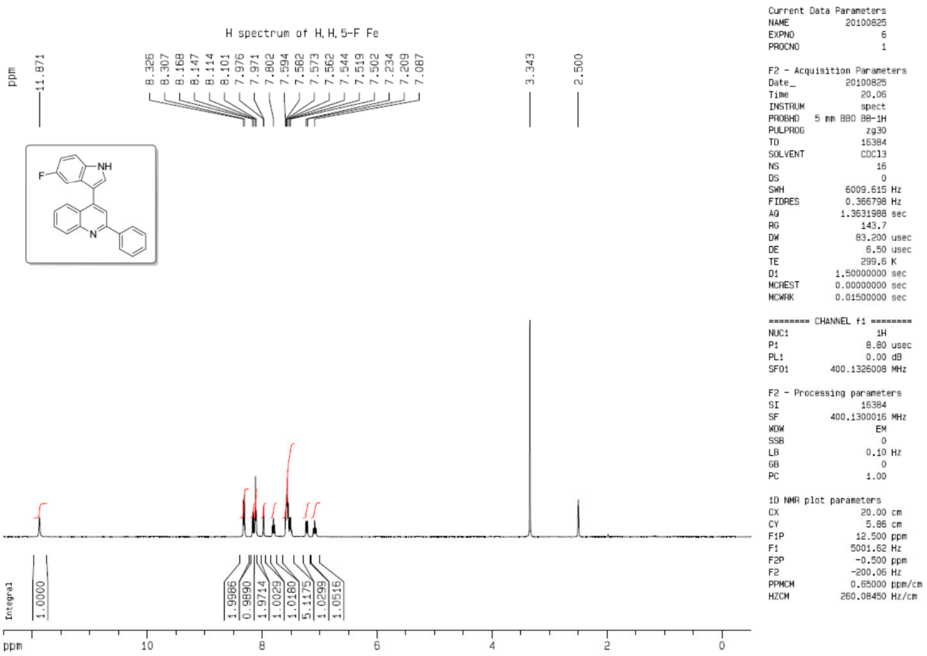

Figure S67. ^{1}H-NMR of 4-(5-Fluoro-1*H*-indol-3-yl)-2-phenylquinoline (**4l**).

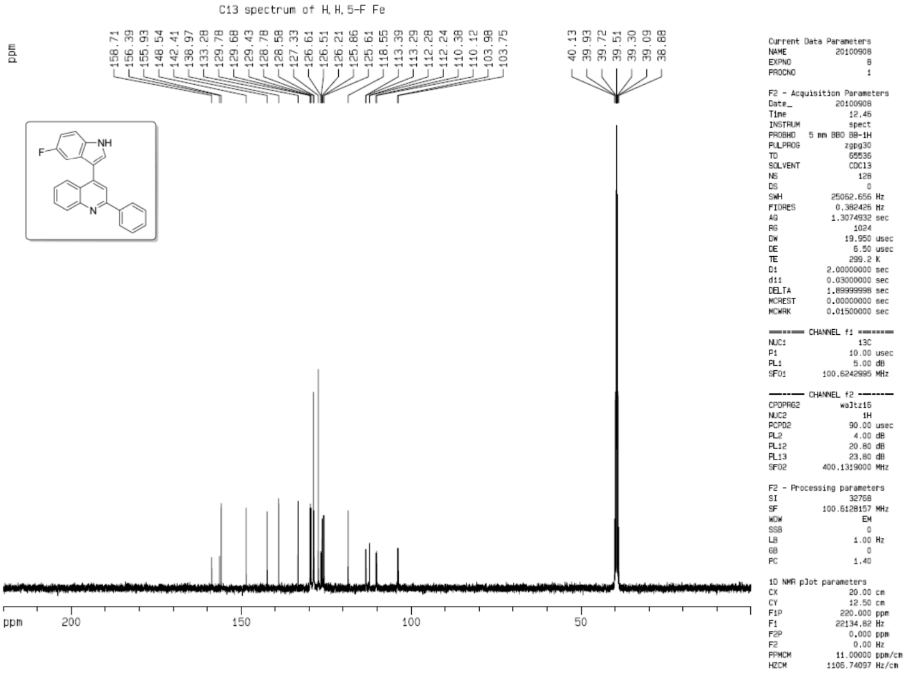

Figure S68. ^{13}C-NMR of 4-(5-Fluoro-1*H*-indol-3-yl)-2-phenylquinoline (**4l**).

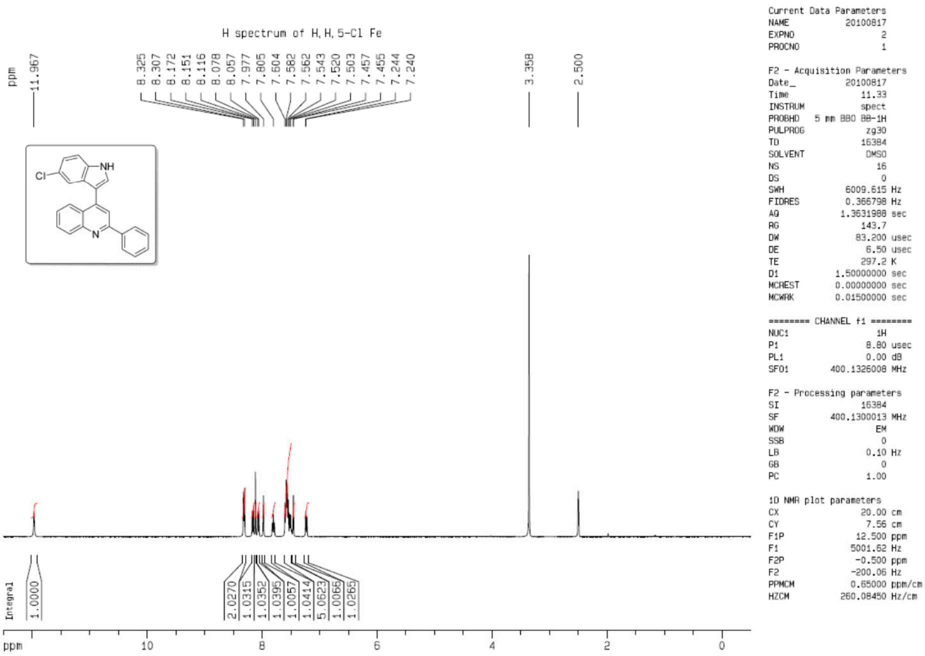

Figure S69. ^{1}H-NMR of 4-(5-Chloro-1*H*-indol-3-yl)-2-phenylquinoline (**4m**).

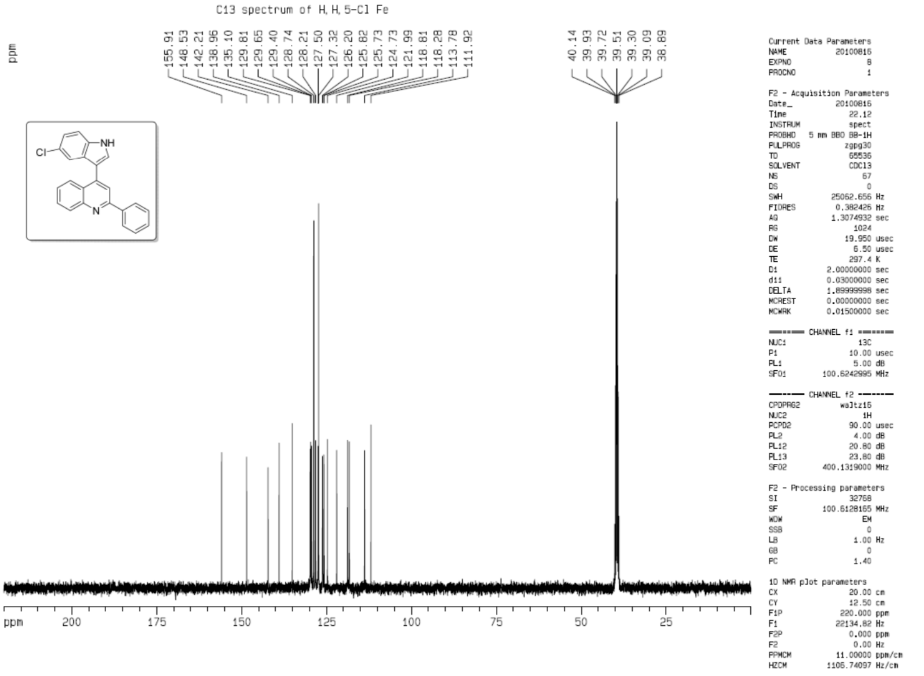

Figure S70. ^{13}C-NMR of 4-(5-Chloro-1*H*-indol-3-yl)-2-phenylquinoline (**4m**).

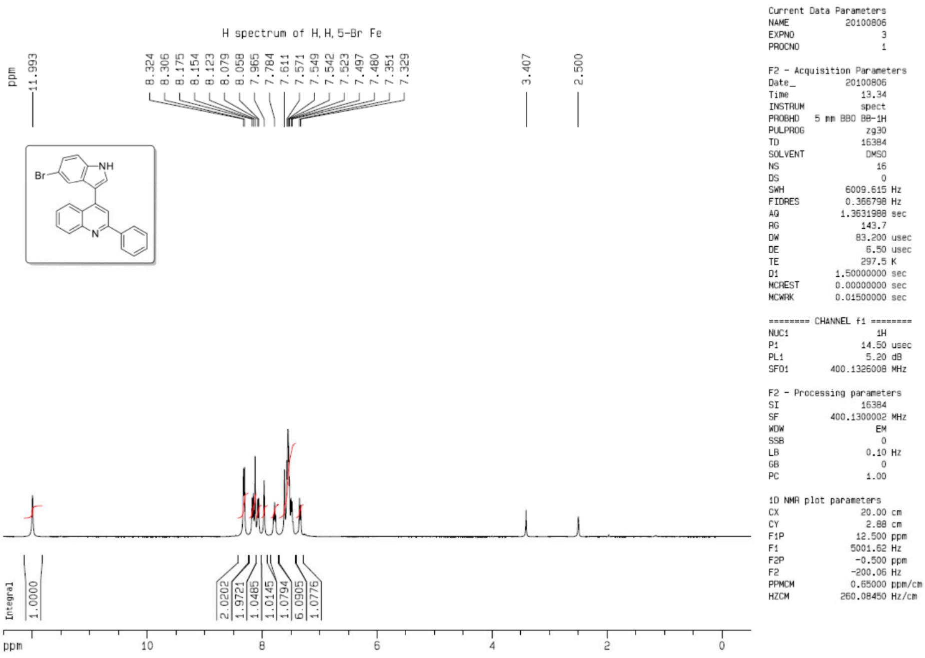

Figure S71. ^{1}H-NMR of 4-(5-Bromo-1*H*-indol-3-yl)-2-phenylquinoline (**4n**).

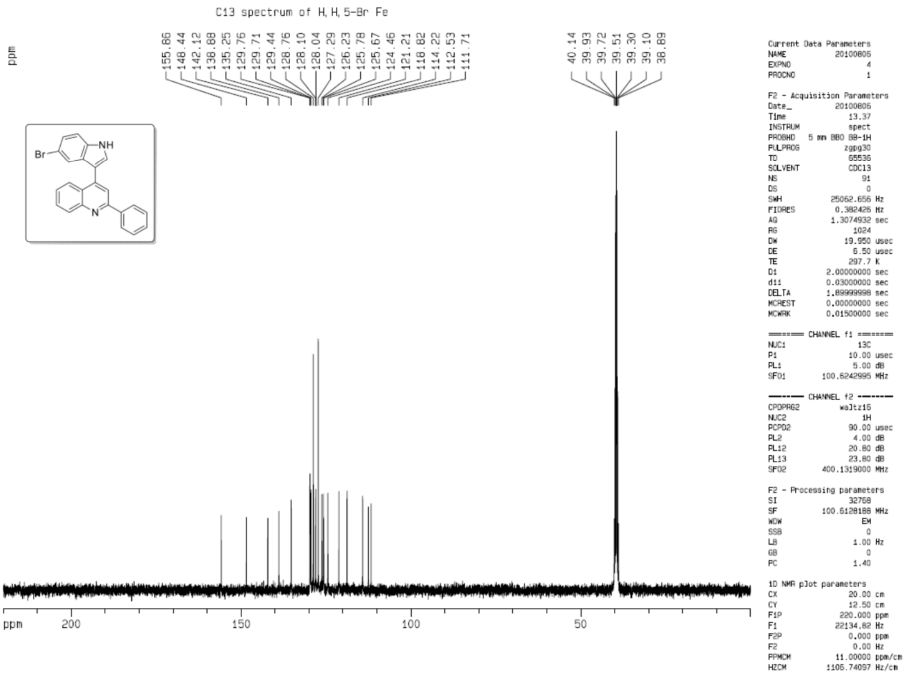

Figure S72. ^{13}C-NMR of 4-(5-Bromo-1*H*-indol-3-yl)-2-phenylquinoline (**4n**).

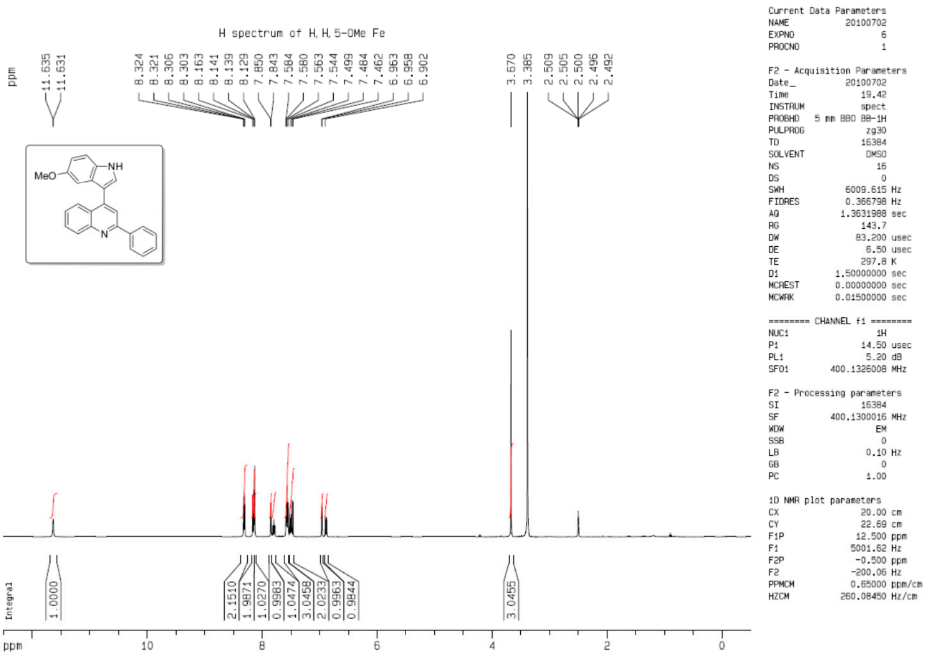

Figure S73. ^{1}H-NMR of 4-(5-Methoxy-1*H*-indol-3-yl)-2-phenylquinoline (**4o**).

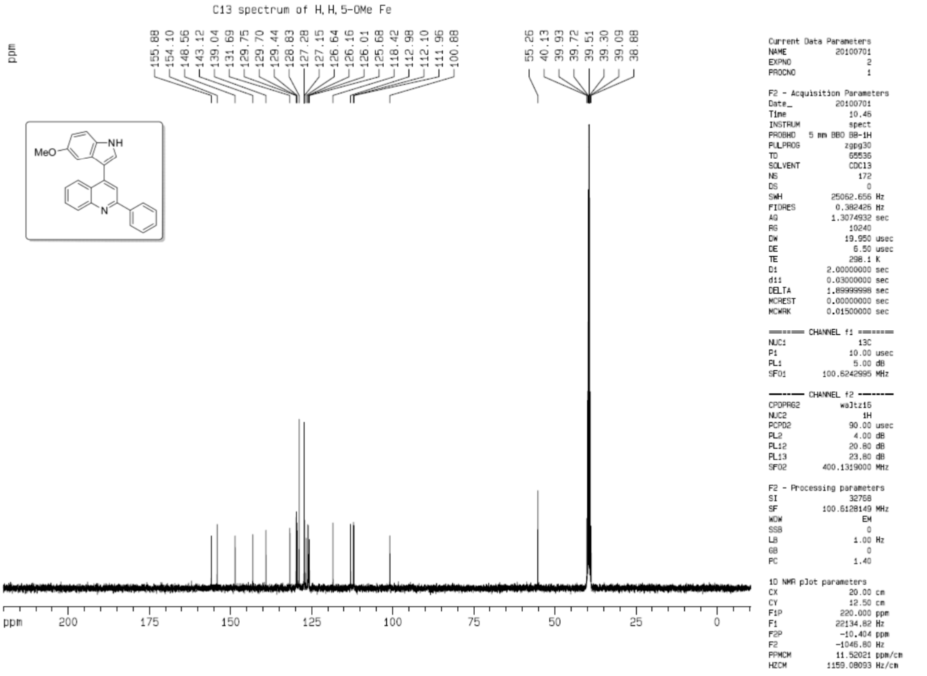

Figure S74. ^{13}C-NMR of 4-(5-Methoxy-1*H*-indol-3-yl)-2-phenylquinoline (**4o**).

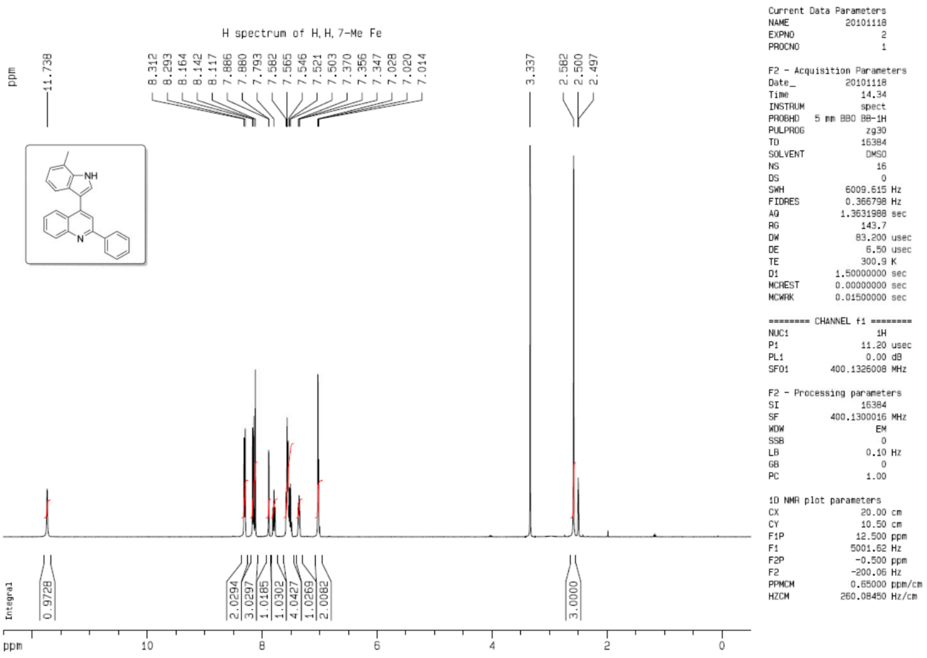

Figure S81. ^{1}H-NMR of 4-(7-Methyl-1*H*-indol-3-yl)-2-phenylquinoline (**4p**).

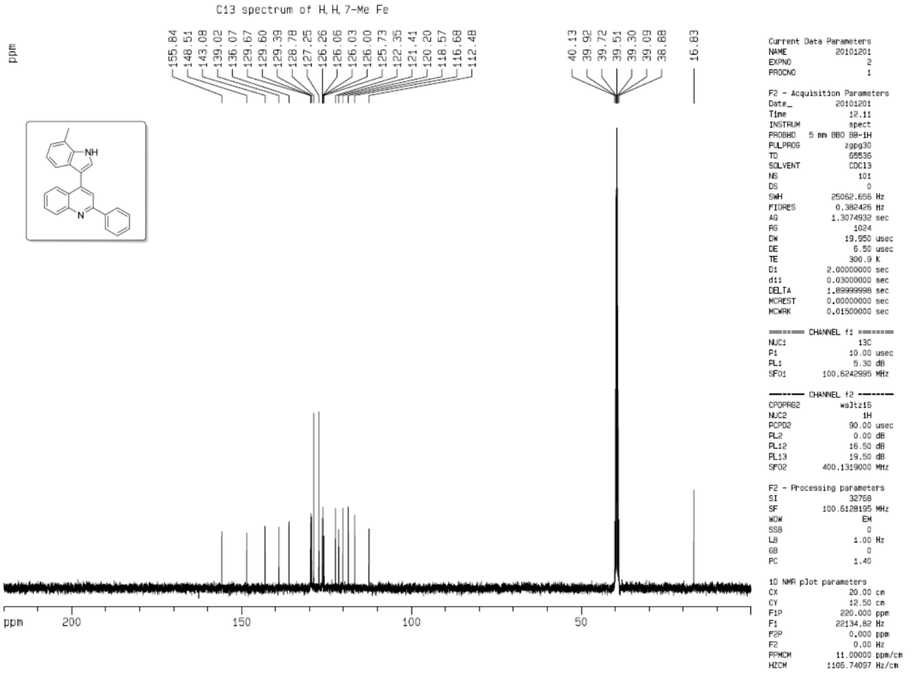

Figure S76. ^{13}C-NMR of 4-(7-Methyl-1*H*-indol-3-yl)-2-phenylquinoline (**4p**).

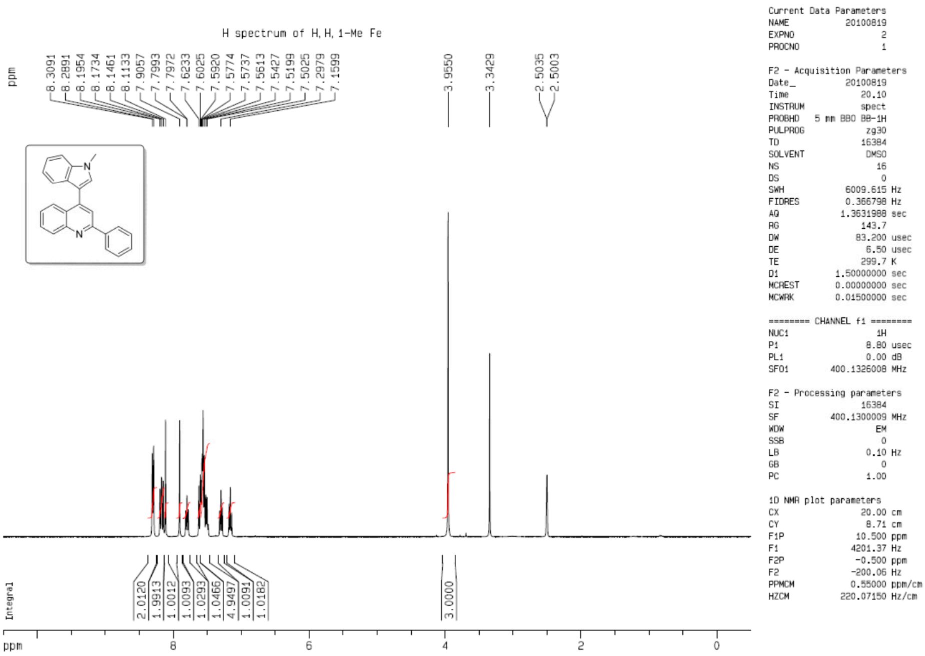

Figure S77. ^{1}H-NMR of 4-(1-Methyl-1*H*-indol-3-yl)-2-phenylquinoline (**4q**).

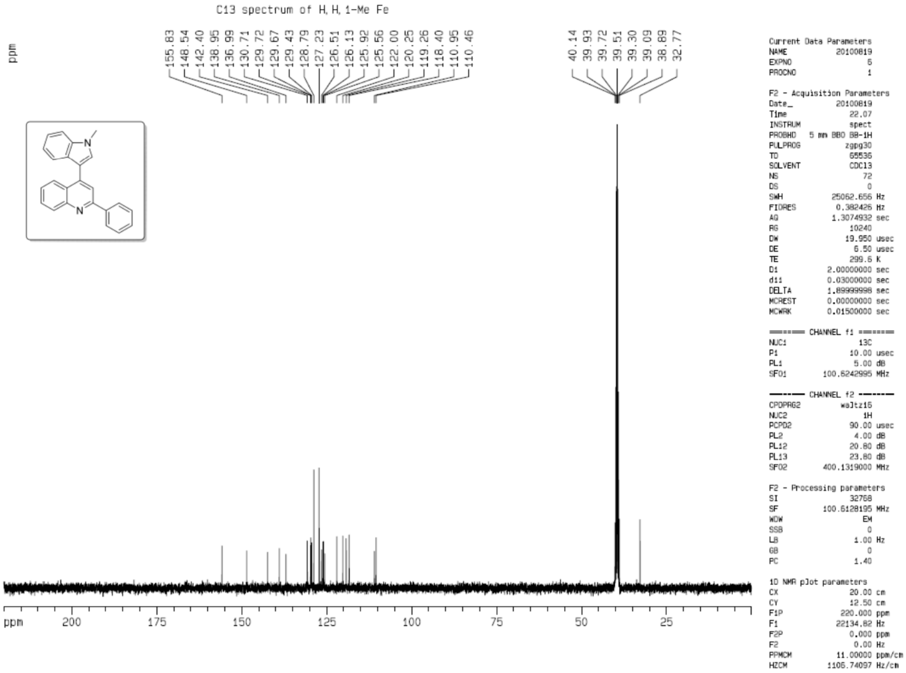

Figure S78. ^{13}C-NMR of 4-(1-Methyl-1*H*-indol-3-yl)-2-phenylquinoline (**4q**).

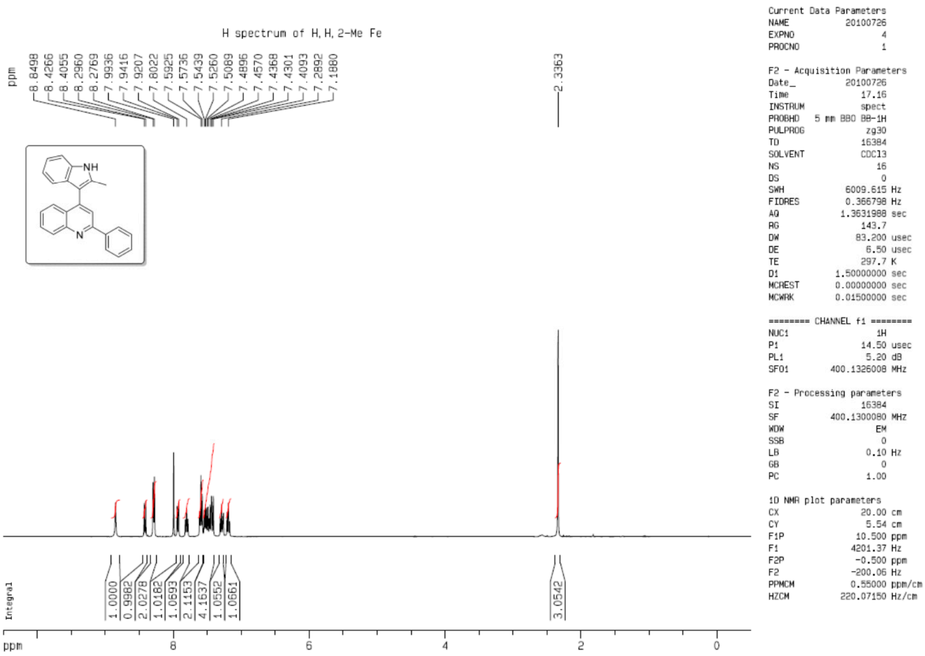

Figure S79. ^{1}H-NMR of 4-(2-Methyl-1*H*-indol-3-yl)-2-phenylquinoline (**4r**).

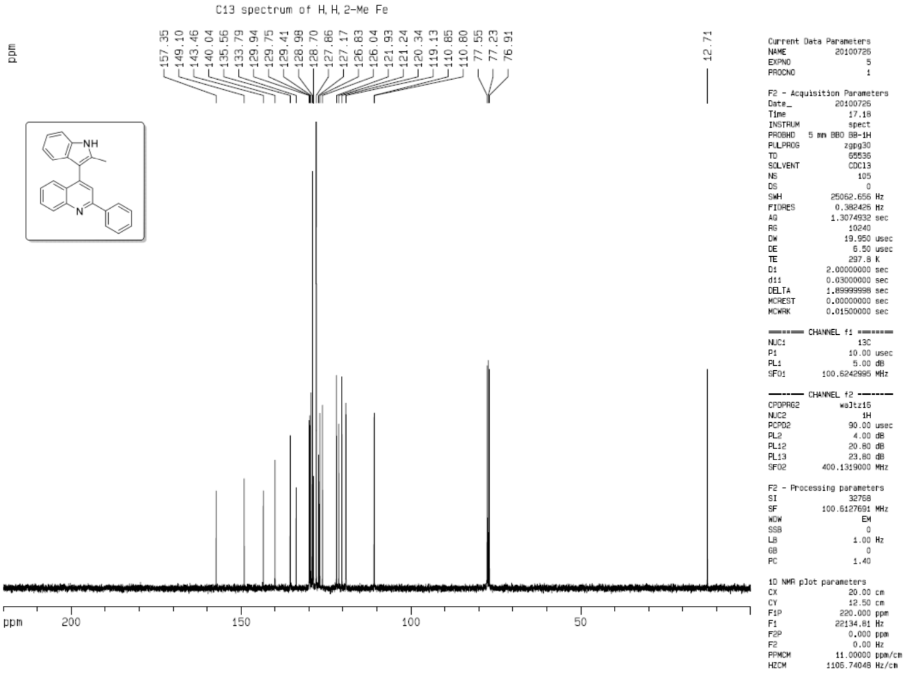

Figure S80. ^{13}C-NMR of 4-(2-Methyl-1*H*-indol-3-yl)-2-phenylquinoline (**4r**).

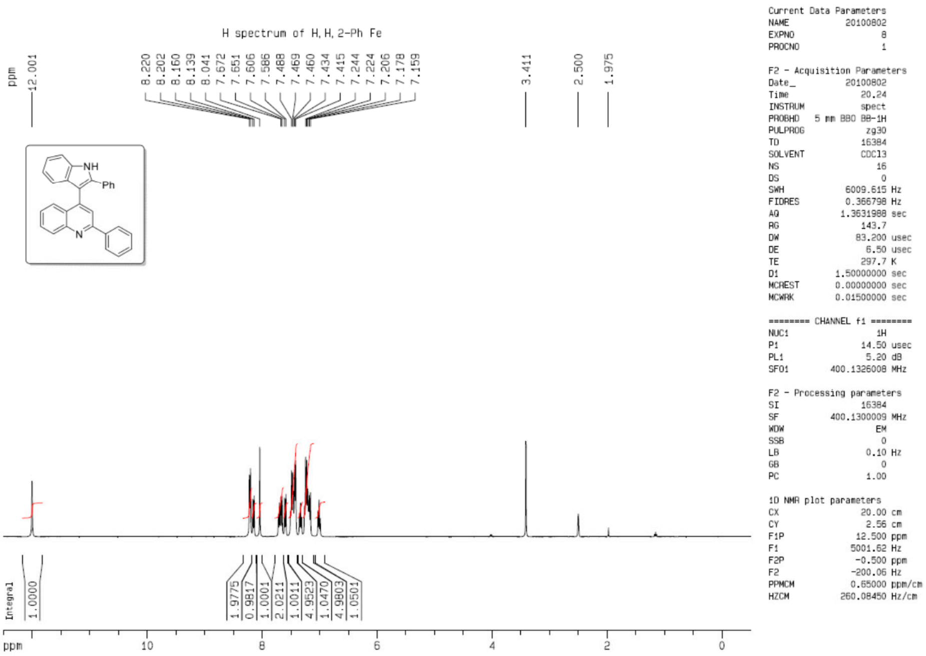

Figure S81. ^{1}H-NMR of 2-Phenyl-4-(2-phenyl-1*H*-indol-3-yl)quinoline (**4s**).

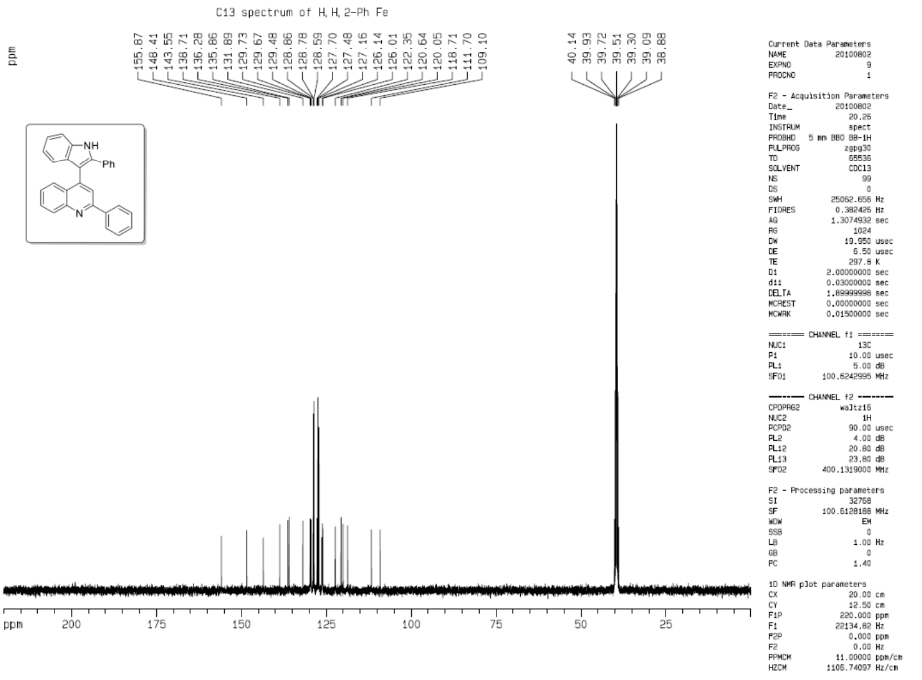

Figure S82. ^{13}C-NMR of 2-Phenyl-4-(2-phenyl-1*H*-indol-3-yl)quinoline (**4s**).

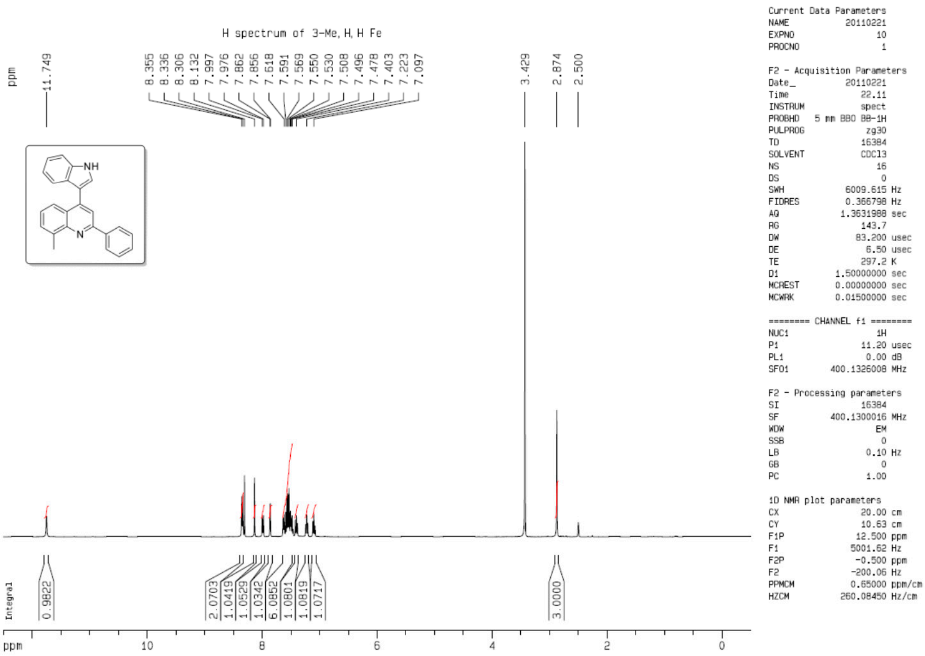

Figure S83. ^{1}H-NMR of 4-(1*H*-Indol-3-yl)-8-methyl-2-phenylquinoline (**4t**).

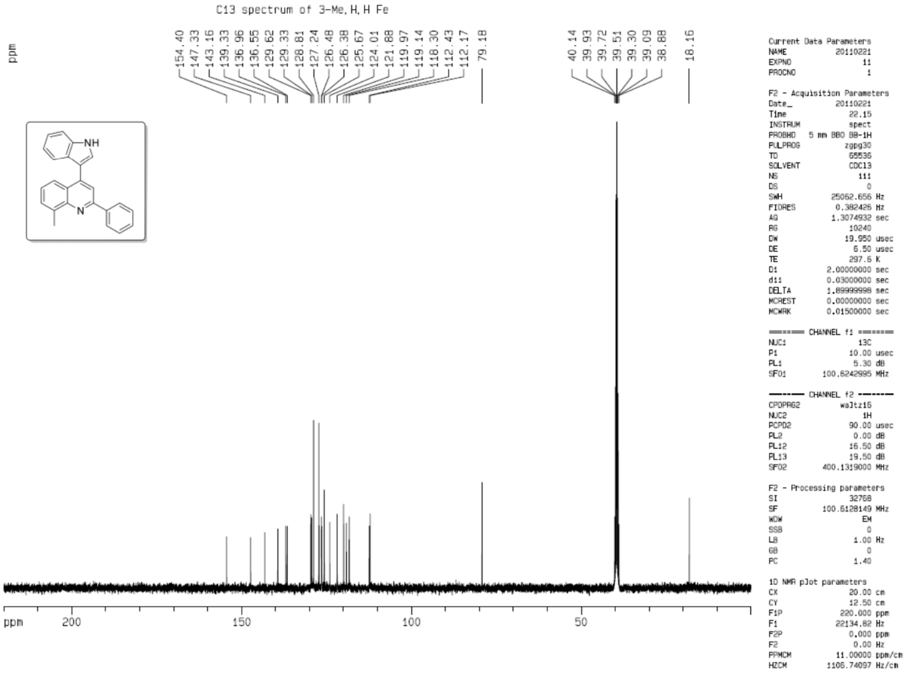

Figure S84. ^{13}C-NMR of 4-(1*H*-Indol-3-yl)-8-methyl-2-phenylquinoline (**4t**).

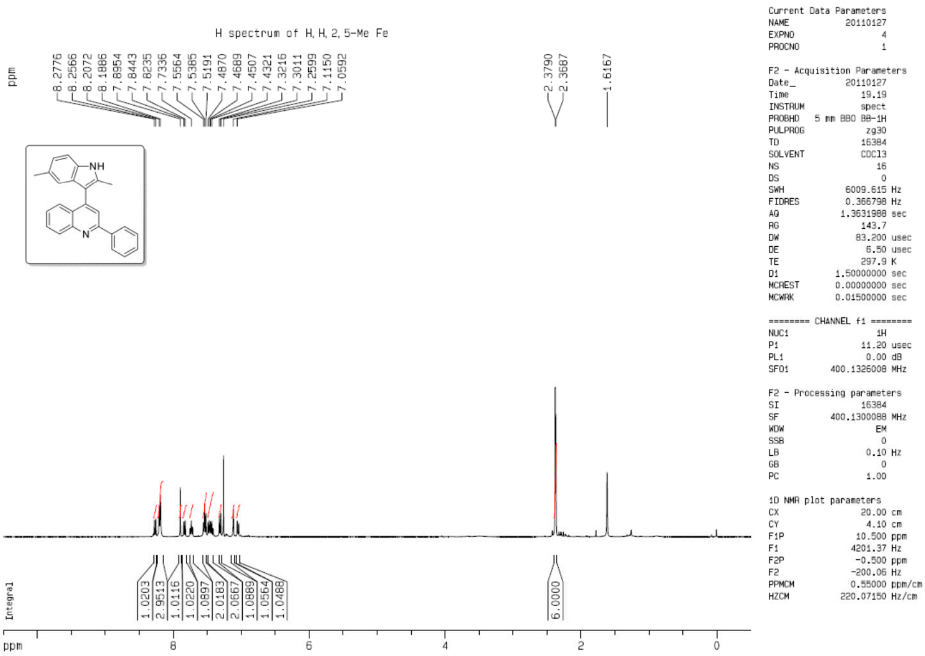

Figure S85. ^{1}H-NMR of 4-(2,5-Dimethyl-1*H*-indol-3-yl)-2-phenylquinoline (**4u**).

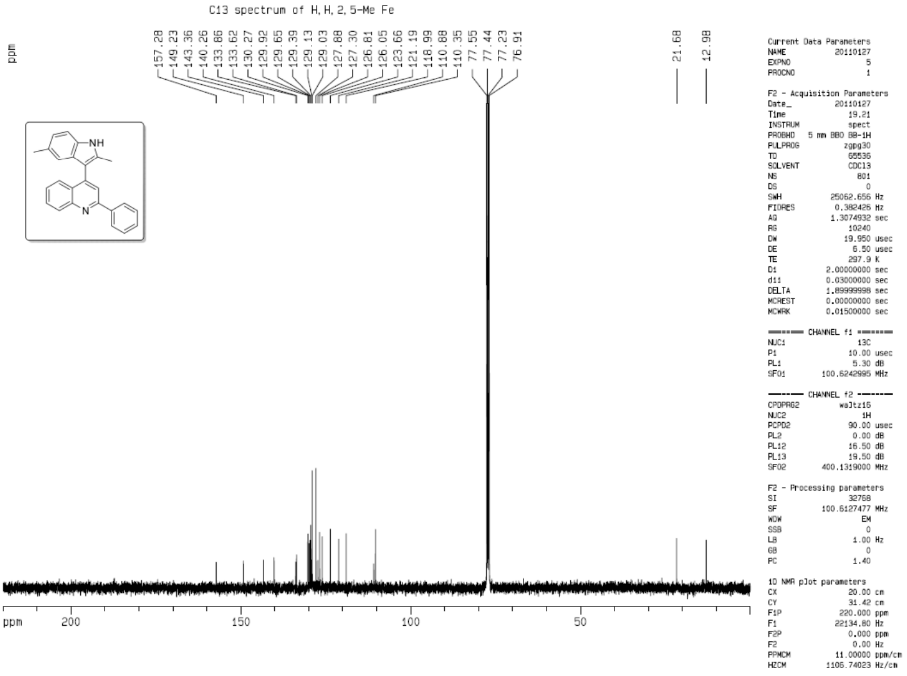

Figure S86. ^{13}C-NMR of 4-(2,5-Dimethyl-1*H*-indol-3-yl)-2-phenylquinoline (**4u**).

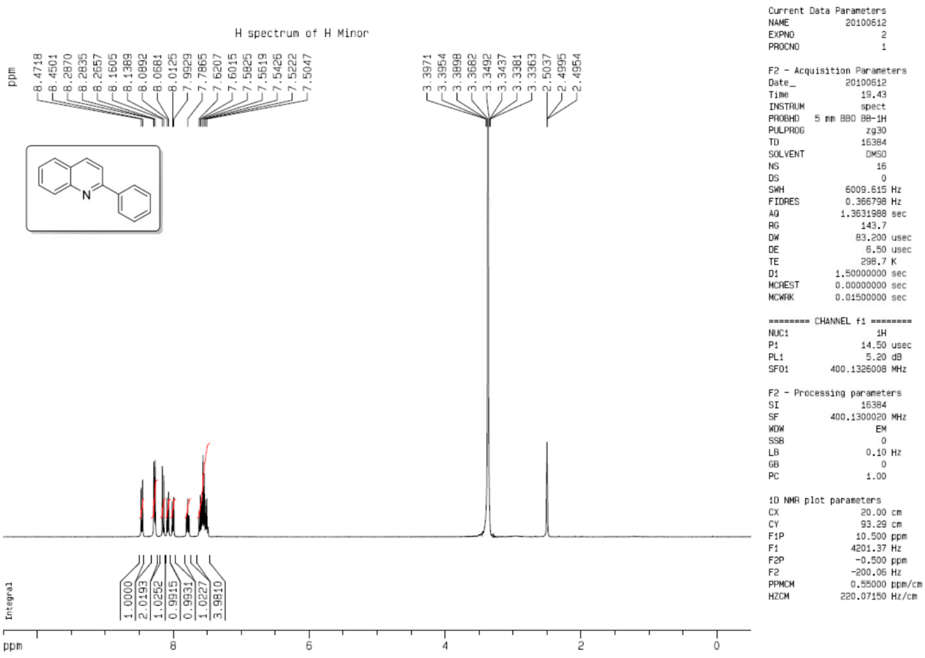

Figure S87. ^{13}C-NMR of 2-Phenylquinoline (**5a**).

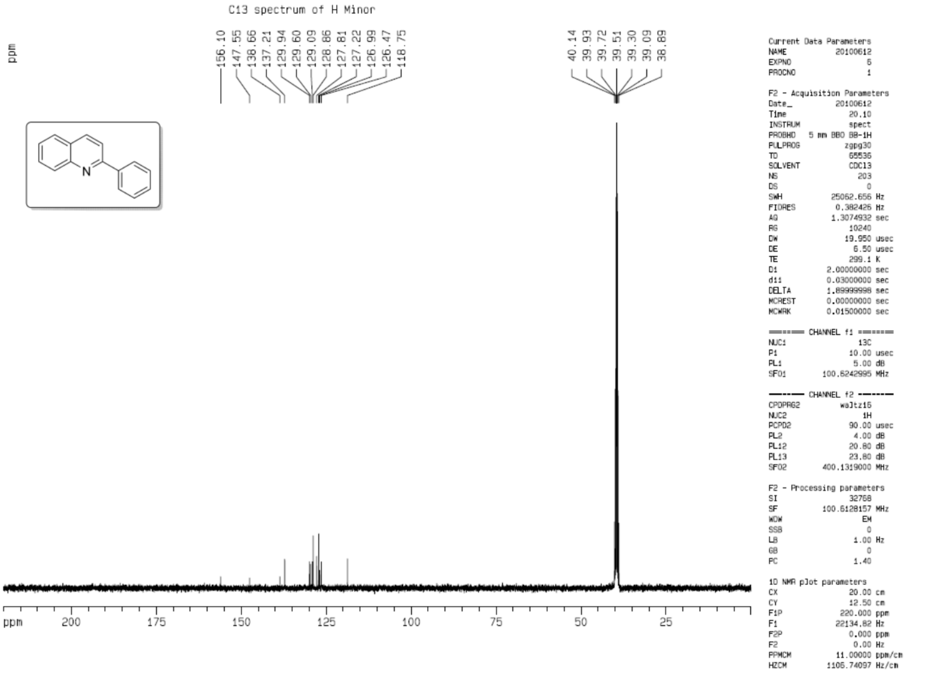

Figure S88. ^{13}C-NMR of 2-Phenylquinoline (**5a**).

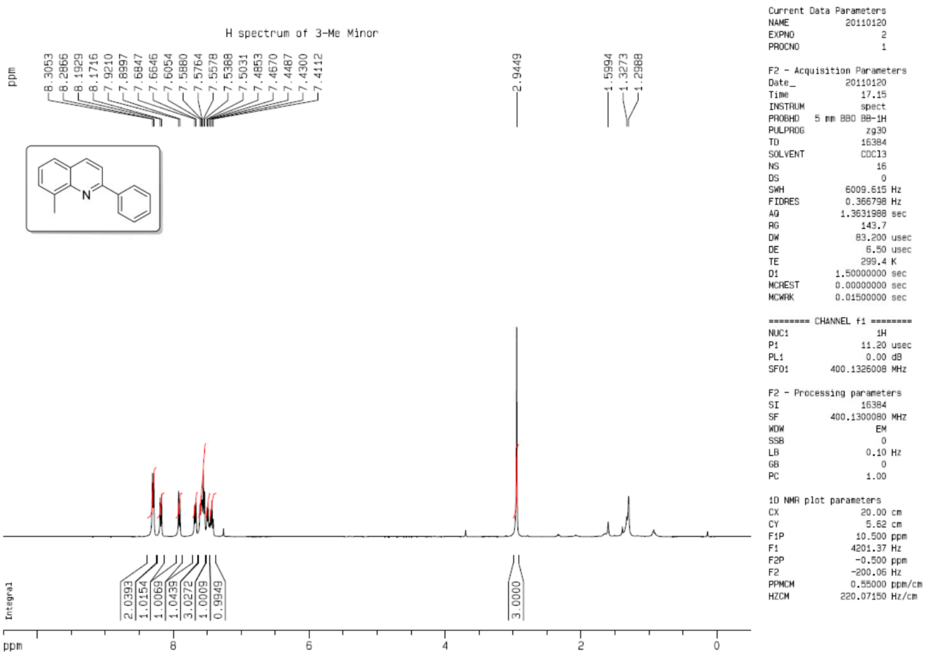

Figure S89. ^{1}H-NMR of 8-Methyl-2-phenylquinoline (**5v**).

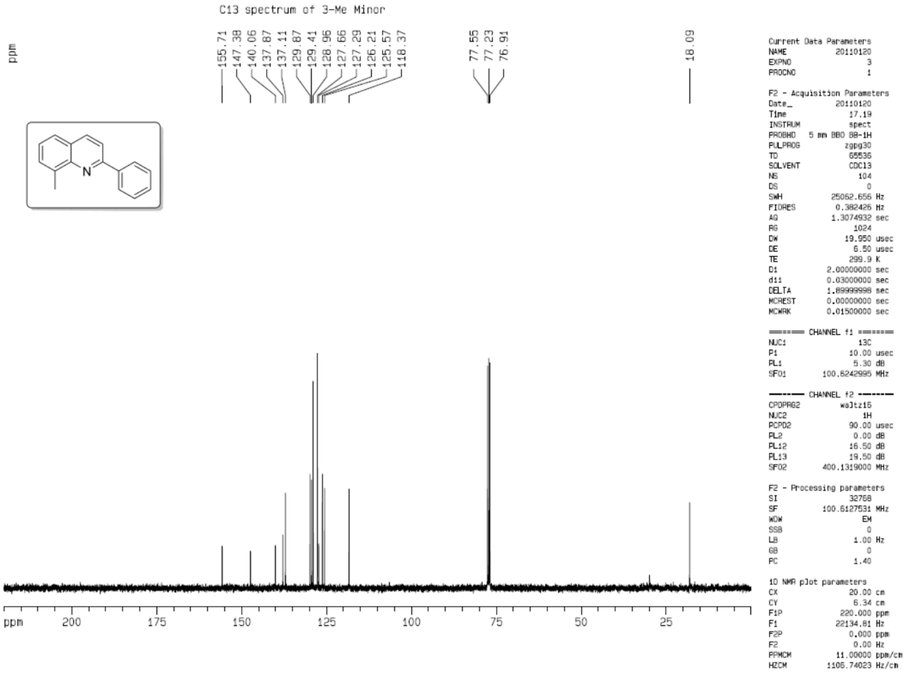

Figure S90. ^{13}C-NMR of 8-Methyl-2-phenylquinoline (**5v**).
